# Nickel-Catalyzed 8‑Endo Cyclization/Carbonylation for the Synthesis of Eight-Membered Lactams

**DOI:** 10.1021/acs.orglett.6c02643

**Published:** 2026-06-29

**Authors:** Yibin He, Zilin Huang, Yuting Jiang, Hucheng Ma, Xinxin Qi, Xiao-Feng Wu

**Affiliations:** † School of Chemistry and Chemical Engineering, Key Laboratory of Surface & Interface Science of Polymer Materials of Zhejiang Province, 12646 Zhejiang Sci-Tech University, Hangzhou, Zhejiang 310018, People’s Republic of China; ‡ Dalian National Laboratory for Clean Energy, Dalian Institute of Chemical Physics, Chinese Academy of Sciences, 116023, Dalian, Liaoning, People’s Republic of China; § Leibniz-Institut für Katalyse e.V., Albert-Einstein-Straße 29a, Rostock 18059, Germany

## Abstract

Medium-sized lactams are prevalent motifs in many bioactive molecules and natural products. However, their construction remains challenging due to unfavorable enthalpic and entropic barriers during the transition state. Herein, a novel nickel-catalyzed 8-endo cyclization/carbonylation has been achieved for the synthesis of eight-membered lactams bearing a gem-difluoro group. This approach features high regioselectivity, good functional group tolerance, and CO gas-free operation, which provides a good supplement for the preparation of eight-membered lactams via a nickel-catalyzed carbonylation process.

## Graphical abstract

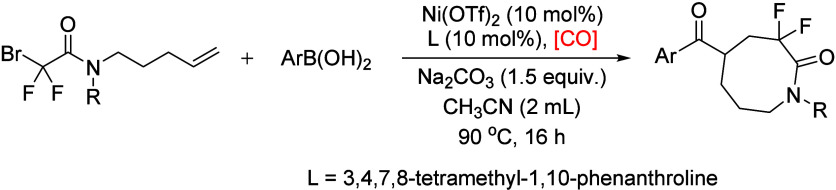

Medium-sized lactams are widespread structural motifs in a variety of natural products and pharmaceuticals.^1^ The distinctive structure of these compounds can facilitate the formation of high-affinity, high-selectivity interactions with biological targets and make it easier to optimize pharmacokinetic properties in drug design. In particular, eight-membered lactams, which are pharmaceutically important core skeletons, play a pivotal role in drug discovery (Figure 1).^2^ For example, balasubramide, a natural product isolated from the leaves of the Sri Lankan plant *Clausena indica*, shows anti-neuroinflammatory properties.^3^ Decursivine, an indole alkaloid isolated from the leaves and stems of the plant *Rhaphid ophora decursiva*, serves as a lead compound for antimalarial drug discovery.^4^ In this regard, the exploration of general and efficient strategies to access eight-membered lactams is greatly needed. However, eight-membered lactams are synthetically more challenging owing to the unfavorable enthalpic and entropic barriers in their transition states.^5^ Typically, the synthesis of eight-membered lactams mainly relies on ring-expansion reactions,^6^ while alternative approaches such as ring-closing metathesis,^7^ Claisen rearrangement,^8^ and other methods^9^ have also been developed. Although substantial progress has been made, these methods still suffer from some drawbacks, such as harsh reaction conditions, complicated substrate preparation, and narrow substrate scope. Therefore, the development of concise and practical routes toward eight-membered lactams continues to be highly desirable. In recent decades, the transition-metal-catalyzed carbonylation reaction has become a powerful tool for the construction of carbonyl-containing compounds.^10^ Among these transformations, the nickel-catalyzed carbonylation reaction has received extensive attention due to the low cost and abundant properties of nickel in contrast to noble metals like palladium, rhodium, and iridium. Nevertheless, the formation of volatile and toxic Ni­(CO)_4_ limited its application.^11^ To overcome this disadvantage, the utility of low-pressure CO and CO surrogates provides a good option.^12^ Consequently, nickel-catalyzed carbonylation under mild conditions has emerged as a promising strategy for constructing medium-sized lactams, including the challenging eight-membered ring systems.

**Figure 1:**
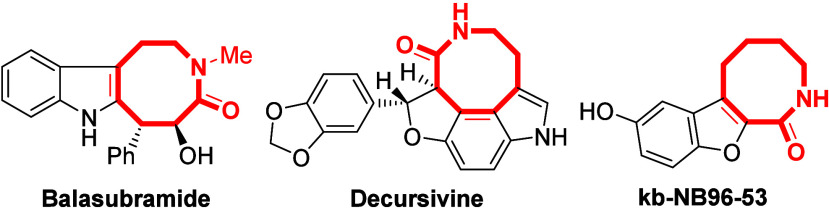
Representative 8-membered lactams in natural products and bioactive molecules.

Over the last few decades, fluorine-containing compounds have attracted considerable attention in the pharmaceutical and material fields.^13^ Fluorinated drugs usually exhibit distinctive biological activity and biocompatibility because fluorine atoms could serve as highly effective biosynthetic isosteres, which can precisely modulate the metabolic stability or lipophilicity of lead compounds. In particular, heterocycles bearing a gem-difluoro group have been extensively studied because of their pharmacokinetic properties and biological activities.^14^ Therefore, the incorporation of a gem-difluoro group into heterocycles is of great interest in drug design and exploration.

Most recently, our group developed the synthesis of functionalized lactams through nickel-catalyzed 5-exo and 6-exo cyclization/carbonylation reactions, respectively.^15^ Notably, compared to 5-exo or 6-exo cyclization, the type of 8-endo cyclization remains less studied, while competing 7-exo cyclization also exists. In our continuous interest in the nickel-catalyzed cyclization/carbonylation reactions, by using bromodifluoroacetamides **1** as the starting materials, we anticipated that the 8-endo cyclization/carbonylation might be achieved as the kinetically favorable electrophilic addition of the reactive transient difluoromethyl radical to the alkene would generate a relatively stable secondary radical, thereby providing a driving force for the transformation. Herein, we wish to report a nickel-catalyzed 8-endo cyclization/carbonylation reaction for the synthesis of eight-membered lactams bearing a gem-difluoro group (Scheme 1).

**Scheme 1:**
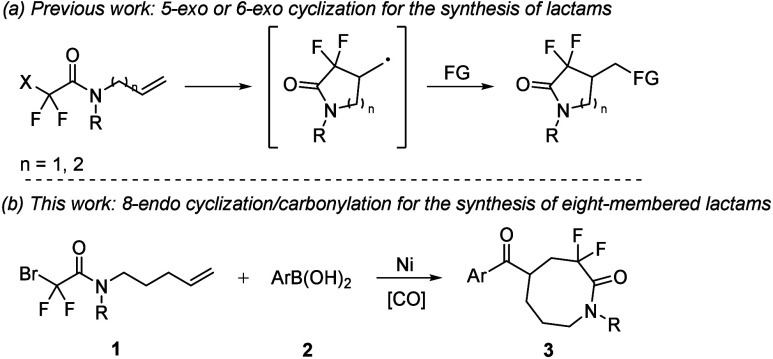
Synthesis of Lactams

A preliminary investigation was performed with 2-bromo-2,2-difluoro-*N*-(pent-4-en-1-yl)-*N*-phenylacetamide **1a** and *p*-tolylboronic acid **2b** as the model substrates to screen the reaction conditions. To our delight, the desired product **3ab** was obtained in 40% yield by applying Ni­(OTf)_2_ as the catalyst, L1 as the ligand, and Na_2_CO_3_ as the base in 1,4-dioxane at 90 °C for 16 h (Table 1, entry 1). Encouraged by this result, several solvents such as THF, DMF, DMA, and CH_3_CN were then studied (Table 1, entries 2–5); CH_3_CN tends to be the best solvent (Table 1, entry 5). Next, a series of catalysts were examined (Table 1, entries 6–10). Ni­(acac)_2_ failed to afford the final product, while other catalysts such as NiBr_2_·DME, NiI_2_, Ni­(dppe)_2_Cl_2_, and Ni­(PPh_3_)_2_Cl_2_ provided reduced yields of 29–61%. Next, the performance of ligand was tested (Table 1, entries 11–15); L2, L3, L4, and dtbbpy afforded the product **3ab** in 32–67% yields, whereas L5 gave the desired product in trace amount. Furthermore, the effect of base was investigated, and the yield of product **3ab** was decreased (Table 1, entries 16–19). Notably, no 7-exo cyclized product was detected during the optimization process.

**Table 1:**
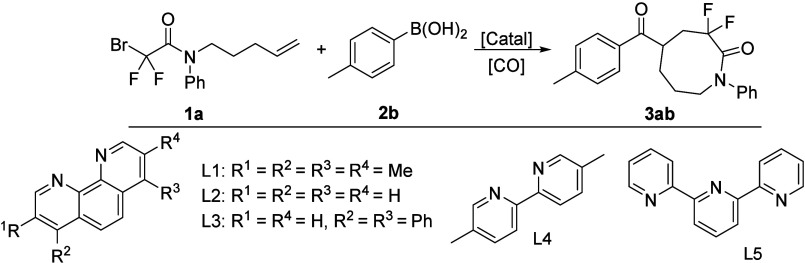
Screening of Reaction Conditions^a^

entry	Catal.	ligand	base	solvent	Yield (%)
1	Ni(OTf)_2_	L1	Na_2_CO_3_	1,4-dioxane	40
2	Ni(OTf)_2_	L1	Na_2_CO_3_	THF	31
3	Ni(OTf)_2_	L1	Na_2_CO_3_	DMF	37
4	Ni(OTf)_2_	L1	Na_2_CO_3_	DMA	42
5	Ni(OTf)_2_	L1	Na_2_CO_3_	CH_3_CN	77
6	NiBr_2_·DME	L1	Na_2_CO_3_	CH_3_CN	62
7	Ni(acac)_2_	L1	Na_2_CO_3_	CH_3_CN	0
8	NiI_2_	L1	Na_2_CO_3_	CH_3_CN	61
9	Ni(dppe)_2_Cl_2_	L1	Na_2_CO_3_	CH_3_CN	29
10	Ni(PPh_3_)_2_Cl_2_	L1	Na_2_CO_3_	CH_3_CN	54
11	Ni(OTf)_2_	L2	Na_2_CO_3_	CH_3_CN	64
12	Ni(OTf)_2_	L3	Na_2_CO_3_	CH_3_CN	61
13	Ni(OTf)_2_	L4	Na_2_CO_3_	CH_3_CN	67
14	Ni(OTf)_2_	dtbbpy	Na_2_CO_3_	CH_3_CN	32
15	Ni(OTf)_2_	L5	Na_2_CO_3_	CH_3_CN	trace
16	Ni(OTf)_2_	L1	K_2_CO_3_	CH_3_CN	trace
17	Ni(OTf)_2_	L1	Cs_2_CO_3_	CH_3_CN	0
18	Ni(OTf)_2_	L1	NaHCO_3_	CH_3_CN	29
19	Ni(OTf)_2_	L1	Na_3_PO_4_	CH_3_CN	58

a Reaction conditions: **1a** (0.2 mmol), **2b** (0.3 mmol), catalyst (10 mol %), ligand (10 mol %), [CO] (HCOOH + Ac_2_O, 2.5 mmol), base (1.5 equiv), solvent (2 mL), 90 °C, 16 h, isolated yields.

With the optimized reaction conditions, we first explored the generality of this nickel-catalyzed 8-endo cyclization/carbonylation reaction with a variety of arylboronic acids. As shown in Scheme 2, phenylboronic acid was well-tolerated to give the final product in good yield (**3aa**). Substrates with electron-donating groups, such as methyl, ethyl, *tert*-methyl, methoxy, and *N,N*-diphenyl groups, were all compatible well to give the desired products in moderate to high yields (**3ab**-**3ah**). Those substrates with *para*- and *meta*-substituents provided the target products in higher yields than the *ortho*-substituent, probably due to the steric hindrance effect (**3ab**, **3ac** vs. **3ad**) Arylboronic acids bearing an electron-withdrawing group like an acetyl group were tested, and the expected product was obtained in 61% yield (**3ai**). Halogen groups, including fluoro, chloro, and bromo, could also react efficiently to afford the corresponding products in moderate yields (**3aj**-**3al**). Furthermore, biphenyl and 9,9-dimethyl-9*H*-fluoren-2-yl moieties proved to be suitable substrates under the standard reaction conditions, furnishing products **3am** and **3an** in 74% and 73% yields. It was noteworthy that arylboronic acids with disubstituents also exhibited good reactivity in the synthesis of eight-membered lactams (**3ao**, **3ap**). However, the reactions failed with activated boronic acids, such as strong electron-deficient substituted arylboronic acids, heterocyclicboronic acids, alkylboronic acids, and alkenylboronic acids (**3aq**-**3aw**).

**Scheme 2:**
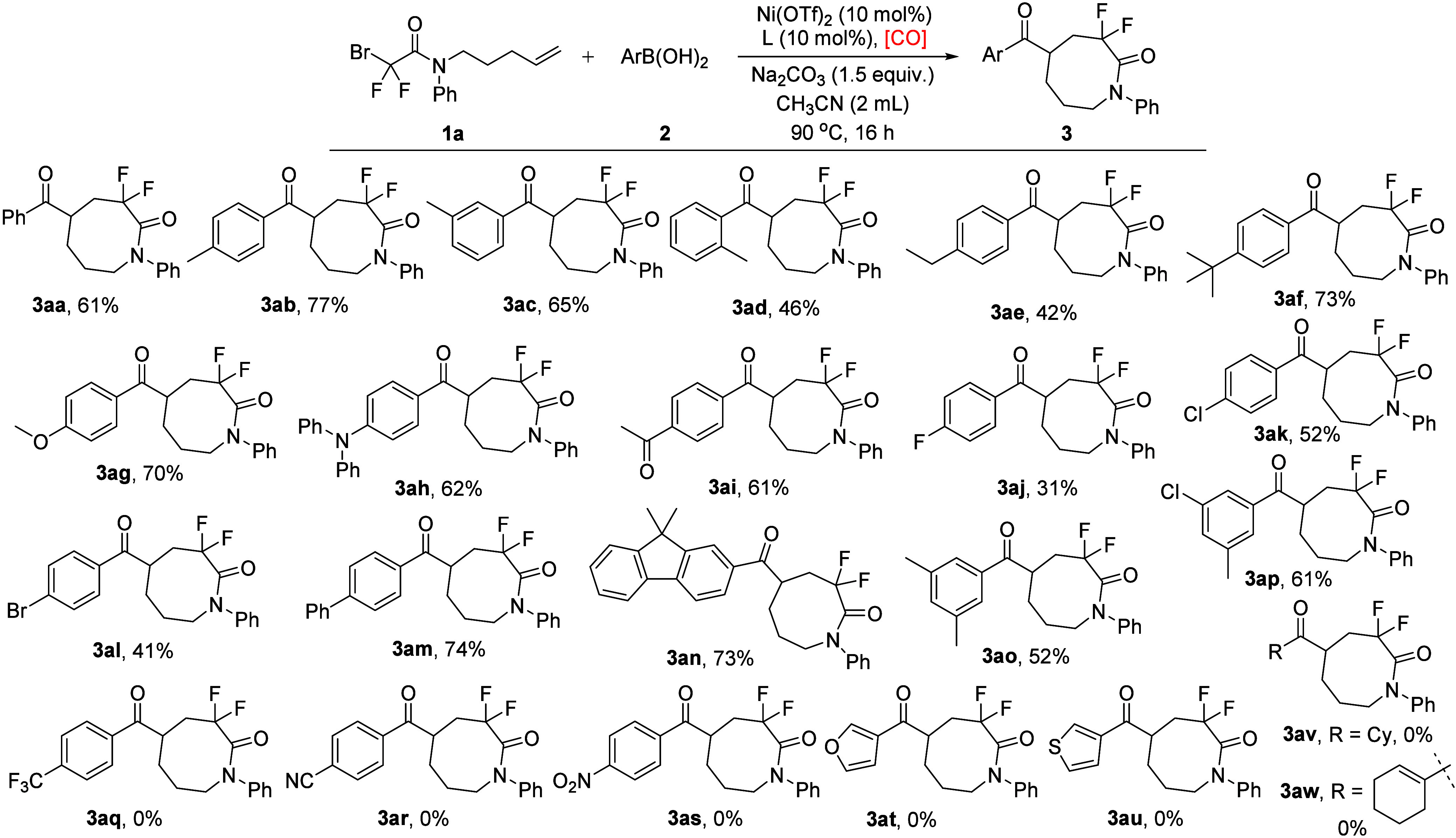
Substrate Scope of Arylboronic Acids^a^

a Reaction conditions: **1a** (0.2 mmol), **2** (0.3 mmol), Ni­(OTf)_2_ (10 mol %), L (10 mol %), [CO] (HCOOH + Ac_2_O, 2.5 mmol), Na_2_CO_3_ (1.5 equiv), CH_3_CN (2 mL), 90 °C, 16 h, isolated yields.

Next, the effect of substituents on the aniline moiety was explored under optimal reaction conditions (Scheme 3). *N*-Aryl bromodifluoroacetamides with electron-rich groups, such as methyl, *tert*-butyl, and methoxy, successfully reacted to afford the desired eight-membered lactams in moderate yields (**3bb**-**3fb**). The fluoro group was examined, and the corresponding product was obtained in 38% yield (**3gb**). Notably, when *N*-cyclopropyl bromodifluoroacetamides were used as the substrate, the expected product was produced in 81% yield (**3hb**). Additionally, we also attempted to obtain a 7-membered ring lactam but failed. It is worth mentioning that the noncarbonylation product was the main byproduct detectable in the cases of low yields.

**Scheme 3:**
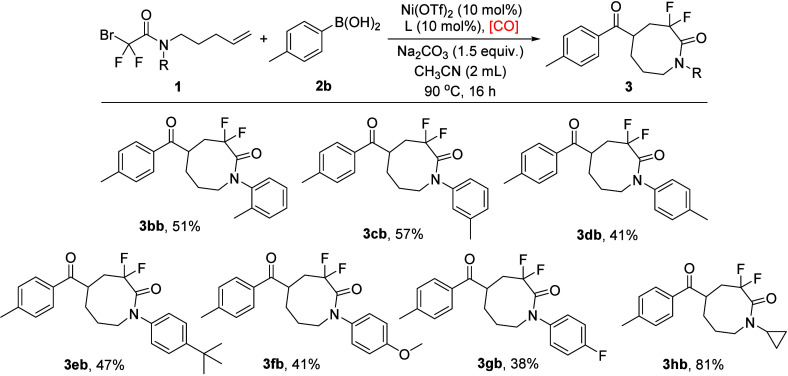
Substrate Scope of Bromodifluoroacetamides^a^

a Reaction conditions: **1** (0.2 mmol), **2b** (0.3 mmol), Ni­(OTf)_2_ (10 mol %), L (10 mol %), [CO] (HCOOH + Ac_2_O, 2.5 mmol), Na_2_CO_3_ (1.5 equiv), CH_3_CN (2 mL), 90 °C, 16 h, isolated yields.

Furthermore, to gain more insight into the reaction mechanism, a control experiment was performed in Scheme 4a. Under the standard reaction conditions, TEMPO was added to the reaction mixture as the radical trapping agent, and the desired product was not observed, which indicated that a free radical process may be included in this reaction. From the reaction mechanism point of view, the gem-difluoro group is important for the C–Br activation and cyclization steps, which are crucial for the success of this transformation.

**Scheme 4:**
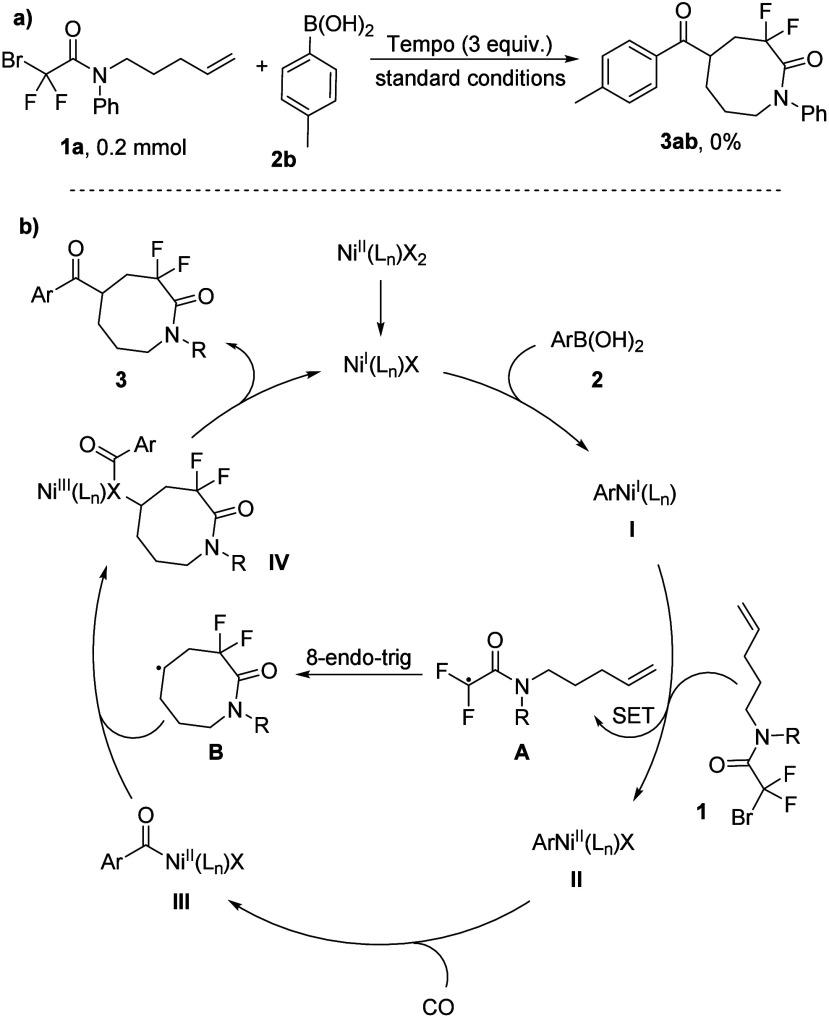
Control Experiment and Plausible Reaction Mechanism

On the basis of the above results and previous reports, a possible reaction mechanism was proposed (Scheme 4b). First, transmetalation of arylboronic acids **2** with Ni­(I) provides aryl Ni­(I) species **I**, followed by an SET process with bromodifluoroacetamides **1** to give aryl Ni­(II) complexes **II** and radical **A**. Then, a CO coordination and insertion to Ni­(II) complexes **II** afford acyl Ni­(III) intermediates **III**. At the same time, 8-*endo*-*trig* cyclization of radical **A** occurs and leads to radical **B**,^16^ which then reacts with intermediates **III** to afford complexes **IV**. Finally, reductive elimination of complexes **IV** furnishes product **3** and regenerates Ni­(I) for the next catalytic cycle.

In summary, a general and straightforward nickel-catalyzed 8-endo cyclization/carbonylation of bromodifluoroacetamides with arylboronic acids has been disclosed. With formic acid as the CO source, a variety of eight-membered lactams bearing a gem-difluoro group were obtained in moderate to high yields with high regioselectivity and good functional-group tolerance. It provides a good supplement to access eight-membered lactams with a diverse set of functional groups via a nickel-catalyzed carbonylation reaction.

## Supplementary Material



## Data Availability

The data underlying this study are available in the published article and its Supporting Information.

## Notes

General comments, general procedure, optimization details, analytic data and NMR spectra (PDF)
