# Supplementary material for: Nickel-Catalyzed 8‑Endo Cyclization/Carbonylation for the Synthesis of Eight-Membered Lactams
Source: Org Lett. 2026 Jun 29;28(27):8748–53. doi: 10.1021/acs.orglett.6c02643 (PMC13366684; doi:10.1021/acs.orglett.6c02643)

Supporting Information

Nickel-Catalyzed 8-Endo Cyclization/Carbonylation for the Synthesis of Eight-Membered Lactams

Yibin He,[†] Zilin Huang,[†] Yuting Jiang,[†] Hucheng Ma,[†] Xinxin Qi,*[,†] Xiao-Feng Wu*[,‡]

[†]School of Chemistry and Chemical Engineering, Key Laboratory of Surface & Interface Science of Polymer Materials of Zhejiang Province, Zhejiang Sci-Tech University, Hangzhou, Zhejiang 310018, People's Republic of China.

[‡]Dalian National Laboratory for Clean Energy, Dalian Institute of Chemical Physics, Chinese Academy of Sciences, 116023, Dalian, Liaoning, China; Leibniz-Institut für Katalyse e.V., Albert-Einstein-Straße 29a, Rostock 18059, Germany. E-mail: xiao-feng.wu@catalysis.de

Table of Contents

1. General Information 2

2. General Procedure 3

2.1 General Procedure for the Synthesis of Bromodifluoroacetamides 3

2.2 General Procedure for the Synthesis of Products 4

3. Characterization Data of Bromodifluoroacetamides (1a-1h) 5

4. Characterization Data of Products 10

5. Reference 24

6. Copy of ^{1}H ,^{13}C and ^{19}F NMR Spectra of Bromodifluoroacetamides (1a-1h). 25

7. Copy of ^{1}H ,^{13}C and ^{19}F NMR Spectra of Products. 41

8. Copy of DEPT-135 Spectra of 3hb. 87

1. General Information

Unless otherwise noted, all reactions were carried out under N_2 atmosphere. All the reagents were ordered from Adamas-beta®, Energy Chemical Sigma-Aldrich, Bidepharm and used without purification. All solvents were dry solvents. Column chromatography was performed on silica gel (200-300 meshes) using dichloromethane and ethyl acetate as eluent. NMR spectra were recorded on a Bruker Avance operating at for ^{1}H NMR at 400 MHz, ^{13}C NMR at 101 MHz and spectral data were reported in ppm relative to tetramethylsilane (TMS) as internal standard and $CDCl_3$ (^{1}H NMR δ 7.26, ^{13}C NMR δ 77.16) as solvent. All coupling constants (J) are reported in Hz. The following abbreviations were used to describe peak splitting patterns when appropriate: s = singlet, d = doublet, dd = double doublet, ddd = double doublet of doublets, t = triplet, dt = double triplet, q = quatriplet, m = multiplet, br = broad. Gas chromatography (GC) analyses were performed on a Shimadzu GC-2014C chromatograph equipped with a FID detector. Mass spectra (MS) were measured on spectrometer by direct inlet at 70 eV. Mass spectroscopy data of the products were collected on an HRMS-TOF instrument or Waters TOFMS GCT Premier using EI or ESI ionization. Melting points were measured with WRR digital point apparatus and not corrected.

2. General Procedure

2.1 General Procedure for the Synthesis of Bromodifluoroac etamides

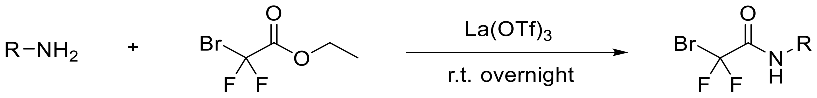

S1

Step I: Substrates S1 were prepared according to the procedures reported in the literature. To a round-bottom flask equipped with stir bar was added aryl/alkylamine (10.0 mmol, 1.0 equiv.) under argon, then ethyl bromodifluoroacetate (14 mmol, 1.4 equiv.) was added with lanthanum trifluoromethanesulfonate (5 mol %). The mixture was stirred in a 35 °C oil bath and monitored by TLC. After the amine was exhausted, the resulting mixture was quenched by H_2O and extracted with ethyl acetate, and then the extract was washed with brine and dried over $MgSO_4$. The solvent was removed in vacuo and the residue was purified by column chromatography (petroleum ether : ethyl acetate = 10 : 1) on silica gel to give the corresponding products **S1** as white solid.[1]

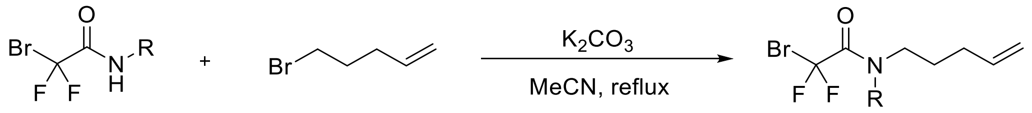

S1 **1**

Step II: Substrate 1 were prepared according to the procedures reported in the literature. To a solution of **S1** (6.0 mmol, 1.0 equiv.) in acetonitrile (0.3 M) was added K_2CO_3 (2.5 g, 18 mmol, 3 equiv.) and 5-bromo-1-pentene (2.1 mL, 18 mmol, 3 equiv.). The reaction mixture was heated (heat source was oil bath) to reflux and stirred for 24 hours. Then the solvent was evaporated under reduced pressure, quenched with H_2O, and extracted with ethyl acetate (3×10 mL). The organic layers were combined and dried over Na_2SO_4, filtered, and concentrated. The crude product was purified by silica gel column chromatography (petroleum ether : ethyl acetate = 100 : 1) to give the products **1** as yellow oil.[2]

2.2 General Procedure for the Synthesis of Products

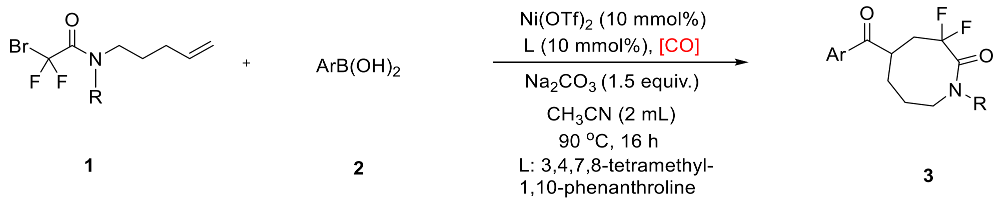

1 (0.2 mmol, 1.0 equiv.), **2** (0.3 mmol, 1.5 equiv.), $Ni(OTf)_2$ (10 mol%), 3,4,7,8-tetramethyl-1,10-phenanthroline (10 mol%), Na_2CO_3 (0.3 mmol, 1.5 equiv.) were added to an oven-dried tube (20 mL), which was then placed under vacuum and refilled with nitrogen for three times. Then dry CH_3CN (2.0 mL) was added into the tube via a syringe. A mixture of formic acid (2.5 mmol) and acetic anhydride (2.5 mmol), which was stirred at 30 °C for 1.5 h and then added to the small inner tube with Et_3N (2.5 mmol). The tube was sealed and the mixture was stirred at 90 °C (oil bath) for 16 h. After the reaction was completed, the reaction mixture was filtered and concentrated under vacuum. The crude product was purified by column chromatography (petroleum ether : ethyl acetate = 20 : 1 to 5 : 1) on silica gel to afford the corresponding product **3**.

1 mmol scale: **1a** (1 mmol, 1.0 equiv.), **2b** (1.5 mmol, 1.5 equiv.), $Ni(OTf)_2$ (10 mol%, 0.1 mmol, 35.7 mg), 3,4,7,8-tetramethyl-1,10-phenanthroline (10 mol%, 0.1 mmol, 23.6 mg), Na_2CO_3 (1.5 mmol, 1.5 equiv., 159 mg) were added to an oven-dried tube (50 mL), which was then placed under vacuum and refilled with nitrogen for three times. Then dry CH_3CN (10.0 mL) was added into the tube via a syringe. A mixture of formic acid (12.5 mmol) and acetic anhydride (12.5 mmol), which was stirred at 30 °C for 1.5 h and then added to the small inner tube with Et_3N (12.5 mmol). The tube was sealed and the mixture was stirred at 90 °C (oil bath) for 16 h. After the reaction was completed, the reaction mixture was filtered and concentrated under vacuum. The crude product was purified by column chromatography (petroleum ether : ethyl acetate = 20 : 1 to 5 : 1) on silica gel to afford the corresponding product **3ab** in 65% yield (232.1 mg).

3. Characterization Data of Bromodifluoroacetamides (1a-1h)

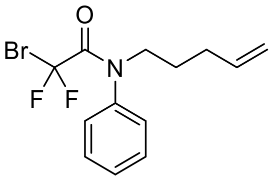

2-bromo-2,2-difluoro-*N*-(pent-4-en-1-yl)-*N*-phenylacetamide (1a)

Upon completion the mixture was concentrated and purified via flash column chromatography (petroleum ether : ethyl acetate = 100 : 1) to give the titled product **1a** as a **yellow oil** with 95% purity (1.62g, 85%).

^{1}H NMR (400 MHz, CDCl$_3$) δ 7.48 – 7.43 (m, 3H), 7.30 (d, J = 6.0 Hz, 2H), 5.85 – 5.75 (m, 1H), 5.06 – 4.99 (m, 2H), 3.78 – 3.74 (m, 2H), 2.15 – 2.09 (m, 2H), 1.77 – 1.70 (m, 2H).

^{13}C NMR (101 MHz, CDCl$_3$) δ 158.92 (t, J = 25.7 Hz), 139.7, 137.3, 129.3, 129.0, 128.7, 115.5, 111.64 (t, J = 317.6 Hz), 52.4, 30.8, 26.0.

^{19}F NMR (376 MHz, CDCl$_3$) δ -51.77.

HRMS (ESI-TOF) m/z: [M+H]$^+$ Calcd. for $C_{13}H_{15}BrF_2NO$ 318.0300; found: 318.0304.

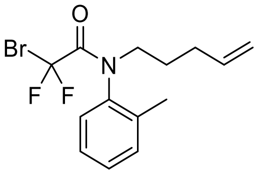

2-bromo-2,2-difluoro-*N*-(pent-4-en-1-yl)-*N*-(*o*-tolyl)acetamide (1b)

Upon completion the mixture was concentrated and purified via flash column chromatography (petroleum ether : ethyl acetate = 100 : 1) to give the titled product **1b** as a **yellow oil** with 95% purity (1.39g, 70%).

^{1}H NMR (400 MHz, CDCl$_3$) δ 7.30 – 7.27 (m, 2H), 7.22 (t, J = 3.2 Hz, 2H), 5.81 – 5.71 (m, 1H), 5.03 – 4.94 (m, 2H), 4.24 – 4.17 (m, 1H), 3.06 – 2.99 (m, 1H), 2.24 (s, 3H), 2.11 – 2.06 (m, 2H), 1.85 – 1.76 (m, 1H), 1.71 – 1.61 (m, 1H).

^{13}C NMR (101 MHz, CDCl$_3$) δ 159.00 (t, J = 25.6 Hz), 139.0, 137.3, 137.0, 129.9, 128.4, 120.5, 115.4, 113.0, 111.64 (t, J = 317.8 Hz), 52.4, 30.7, 26.0, 21.2.

^{19}F NMR (376 MHz, CDCl$_3$) δ -51.71.

HRMS (ESI-TOF) m/z: $[M+H]^+$ Calcd. for $C_{14}H_{17}BrF_2NO$ 332.0456; found: 332.0461.

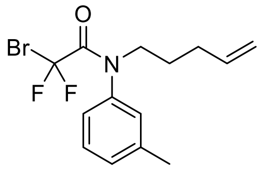

2-bromo-2,2-difluoro-*N*-(pent-4-en-1-yl)-*N*-(*m*-tolyl)acetamide (1c)

Upon completion the mixture was concentrated and purified via flash column chromatography (petroleum ether : ethyl acetate = 100 : 1) to give the titled product **1c** as a **yellow oil** with 95% purity (1.51g, 76%).

^{1}H NMR (400 MHz, $CDCl_3$) δ 7.30 (t, J = 8.1 Hz, 1H), 7.20 (d, J = 7.7 Hz, 1H), 7.06 (d, J = 7.2 Hz, 2H), 5.82 – 5.72 (m, 1H), 5.04 – 4.94 (m, 2H), 3.71 (t, J = 7.8 Hz, 2H), 2.38 (s, 3H), 2.11 – 2.05 (m, 2H), 1.74 – 1.66 (m, 2H).

^{13}C NMR (101 MHz, $CDCl_3$) δ 158.90 (t, J = 25.5 Hz), 139.6, 139.4, 137.3, 129.7, 129.0, 125.6, 115.4, 111.72 (t, J = 317.7 Hz), 52.4, 30.8, 26.0, 21.3.

^{19}F NMR (376 MHz, $CDCl_3$) δ -51.73.

HRMS (ESI-TOF) m/z: $[M+H]^+$ Calcd. for $C_{14}H_{17}BrF_2NO$ 332.0456; found: 332.0461.

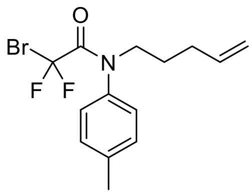

2-bromo-2,2-difluoro-*N*-(pent-4-en-1-yl)-*N*-(*p*-tolyl)acetamide (1d)

Upon completion the mixture was concentrated and purified via flash column chromatography (petroleum ether : ethyl acetate = 100 : 1) to give the titled product **1d** as a **yellow oil** with 95% purity (1.59g, 80%).

^{1}H NMR (400 MHz, $CDCl_3$) δ 7.22 (d, J = 8.1 Hz, 2H), 7.14 (d, J = 8.1 Hz, 2H), 5.82 – 5.71 (m, 1H), 5.03 – 4.95 (m, 2H), 3.72 – 3.68 (m, 2H), 2.39 (s, 3H), 2.08 (q, J = 6.8, 6.4 Hz, 2H), 1.73 – 1.65 (m, 2H).

^{13}C NMR (101 MHz, $CDCl_3$) δ 159.03 (t, J = 25.4 Hz), 139.0, 137.3, 137.0, 129.9, 129.3, 128.4, 115.4, 111.66 (t, J = 317.5 Hz), 52.4, 30.8, 26.0, 21.2.

^{19}F NMR (376 MHz, $CDCl_3$) δ -51.72.

HRMS (ESI-TOF) m/z: $[M+H]^+$ Calcd. for $C_{14}H_{17}BrF_2NO$ 332.0456; found: 332.0461.

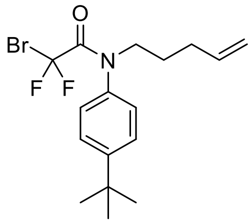

2-bromo-*N*-(4-(*tert*-butyl)phenyl)-2,2-difluoro-*N*-(pent-4-en-1-yl)acetamide (1e)

Upon completion the mixture was concentrated and purified via flash column chromatography (petroleum ether : ethyl acetate = 100 : 1) to give the titled product **1e** as a **yellow oil** with 95% purity (1.39mg, 62%).

^{1}H NMR (400 MHz, CDCl$_3$) δ 7.41 (d, J = 8.6 Hz, 2H), 7.24 – 7.16 (m, 2H), 5.85 – 5.73 (m, 1H), 5.04 – 4.96 (m, 2H), 3.73 – 3.69 (m, 2H), 2.12 – 2.06 (m, 2H), 1.73 – 1.69 (m, 2H), 1.34 (s, 9H).

^{13}C NMR (101 MHz, CDCl$_3$) δ 159.01 (t, J = 25.5 Hz), 152.0, 137.3, 137.0, 128.1, 126.2, 115.4, 112.5, 111.83 (t, J = 329.9 Hz), 52.4, 34.8, 31.3, 30.8, 26.0.

^{19}F NMR (376 MHz, CDCl$_3$) δ -51.67.

HRMS (ESI-TOF) m/z: $[M+Na]^+$ Calcd. for $C_{17}H_{22}BrF_2NNaO$ 396.0745; found: 396.0752.

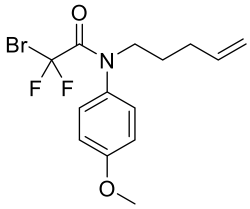

2-bromo-2,2-difluoro-*N*-(4-methoxyphenyl)-*N*-(pent-4-en-1-yl)acetamide (1f)

Upon completion the mixture was concentrated and purified via flash column chromatography (petroleum ether : ethyl acetate = 100 : 1) to give the titled product **1f** as a **yellow oil** with 95% purity (1.21g, 58%).

^{1}H NMR (400 MHz, CDCl$_3$) δ 7.16 (d, J = 8.9 Hz, 2H), 6.89 (d, J = 8.9 Hz, 2H), 5.78 – 5.68 (m, 1H), 5.00 – 4.91 (m, 2H), 3.79 (s, 3H), 3.66 (t, 2H), 2.07 – 2.02 (m, 2H), 1.69 – 1.62 (m, 2H).

^{13}C NMR (101 MHz, CDCl$_3$) δ 159.7, 159.07 (t, J = 25.4 Hz), 137.3, 132.1, 129.9, 115.4, 114.3, 111.62 (t, J = 317.7 Hz), 55.5, 52.4, 30.7, 25.6.

^{19}F NMR (376 MHz, CDCl$_3$) δ -52.23.

HRMS (ESI-TOF) m/z: $[M+H]^+$ Calcd. for $C_{14}H_{17}BrF_2NO_2$ 348.0405; found: 348.0410.

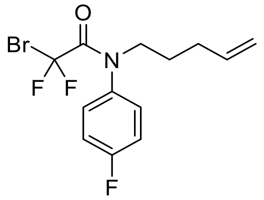

2-bromo-2,2-difluoro-*N*-(4-fluorophenyl)-*N*-(pent-4-en-1-yl)acetamide (1g)

Upon completion the mixture was concentrated and purified via flash column chromatography (petroleum ether : ethyl acetate = 100 : 1) to give the titled product **1g** as a **yellow oil** with 95% purity (1.28g, 60%).

^{1}H NMR (400 MHz, CDCl$_3$) δ 7.29 (dd, J = 9.0, 4.7 Hz, 2H), 7.14 (t, J = 8.5 Hz, 2H), 5.84 – 5.74 (m, 1H), 5.06 – 4.99 (m, 2H), 3.75 – 3.72 (m, 2H), 2.14 – 2.09 (m, 2H), 1.75 – 1.68 (m, 2H).

^{13}C NMR (101 MHz, CDCl$_3$) δ 163.7, 161.2, 158.91 (t, J = 25.6 Hz), 137.1, 135.6, 130.61 (d, J = 8.9 Hz), 116.5, 116.2, 115.6, 111.45 (t, J = 317.4 Hz), 52.4, 30.7, 25.9.

^{19}F NMR (376 MHz, CDCl$_3$) δ -51.92, -111.62.

HRMS (ESI-TOF) m/z: $[M+H]^+$ Calcd. for $C_{13}H_{14}BrF_3NO$ 336.0205; found: 336.0210

HRMS (ESI-TOF) m/z: $[M+Na]^+$ Calcd. for $C_{14}H_{16}BrF_2NO$ 354.0267; found: 354.0281.

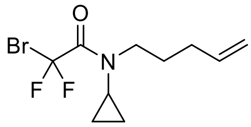

2-bromo-*N*-cyclopropyl-2,2-difluoro-*N*-(pent-4-en-1-yl)acetamide (1h)

Upon completion the mixture was concentrated and purified via flash column chromatography (petroleum ether : ethyl acetate = 100 : 1) to give the titled product **1h** as a **colorless oil** with 95% purity (1.11g, 66%).

^{1}H NMR (400 MHz, CDCl$_3$) δ 5.84 – 5.73 (m, 1H), 5.06 – 4.97 (m, 2H), 3.42 (t, J = 7.6 Hz, 3H), 2.96 – 2.69 (m, 1H), 2.07 (d, J = 7.2 Hz, 2H), 1.81 – 1.65 (m, 2H), 0.93 (d, J = 6.1 Hz, 4H).

^{13}C NMR (101 MHz, CDCl$_3$) δ 161.4 (t, J = 30.5 Hz), 137.4, 115.4, 112.5 (t, J = 333.3 Hz), 48.1, 30.9, 30.5, 26.6, 9.0, 7.5.

^{19}F NMR (376 MHz, CDCl$_3$) δ -52.79, -54.15.

HRMS (ESI-TOF) m/z: $[M+H]^+$ Calcd. for $C_{10}H_{15}BrF_2NO$ 282.0300; found: 282.0302.

4. Characterization Data of Products

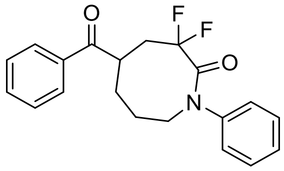

5-benzoyl-3,3-difluoro-1-phenylazocan-2-one (3aa)

Upon completion the mixture was concentrated and purified via flash column chromatography (petroleum ether : ethyl acetate = 5 : 1) to give the titled product **3aa** as a **white solid** (41.9 mg, 61%, 95% purity).

^{1}H NMR (400 MHz, CDCl$_3$) δ 7.96 (d, J = 7.7 Hz, 2H), 7.63 (t, J = 7.3 Hz, 1H), 7.52 (t, J = 7.6 Hz, 2H), 7.45 (t, J = 7.6 Hz, 2H), 7.35 (t, J = 7.4 Hz, 1H), 7.24 (d, J = 7.9 Hz, 2H), 4.67 – 4.58 (m, 1H), 3.70 (d, J = 16.3 Hz, 1H), 3.63 – 3.58 (m, 1H), 2.66 – 2.53 (m, 2H), 2.19 (d, J = 14.9 Hz, 1H), 1.97 – 1.85 (m, 1H), 1.79 – 1.67 (m, 2H).

^{13}C NMR (101 MHz, CDCl$_3$) δ 200.0, 164.46 (t, J = 20.2 Hz), 141.7, 134.9, 133.7, 129.7, 129.1, 128.4, 128.0, 127.1, 118.59 (t, J = 252.5 Hz), 47.56 (d, J = 13.9 Hz), 40.72 (dd, J = 29.1, 23.2 Hz), 38.77 (d, J = 9.8 Hz), 27.1, 27.0.

^{19}F NMR (376 MHz, CDCl$_3$) δ -88.06 (d, J = 253.6 Hz), -99.40 (d, J = 253.0 Hz).

HRMS (ESI-TOF) m/z: $[M+H]^+$ Calcd. for $C_{20}H_{20}F_2NO_2$ 344.1457; found: 344.1459.

M.p. 143.2-144.5 °C

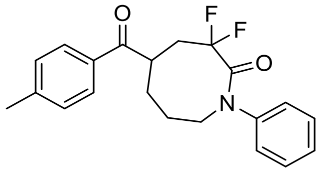

3,3-difluoro-5-(4-methylbenzoyl)-1-phenylazocan-2-one (3ab)

Upon completion the mixture was concentrated and purified via flash column chromatography (petroleum ether : ethyl acetate = 5 : 1) to give the titled product **3ab** as a **colorless oil** (55.3 mg, 77%, 95% purity).

^{1}H NMR (400 MHz, CDCl$_3$) δ 7.86 (d, J = 8.3 Hz, 2H), 7.45 (t, J = 7.8 Hz, 2H), 7.34 (d, J = 7.8 Hz, 3H), 7.23 (d, J = 7.2 Hz, 2H), 4.67 – 4.56 (m, 1H), 3.70 (d, J = 15.5 Hz,

1H), 3.60 – 3.55 (m, 1H), 2.67 – 2.50 (m, 2H), 2.44 (s, 3H), 2.18 (d, J = 14.6 Hz, 1H), 1.94 – 1.86 (m, 1H), 1.76 – 1.61 (m, 2H).

^{13}C NMR (101 MHz, CDCl$_3$) δ 199.7, 164.53 (t, J = 30.3 Hz), 144.7, 141.7, 132.3, 130.3, 129.8, 129.7, 129.3, 128.6, 128.0, 127.1, 118.66 (t, J = 242.4 Hz), 47.61 (d, J = 14.0 Hz), 40.79 (dd, J = 28.9, 22.9 Hz), 38.66 (d, J = 9.8 Hz), 27.2, 27.0, 21.8.

^{19}F NMR (376 MHz, CDCl$_3$) δ -88.06 (d, J = 252.4 Hz), -99.39 (d, J = 255.2 Hz).

HRMS (ESI-TOF) m/z: $[M+H]^+$ Calcd. for $C_{21}H_{22}F_2NO_2$ 358.1613; found: 358.1616.

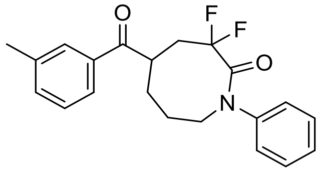

3,3-difluoro-5-(3-methylbenzoyl)-1-phenylazocan-2-one (3ac)

Upon completion the mixture was concentrated and purified via flash column chromatography (petroleum ether : ethyl acetate = 5 : 1) to give the titled product **3ac** as a **colorless oil** (46.5 mg, 65%, 95% purity).

^{1}H NMR (400 MHz, CDCl$_3$)δ 7.77 (s, 1H), 7.74 (d, J = 7.1 Hz, 1H), 7.47 – 7.40 (m, 4H), 7.38 – 7.32 (m, 1H), 7.24 (d, J = 7.3 Hz, 2H), 4.67 – 4.58 (m, 1H), 3.70 (d, J = 15.7 Hz, 1H), 3.61 – 3.56 (m, 1H), 2.65 – 2.53 (m, 2H), 2.44 (s, 3H), 2.18 (d, J = 15.2 Hz, 1H), 1.94 – 1.87 (m, 1H), 1.79 – 1.65 (m, 2H).

^{13}C NMR (101 MHz, CDCl$_3$) δ 200.2, 164.49 (t, J = 60.6 Hz), 141.7, 139.0, 135.0, 134.5, 129.7, 129.1, 128.9, 128.0, 127.1, 125.5, 118.59 (t, J = 499.8 Hz), 47.58 (d, J = 14.1 Hz), 40.75 (dd, J = 28.7, 23.1 Hz), 38.80 (d, J = 9.8 Hz), 27.1, 27.0, 21.5.

^{19}F NMR (376 MHz, CDCl$_3$) δ -88.05 (d, J = 251.5 Hz), -99.41 (d, J = 251.7 Hz).

HRMS (ESI-TOF) m/z: $[M+H]^+$ Calcd. for $C_{21}H_{22}F_2NO_2$ 358.1613; found: 358.1616.

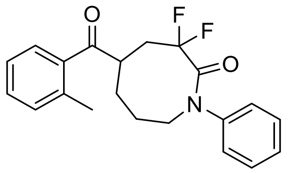

3,3-difluoro-5-(2-methylbenzoyl)-1-phenylazocan-2-one (3ad)

Upon completion the mixture was concentrated and purified via flash column chromatography (petroleum ether : ethyl acetate = 5 : 1) to give the titled product **3ad**

as a **yellow oil** (32.9 mg, 46%, 95% purity).

^{1}H NMR (400 MHz, CDCl$_3$) δ 7.57 (d, J = 7.4 Hz, 1H), 7.43 (q, J = 7.7 Hz, 3H), 7.36 – 7.26 (m, 3H), 7.21 (d, J = 7.2 Hz, 2H), 4.61 – 4.48 (m, 1H), 3.64 (d, J = 16.0 Hz, 1H), 3.49 – 3.40 (m, 1H), 2.74 – 2.49 (m, 2H), 2.48 (s, 3H), 2.16 (d, J = 14.5 Hz, 1H), 1.90 – 1.79 (m, 1H), 1.76 – 1.65 (m, 2H).

^{13}C NMR (101 MHz, CDCl$_3$) δ 203.6, 164.50 (dd, J = 29.1, 25.9 Hz), 141.7, 139.1, 136.4, 132.3, 131.7, 129.7, 129.5, 127.9, 127.5, 127.1, 126.0, 118.59 (dd, J = 253.2, 244.8 Hz), 47.53 (d, J = 14.1 Hz), 41.51 (d, J = 9.6 Hz), 40.37 (dd, J = 29.0, 23.2 Hz), 27.2, 26.4, 21.1.

^{19}F NMR (376 MHz, CDCl$_3$) δ -88.15 (d, J = 252.7 Hz), -99.44 (d, J = 253.7 Hz).

HRMS (ESI-TOF) m/z: $[M+H]^+$ Calcd. for $C_{21}H_{22}F_2NO_2$ 358.1613; found: 358.1616.

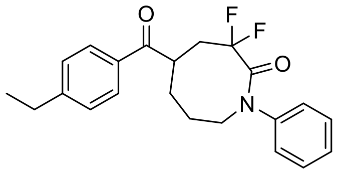

5-(4-ethylbenzoyl)-3,3-difluoro-1-phenylazocan-2-one (3ae)

Upon completion the mixture was concentrated and purified via flash column chromatography (petroleum ether : ethyl acetate = 5 : 1) to give the titled product **3ae** as a **colorless oil** (31.2 mg, 42%, 95% purity).

^{1}H NMR (400 MHz, CDCl$_3$) δ 7.89 (d, J = 8.1 Hz, 2H), 7.45 (t, J = 7.7 Hz, 2H), 7.35 (t, J = 7.5 Hz, 3H), 7.24 (d, J = 7.5 Hz, 2H), 4.65 – 4.57 (m, 1H), 3.70 (d, J = 16.6 Hz, 1H), 3.61 – 3.56 (m, 1H), 2.73 (q, J = 7.6 Hz, 2H), 2.65 – 2.49 (m, 2H), 2.19 (d, J = 15.4 Hz, 1H), 1.94 – 1.88 (m, 1H), 1.78 – 1.66 (m, 2H), 1.28 (t, J = 7.7 Hz, 3H).

^{13}C NMR (101 MHz, CDCl$_3$) δ 199.7, 164.46 (dd, J = 29.2, 26.0 Hz), 150.8, 141.7, 132.5, 129.6, 128.6, 128.6, 127.9, 127.0, 126.0, 118.63 (dd, J = 254.1, 245.3 Hz), 47.55 (d, J = 14.1 Hz), 40.75 (dd, J = 28.8, 23.0 Hz), 38.60 (d, J = 9.7 Hz), 29.0, 27.2, 27.0, 15.2.

^{19}F NMR (376 MHz, CDCl$_3$) δ -88.10 (d, J = 253.6 Hz), -99.39 (d, J = 253.6 Hz).

HRMS (ESI-TOF) m/z: $[M+H]^+$ Calcd. for $C_{22}H_{24}F_2NO_2$ 372.1770; found: 372.1772.

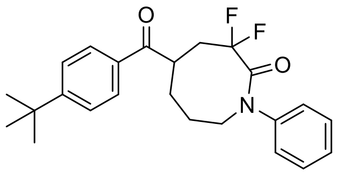

5-(4-(*tert*-butyl)benzoyl)-3,3-difluoro-1-phenylazocan-2-one (3af)

Upon completion the mixture was concentrated and purified via flash column chromatography (petroleum ether : ethyl acetate = 5 : 1) to give the titled product **3af** as a **yellow oil** (58.3 mg, 73%, 95% purity).

^{1}H NMR (400 MHz, CDCl$_3$) δ 7.91 (d, J = 8.5 Hz, 2H), 7.53 (d, J = 8.5 Hz, 2H), 7.45 (t, J = 7.7 Hz, 2H), 7.35 (t, J = 7.4 Hz, 1H), 7.24 (d, J = 7.2 Hz, 2H), 4.65 – 4.57 (m, 1H), 3.71 (d, J = 15.8 Hz, 1H), 3.62 – 3.57 (m, 1H), 2.65 – 2.50 (m, 2H), 2.21 (d, J = 14.8 Hz, 1H), 1.95 – 1.88 (m, 1H), 1.80 – 1.69 (m, 2H), 1.36 (s, 9H).

^{13}C NMR (101 MHz, CDCl$_3$) δ 199.7, 164.52 (dd, J = 28.9, 26.1 Hz), 157.6, 141.7, 132.2, 130.1, 129.7, 128.5, 128.0, 127.1, 126.1, 125.5, 118.65 (dd, J = 253.2, 244.7 Hz), 47.64 (d, J = 14.0 Hz), 40.82 (dd, J = 28.3, 23.4 Hz), 38.68 (d, J = 9.6 Hz), 35.27, 31.1, 27.1, 27.1.

^{19}F NMR (376 MHz, CDCl$_3$) δ -88.11 (d, J = 253.5 Hz), -99.37 (d, J = 255.2 Hz).

HRMS (ESI-TOF) m/z: $[M+H]^+$ Calcd. for $C_{24}H_{28}F_2NO_2$ 400.2083; found: 400.2086.

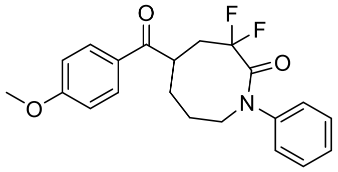

3,3-difluoro-5-(4-methoxybenzoyl)-1-phenylazocan-2-one (3ag)

Upon completion the mixture was concentrated and purified via flash column chromatography (petroleum ether : ethyl acetate = 5 : 1) to give the titled product **3ag** as a **yellow oil** (52.3 mg, 70%, 95% purity).

^{1}H NMR (400 MHz, CDCl$_3$) δ 7.94 (d, J = 8.9 Hz, 2H), 7.44 (t, J = 7.7 Hz, 2H), 7.34 (t, J = 7.5 Hz, 1H), 7.23 (d, J = 7.3 Hz, 2H), 6.99 (d, J = 8.9 Hz, 2H), 4.65 – 4.56 (m, 1H), 3.89 (s, 3H), 3.69 (d, J = 16.9 Hz, 1H), 3.58 – 3.53 (m, 1H), 2.64 – 2.53 (m, 2H), 2.18 (d, J = 13.6 Hz, 1H), 1.95 – 1.86 (m, 1H), 1.77 – 1.65 (m, 2H).

^{13}C NMR (101 MHz, CDCl$_3$) δ 198.7, 164.50 (t, J = 30.3 Hz), 164.0, 141.7, 130.8,

129.7, 127.9, 127.7, 127.1, 118.66 (t, J = 242.4 Hz), 114.3, 55.6, 47.61 (d, J = 13.9 Hz), 40.94 (dd, J = 28.9, 23.0 Hz), 38.42 (d, J = 9.7 Hz), 27.3, 27.0.

^{19}F NMR (376 MHz, CDCl$_3$) δ -87.98 (d, J = 252.9 Hz), -99.33 (d, J = 250.8 Hz).

HRMS (ESI-TOF) m/z: [M+H]$^+$ Calcd. for $C_{21}H_{22}F_2NO_3$ 374.1562; found: 374.1565.

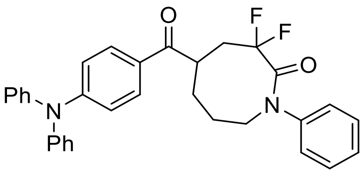

5-(4-(diphenylamino)benzoyl)-3,3-difluoro-1-phenylazocan-2-one (3ah)

Upon completion the mixture was concentrated and purified via flash column chromatography (petroleum ether : ethyl acetate = 5 : 1) to give the titled product **3ah** as a **yellow oil** (63.3 mg, 62%, 95% purity).

^{1}H NMR (400 MHz, CDCl$_3$) δ 7.80 (d, J = 8.9 Hz, 2H), 7.45 (t, J = 7.7 Hz, 2H), 7.36 – 7.32 (m, 5H), 7.23 (d, J = 7.8 Hz, 2H), 7.18 (d, J = 7.2 Hz, 6H), 7.01 (d, J = 8.9 Hz, 2H), 4.60 – 4.53 (m, 1H), 3.68 (d, J = 11.1 Hz, 1H), 3.55 – 3.49 (m, 1H), 2.64 – 2.53 (m, 2H), 2.22 (d, J = 16.6 Hz, 1H), 1.94 – 1.87 (m, 1H), 1.67 – 1.60 (m, 2H).

^{13}C NMR (101 MHz, CDCl$_3$) δ 198.2, 164.24 (t, J = 31.9 Hz), 152.7, 146.2, 141.7, 130.0, 129.7, 129.6, 127.9, 127.1, 126.6, 126.3, 125.1, 119.3, 118.80 (t, J = 231.8 Hz), 47.62 (d, J = 14.0 Hz), 41.04 (dd, J = 28.1, 23.2 Hz), 38.22 (d, J = 9.5 Hz), 27.3, 27.1.

^{19}F NMR (376 MHz, CDCl$_3$) δ -88.10 (d, J = 253.1 Hz), -99.22 (d, J = 219.9 Hz).

HRMS (ESI-TOF) m/z: [M+H]$^+$ Calcd. for $C_{32}H_{29}F_2N_2O_2$ 511.2192; found: 511.2199.

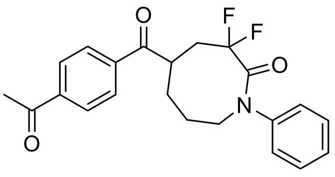

5-(4-acetylbenzoyl)-3,3-difluoro-1-phenylazocan-2-one (3ai)

Upon completion the mixture was concentrated and purified via flash column chromatography (petroleum ether : ethyl acetate = 5 : 1) to give the titled product **3ai** as a **yellow oil** (47.3 mg, 61%, 95% purity).

^{1}H NMR (400 MHz, CDCl$_3$) δ 8.08 (d, J = 8.5 Hz, 2H), 8.03 (d, J = 8.5 Hz, 2H), 7.45

(t, *J* = 7.7 Hz, 2H), 7.35 (t, *J* = 7.5 Hz, 1H), 7.23 (d, *J* = 7.1 Hz, 2H), 4.65 – 4.58 (m, 1H), 3.71 (d, *J* = 15.6 Hz, 1H), 3.62 – 3.57 (m, 1H), 2.66 (s, 3H), 2.63 – 2.49 (m, 2H), 2.16 (d, *J* = 14.9 Hz, 1H), 1.96 – 1.89 (m, 1H), 1.71 – 1.62 (m, 2H).

^{13}C NMR (101 MHz, $CDCl_3$) δ 199.3, 197.3, 164.30 (dd, *J* = 29.3, 25.9 Hz), 141.6, 140.6, 138.1, 129.6, 129.5, 128.8, 128.6, 128.4, 128.0, 127.5, 127.0, 125.9, 118.41 (dd, *J* = 253.2, 244.8 Hz), 47.44 (d, *J* = 14.1 Hz), 40.44 (dd, *J* = 29.2, 23.1 Hz), 39.17 (d, *J* = 9.8 Hz), 26.9.

^{19}F NMR (376 MHz, $CDCl_3$) δ -88.11 (d, *J* = 253.8 Hz), -99.37 (d, *J* = 253.8 Hz).

HRMS (ESI-TOF) m/z: $[M+H]^+$ Calcd. for $C_{22}H_{22}F_2NO_3$ 386.1562; found: 386.1565.

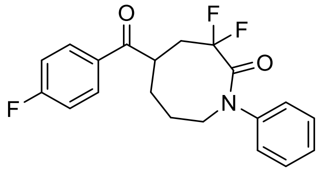

3,3-difluoro-5-(4-fluorobenzoyl)-1-phenylazocan-2-one (3aj)

Upon completion the mixture was concentrated and purified via flash column chromatography (petroleum ether : ethyl acetate = 5 : 1) to give the titled product **3aj** as a **yellow oil** (22.4 mg, 31%, 95% purity).

^{1}H NMR (400 MHz, $CDCl_3$) δ 7.99 (dd, *J* = 8.9, 5.3 Hz, 2H), 7.45 (t, *J* = 7.7 Hz, 2H), 7.35 (t, *J* = 7.4 Hz, 1H), 7.24 – 7.17 (m, 4H), 4.65 – 4.56 (m, 1H), 3.70 (d, *J* = 13.0 Hz, 1H), 3.58 – 3.53 (m, 1H), 2.64 – 2.53 (m, 2H), 2.17 (d, *J* = 14.7 Hz, 1H), 1.96 – 1.88 (m, 1H), 1.80 – 1.62 (m, 2H).

^{13}C NMR (101 MHz, $CDCl_3$) δ 198.4, 167.4, 164.43 (dd, *J* = 43.0, 39.6 Hz), 141.6, 131.12 (d, *J* = 9.3 Hz), 129.7, 128.0, 127.0, 126.0, 118.49 (dd, *J* = 255.2, 246.1 Hz), 116.4, 116.2, 47.52 (d, *J* = 14.1 Hz), 40.72 (dd, *J* = 29.1, 23.1 Hz), 38.77 (d, *J* = 9.8 Hz), 27.1, 27.0.

^{19}F NMR (376 MHz, $CDCl_3$) δ -88.07 (d, *J* = 253.7 Hz), -99.38 (d, *J* = 253.0 Hz), -103.97.

HRMS (ESI-TOF) m/z: $[M+H]^+$ Calcd. for $C_{20}H_{19}F_3NO_2$ 362.1362; found: 362.1364.

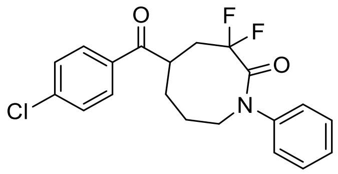

5-(4-chlorobenzoyl)-3,3-difluoro-1-phenylazocan-2-one (3ak)

Upon completion the mixture was concentrated and purified via flash column chromatography (petroleum ether : ethyl acetate = 5 : 1) to give the titled product **3ak** as a **colorless oil** (39.3 mg, 52%, 95% purity).

^{1}H NMR (400 MHz, CDCl$_3$) δ 7.90 (d, J = 8.4 Hz, 2H), 7.50 (d, J = 8.4 Hz, 2H), 7.44 (d, J = 7.6 Hz, 2H), 7.36 (d, J = 7.4 Hz, 1H), 7.23 (d, J = 7.5 Hz, 2H), 4.64 – 4.56 (m, 1H), 3.70 (d, J = 15.3 Hz, 1H), 3.56 – 3.51 (m, 1H), 2.64 – 2.53 (m, 2H), 2.16 (d, J = 14.3 Hz, 1H), 1.96 – 1.89 (m, 1H), 1.75 – 1.61 (m, 2H).

^{13}C NMR (101 MHz, CDCl$_3$) δ 198.7, 164.36 (dd, J = 28.9, 26.7 Hz), 141.6, 140.3, 133.2, 129.8, 129.7, 129.4, 129.2, 128.0, 127.0, 126.0, 118.46 (dd, J = 253.3, 245.0 Hz), 47.49 (d, J = 14.2 Hz), 40.63 (dd, J = 29.0, 23.1 Hz), 38.83 (d, J = 9.9 Hz), 27.0, 27.0.

^{19}F NMR (376 MHz, CDCl$_3$) δ -88.09 (d, J = 253.9 Hz), -99.39 (d, J = 255.6 Hz).

HRMS (ESI-TOF) m/z: $[M+H]^+$ Calcd. for $C_{20}H_{19}ClF_2NO_2$ 378.1067; found: 378.1069.

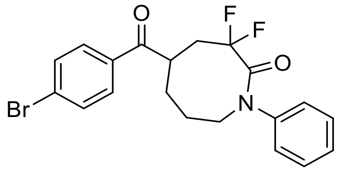

5-(4-bromobenzoyl)-3,3-difluoro-1-phenylazocan-2-one (3al)

Upon completion the mixture was concentrated and purified via flash column chromatography (petroleum ether : ethyl acetate = 5 : 1) to give the titled product **3al** as a **yellow oil** (34.7 mg, 41%, 95% purity).

^{1}H NMR (400 MHz, CDCl$_3$) δ 7.82 (d, J = 8.6 Hz, 2H), 7.66 (d, J = 8.6 Hz, 2H), 7.45 (t, J = 7.7 Hz, 2H), 7.35 (t, J = 7.4 Hz, 1H), 7.23 (d, J = 7.2 Hz, 2H), 4.65 – 4.56 (m, 1H), 3.70 (d, J = 15.2 Hz, 1H), 3.55 – 3.50 (m, 1H), 2.64 – 2.52 (m, 2H), 2.15 (d, J = 14.5 Hz, 1H), 1.92 – 1.87 (m, 1H), 1.77 – 1.65 (m, 2H).

^{13}C NMR (101 MHz, CDCl$_3$) δ 197.9, 163.26 (dd, J = 28.9, 26.4 Hz), 140.6, 132.5,

131.3, 131.0, 128.8, 128.6, 128.4, 128.4, 127.9, 126.9, 125.9, 124.9, 117.36 (dd, *J* = 253.1, 244.9 Hz), 46.39 (d, *J* = 14.1 Hz), 39.50 (dd, *J* = 29.1, 23.1 Hz), 37.72 (d, *J* = 9.8 Hz), 25.9, 25.9.

^{19}F NMR (376 MHz, CDCl$_3$) δ -88.08 (d, *J* = 250.9 Hz), -99.39 (d, *J* = 255.3 Hz).

HRMS (ESI-TOF) m/z: $[M+H]^+$ Calcd. for $C_{20}H_{19}BrF_2NO_2$ 422.0562; found: 422.0564.

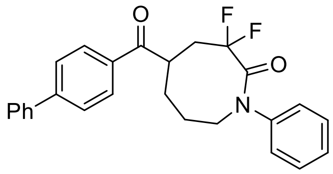

5-([1,1'-biphenyl]-4-carbonyl)-3,3-difluoro-1-phenylazocan-2-one (3am)

Upon completion the mixture was concentrated and purified via flash column chromatography (petroleum ether : ethyl acetate = 5 : 1) to give the titled product **3am** as a **white solid** (62.1 mg, 74%, 95% purity).

^{1}H NMR (400 MHz, CDCl$_3$) δ 8.04 (d, *J* = 8.5 Hz, 2H), 7.74 (d, *J* = 8.4 Hz, 2H), 7.64 (d, *J* = 7.1 Hz, 2H), 7.51 – 7.41 (m, 6H), 7.36 (t, *J* = 7.4 Hz, 1H), 7.24 (s, 1H), 4.64 (ddt, *J* = 16.3, 12.5, 4.1 Hz, 1H), 3.72 (d, *J* = 15.7 Hz, 1H), 3.66 – 3.61 (m, 1H), 2.69 – 2.56 (m, 2H), 2.23 (d, *J* = 14.9 Hz, 1H), 1.98 – 1.90 (m, 1H), 1.82 – 1.67 (m, 2H).

^{13}C NMR (101 MHz, CDCl$_3$) δ 199.5, 164.45 (dd, *J* = 29.1, 25.6 Hz), 146.4, 141.7, 139.6, 133.5, 129.6, 129.05 (d, *J* = 4.2 Hz), 128.5, 127.9, 127.7, 127.3, 127.0, 118.57 (dd, *J* = 254.2, 245.2 Hz), 47.56 (d, *J* = 14.0 Hz), 40.74 (dd, *J* = 28.6, 23.4 Hz), 38.81 (d, *J* = 9.8 Hz), 27.1, 27.0.

^{19}F NMR (376 MHz, CDCl$_3$) δ -88.06 (d, *J* = 253.7 Hz), -99.36 (d, *J* = 254.4 Hz).

HRMS (ESI-TOF) m/z: $[M+H]^+$ Calcd. for $C_{26}H_{24}F_2NO_2$ 420.1770; found: 420.1774.

M.p. 167.5-169.1 °C

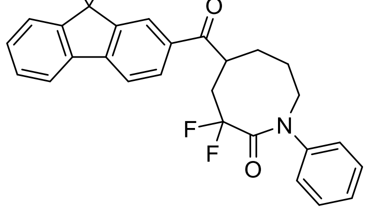

5-(9,9-dimethyl-9H-fluorene-2-carbonyl)-3,3-difluoro-1-phenylazocan-2-one (3an)

Upon completion the mixture was concentrated and purified via flash column chromatography (petroleum ether : ethyl acetate = 5 : 1) to give the titled product **3an** as a **yellow oil** (67.1 mg, 73%, 95% purity).

^{1}H NMR (400 MHz, CDCl$_3$) δ 8.07 (d, J = 1.6 Hz, 1H), 7.94 (dd, J = 8.0, 1.6 Hz, 1H), 7.83 – 7.79 (m, 2H), 7.50 – 7.43 (m, 3H), 7.42 – 7.34 (m, 3H), 7.27 (s, 1H), 7.25 (s, 1H), 4.69 – 4.62 (m, 1H), 3.75 (d, J = 12.8 Hz, 1H), 3.70 – 3.64 (m, 1H), 2.71 – 2.55 (m, 2H), 2.25 (d, J = 13.0 Hz, 1H), 1.98 – 1.91 (m, 1H), 1.72 – 1.62 (m, 2H), 1.53 (d, J = 3.1 Hz, 6H).

^{13}C NMR (101 MHz, CDCl$_3$) δ 199.8, 164.49 (dd, J = 29.0, 26.2 Hz), 154.8, 154.4, 144.8, 141.7, 137.6, 133.6, 129.6, 129.0, 127.90 (d, J = 4.9 Hz), 127.4, 127.1, 122.92 (d, J = 4.9 Hz), 121.2, 120.1, 118.58 (dd, J = 225.8, 217.3 Hz), 47.64 (d, J = 13.8 Hz), 47.1, 40.87 (dd, J = 28.5, 23.0 Hz), 38.84 (d, J = 9.6 Hz), 27.3, 27.0, 27.0.

^{19}F NMR (376 MHz, CDCl$_3$) δ -88.11 (d, J = 253.3 Hz), -99.33 (d, J = 245.6 Hz).

HRMS (ESI-TOF) m/z: $[M+H]^+$ Calcd. for $C_{29}H_{28}F_2NO_2$ 460.2083; found: 460.2089.

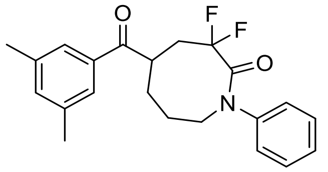

5-(3,5-dimethylbenzoyl)-3,3-difluoro-1-phenylazocan-2-one (3ao)

Upon completion the mixture was concentrated and purified via flash column chromatography (petroleum ether : ethyl acetate = 5 : 1) to give the titled product **3ao** as a **colorless oil** (38.6 mg, 52%, 95% purity).

^{1}H NMR (400 MHz, CDCl$_3$) δ 7.54 (s, 2H), 7.45 (t, J = 7.7 Hz, 3H), 7.35 (t, J = 7.6 Hz, 1H), 7.24 (d, J = 7.8 Hz, 2H), 4.66 – 4.58 (m, 1H), 3.70 (d, J = 14.4 Hz, 1H), 3.59 – 3.54 (m, 1H), 2.64 – 2.54 (m, 2H), 2.40 (s, 6H), 2.17 (d, J = 14.5 Hz, 1H), 1.94 – 1.86 (m, 1H), 1.66 – 1.61 (m, 2H).

^{13}C NMR (101 MHz, CDCl$_3$) δ 200.5, 164.46 (dd, J = 29.1, 26.0 Hz), 141.7, 138.7, 135.3, 135.1, 129.6, 129.5, 127.9, 127.0, 126.2, 118.61 (dd, J = 253.3, 244.4 Hz), 47.55 (d, J = 14.1 Hz), 40.73 (dd, J = 28.8, 22.9 Hz), 38.77 (d, J = 9.8 Hz), 27.1, 26.9, 21.3.

^{19}F NMR (376 MHz, CDCl$_3$) δ -88.13 (d, J = 253.5 Hz), -99.50 (d, J = 230.4 Hz).

HRMS (ESI-TOF) m/z: $[M+H]^+$ Calcd. for $C_{22}H_{24}F_2NO_2$ 372.1770; found: 372.1772.

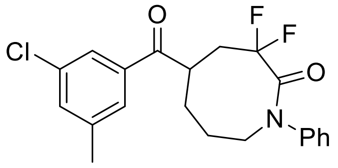

5-(3-chloro-5-methylbenzoyl)-3,3-difluoro-1-phenylazocan-2-one (3ap)

Upon completion the mixture was concentrated and purified via flash column chromatography (petroleum ether : ethyl acetate = 5 : 1) to give the titled product **3ap** as a **yellow oil** (47.8 mg, 61%, 95% purity).

^{1}H NMR (400 MHz, CDCl$_3$) δ 7.83 (d, J = 2.2 Hz, 1H), 7.69 (dd, J = 8.3, 2.2 Hz, 1H), 7.46 (dd, J = 15.0, 8.1 Hz, 3H), 7.35 (t, J = 7.4 Hz, 1H), 7.23 (d, J = 7.1 Hz, 2H), 4.65 – 4.56 (m, 1H), 3.70 (d, J = 16.5 Hz, 1H), 3.56 – 3.51 (m, 1H), 2.63 – 2.52 (m, 2H), 2.46 (s, 3H), 2.15 (d, J = 14.7 Hz, 1H), 1.95 – 1.88 (m, 1H), 1.81 – 1.66 (m, 2H).

^{13}C NMR (101 MHz, CDCl$_3$) δ 199.1, 164.39 (dd, J = 29.3, 26.0 Hz), 141.6, 140.3, 137.2, 133.3, 130.9, 129.7, 129.6, 128.0, 127.0, 126.9, 118.45 (t, J = 254.2 Hz), 47.49 (d, J = 14.1 Hz), 40.62 (dd, J = 29.1, 23.2 Hz), 38.79 (d, J = 9.9 Hz), 27.0, 26.9, 20.2.

HRMS (ESI-TOF) m/z: $[M+H]^+$ Calcd. for $C_{21}H_{21}ClF_2NO_2$ 392.1223; found: 392.1226.

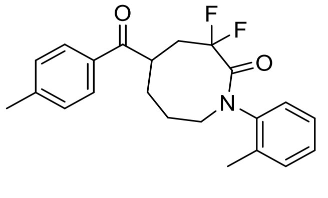

3,3-difluoro-5-(4-methylbenzoyl)-1-(*o*-tolyl)azocan-2-one (3bb)

Upon completion the mixture was concentrated and purified via flash column chromatography (petroleum ether : ethyl acetate = 5 : 1) to give the titled product **3bb** as a **yellow oil** (38.2 mg, 51%, 95% purity).

^{1}H NMR (400 MHz, CDCl$_3$) δ 7.87 (d, J = 8.3 Hz, 2H), 7.35 – 7.28 (m, 5H), 7.03 (d, J = 7.4 Hz, 1H), 5.00 – 4.62 (m, 1H), 3.75 – 3.55 (m, 1H), 3.32 (d, J = 15.8 Hz, 1H), 2.71 – 2.50 (m, 2H), 2.44 (s, 3H), 2.27 (s, 3H), 1.99 (d, J = 11.9 Hz, 1H), 1.86 – 1.79 (m, 1H), 1.59 (s, 2H).

^{13}C NMR (101 MHz, CDCl$_3$) δ 199.7, 164.44 (t, J = 28.3 Hz), 144.7, 140.2, 134.7,

132.4, 131.8, 129.8, 128.52 (d, J = 10.0 Hz), 127.7, 126.9, 118.39 (t, J = 248.2 Hz), 45.99 (d, J = 14.8 Hz), 40.86 (dd, J = 29.4, 22.3 Hz), 38.52 (d, J = 10.6 Hz), 27.3, 27.0, 21.7, 17.3.

^{19}F NMR (376 MHz, CDCl₃) δ -86.86 (d, J = 10.4 Hz), -87.54 (d, J = 13.5 Hz), -100.25 (d, J = 36.9 Hz), -100.92 (d, J = 43.5 Hz).

HRMS (ESI-TOF) m/z: $[M+H]^+$ Calcd. for $C_{22}H_{24}F_2NO_2$ 372.1770; found: 372.1772.

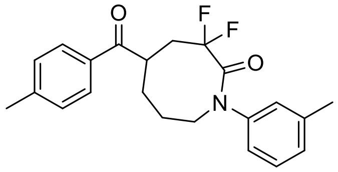

3,3-difluoro-5-(4-methylbenzoyl)-1-(*m*-tolyl)azocan-2-one (3cb)

Upon completion the mixture was concentrated and purified via flash column chromatography (petroleum ether : ethyl acetate = 5 : 1) to give the titled product **3cb** as a **colorless oil** (42.3 mg, 57%, 95% purity).

^{1}H NMR (400 MHz, CDCl₃) δ 7.86 (d, J = 8.2 Hz, 2H), 7.32 (d, J = 7.9 Hz, 3H), 7.15 (d, J = 7.7 Hz, 1H), 7.03 (dd, J = 10.9, 2.5 Hz, 2H), 4.63 – 4.55 (m, 1H), 3.70 – 3.64 (m, 1H), 3.60 – 3.54 (m, 1H), 2.64 – 2.52 (m, 2H), 2.44 (s, 3H), 2.38 (s, 3H), 2.18 (d, J = 14.4 Hz, 1H), 1.79 – 1.73 (m, 1H), 1.69 – 1.62 (m, 2H).

^{13}C NMR (101 MHz, CDCl₃) δ 199.7, 164.39 (dd, J = 29.0, 26.0 Hz), 144.6, 141.6, 139.7, 132.3, 129.7, 129.4, 128.7, 128.5, 127.6, 123.9, 118.60 (dd, J = 253.2, 244.6 Hz), 47.51 (d, J = 13.9 Hz), 40.76 (dd, J = 28.8, 23.0 Hz), 38.64 (d, J = 9.8 Hz), 27.2, 27.0, 21.7, 21.4.

^{19}F NMR (376 MHz, CDCl₃) δ -87.96 (d, J = 253.8 Hz), -99.33 (d, J = 256.8 Hz).

HRMS (ESI-TOF) m/z: $[M+H]^+$ Calcd. for $C_{22}H_{24}F_2NO_2$ 372.1770; found: 372.1772.

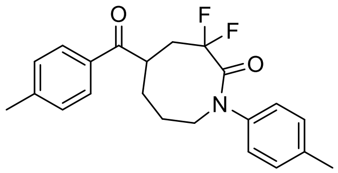

3,3-difluoro-5-(4-methylbenzoyl)-1-(*p*-tolyl)azocan-2-one (3db)

Upon completion the mixture was concentrated and purified via flash column chromatography (petroleum ether : ethyl acetate = 5 : 1) to give the titled product **3db**

as a **yellow oil** (30.4 mg, 41%, 95% purity).

^{1}H NMR (400 MHz, CDCl$_3$) δ 7.31 (d, J = 7.8 Hz, 2H), 7.24 (d, J = 8.0 Hz, 2H), 7.11 (d, J = 8.3 Hz, 2H), 4.63 – 4.54 (m, 1H), 3.69 – 3.64 (m, 1H), 3.59 – 3.54 (m, 1H), 2.65 – 2.55 (m, 2H), 2.44 (s, 3H), 2.37 (s, 3H), 2.17 (d, J = 12.1 Hz, 1H), 1.91 – 1.87 (m, 1H), 1.68 – 1.61 (m, 2H).

^{13}C NMR (101 MHz, CDCl$_3$) δ 199.7, 164.48 (dd, J = 29.2, 25.9 Hz), 144.6, 139.1, 137.8, 132.3, 130.2, 129.7, 128.5, 126.7, 118.65 (dd, J = 253.3, 244.4 Hz), 47.57 (d, J = 14.1 Hz), 40.75 (dd, J = 28.9, 23.1 Hz), 38.62 (d, J = 9.9 Hz), 27.2, 26.9, 21.7, 21.1.

^{19}F NMR (376 MHz, CDCl$_3$) δ -88.14 (d, J = 253.9 Hz), -99.41 (d, J = 253.4 Hz).

HRMS (ESI-TOF) m/z: $[M+H]^+$ Calcd. for $C_{22}H_{24}F_2NO_2$ 372.1770; found: 372.1772.

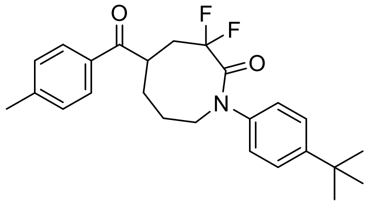

1-(4-(*tert*-butyl)phenyl)-3,3-difluoro-5-(4-methylbenzoyl)azocan-2-one (3eb)

Upon completion the mixture was concentrated and purified via flash column chromatography (petroleum ether : ethyl acetate = 5 : 1) to give the titled product **3eb** as a **yellow oil** (38.9 mg, 47%, 95% purity).

^{1}H NMR (400 MHz, CDCl$_3$) δ 7.86 (d, J = 8.2 Hz, 2H), 7.44 (d, J = 8.6 Hz, 2H), 7.32 (d, J = 8.0 Hz, 2H), 7.15 (d, J = 8.5 Hz, 2H), 4.63 – 4.55 (m, 1H), 3.69 (d, J = 15.3 Hz, 1H), 3.60 – 3.54 (m, 1H), 2.64 – 2.53 (m, 2H), 2.44 (s, 3H), 2.17 (d, J = 14.2 Hz, 1H), 1.95 – 1.88 (m, 1H), 1.66 – 1.61 (m, 2H), 1.33 (s, 9H).

^{13}C NMR (101 MHz, CDCl$_3$) δ 199.7, 164.50 (t, J = 30.3 Hz), 150.8, 144.6, 139.0, 132.3, 129.8, 128.6, 126.6, 126.4, 124.0, 118.73 (t, J = 242.6 Hz), 47.57 (d, J = 14.1 Hz), 40.80 (dd, J = 28.9, 23.1 Hz), 38.68 (d, J = 9.6 Hz), 34.7, 31.4, 27.2, 27.1, 21.7.

^{19}F NMR (376 MHz, CDCl$_3$) δ -88.05 (d, J = 250.1 Hz), -99.30 (d, J = 253.3 Hz).

HRMS (ESI-TOF) m/z: $[M+H]^+$ Calcd. for $C_{25}H_{30}F_2NO_2$ 414.2239; found: 414.2243.

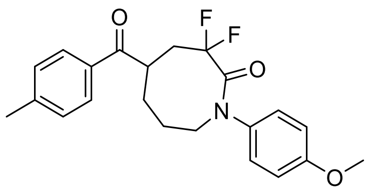

3,3-difluoro-1-(4-methoxyphenyl)-5-(4-methylbenzoyl)azocan-2-one (3fb)

Upon completion the mixture was concentrated and purified via flash column chromatography (petroleum ether : ethyl acetate = 5 : 1) to give the titled product **3fb** as a **yellow oil** (31.8 mg, 41%, 95% purity).

^{1}H NMR (400 MHz, CDCl$_3$) δ 7.85 (d, J = 8.2 Hz, 2H), 7.31 (d, J = 8.0 Hz, 2H), 7.14 (d, J = 8.9 Hz, 2H), 6.95 (d, J = 8.9 Hz, 2H), 4.63 – 4.55 (m, 1H), 3.82 (s, 3H), 3.66 – 3.62 (m, 1H), 3.59 – 3.56 (m, 1H), 2.63 – 2.47 (m, 2H), 2.44 (s, 3H), 2.17 (d, J = 14.2 Hz, 1H), 1.90 (t, J = 13.3 Hz, 1H), 1.69 – 1.61 (m, 2H).

^{13}C NMR (101 MHz, CDCl$_3$) δ 199.7, 164.65 (dd, J = 29.1, 25.9 Hz), 158.9, 144.6, 134.4, 132.3, 129.7, 128.5, 128.0, 118.70 (dd, J = 254.1, 245.5 Hz), 114.8, 55.5, 47.70 (d, J = 14.0 Hz), 40.78 (dd, J = 29.2, 23.0 Hz), 38.62 (d, J = 9.9 Hz), 27.1, 26.9, 21.7.

^{19}F NMR (376 MHz, CDCl$_3$) δ -87.96 (d, J = 254.0 Hz), -99.37 (d, J = 254.5 Hz).

HRMS (ESI-TOF) m/z: $[M+H]^+$ Calcd. for $C_{22}H_{24}F_2NO_3$ 388.1719; found: 388.1722.

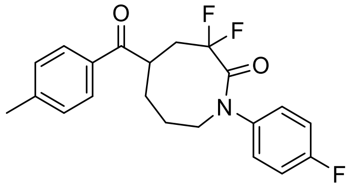

3,3-difluoro-1-(4-fluorophenyl)-5-(4-methylbenzoyl)azocan-2-one (3gb)

Upon completion the mixture was concentrated and purified via flash column chromatography (petroleum ether : ethyl acetate = 5 : 1) to give the titled product **3gb** as a **yellow oil** (28.5 mg, 38%, 95% purity).

^{1}H NMR (400 MHz, CDCl$_3$) δ 7.85 (d, J = 8.2 Hz, 2H), 7.31 (d, J = 8.0 Hz, 2H), 7.21 (dd, J = 8.9, 4.8 Hz, 2H), 7.13 (t, J = 8.6 Hz, 2H), 4.66 – 4.57 (m, 1H), 3.64 (d, J = 16.0 Hz, 1H), 3.59 – 3.54 (m, 1H), 2.64 – 2.50 (m, 2H), 2.44 (s, 3H), 2.19 (d, J = 9.1 Hz, 1H), 1.92 – 1.85 (m, 1H), 1.69 – 1.62 (m, 2H).

^{13}C NMR (101 MHz, CDCl$_3$) δ 199.6, 164.71 (t, J = 30.5 Hz), 163.0, 160.5, 144.7, 137.6, 132.3, 129.8, 128.87 (d, J = 8.7 Hz), 128.6, 118.54 (t, J = 252.6 Hz), 116.7, 116.5,

47.72 (d, J = 14.3 Hz), 40.80 (dd, J = 28.9, 22.6 Hz), 38.57 (d, J = 9.9 Hz), 27.1, 26.9, 21.7.

^{19}F NMR (376 MHz, CDCl$_3$) δ -88.58 (d, J = 253.3 Hz), -100.07 (d, J = 278.8 Hz), -113.84.

HRMS (ESI-TOF) m/z: [M+H]$^+$ Calcd. for $C_{21}H_{21}F_3NO_2$ 376.1519; found: 376.1523.

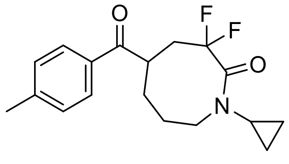

1-cyclopropyl-3,3-difluoro-5-(4-methylbenzoyl)azocan-2-one (3hb)

Upon completion the mixture was concentrated and purified via flash column chromatography (petroleum ether : ethyl acetate = 5 : 1) to give the titled product **3hb** as a **colorless oil** (52.1 mg, 81%, 95% purity).

^{1}H NMR (400 MHz, CDCl$_3$) δ 7.81 (d, J = 8.3 Hz, 2H), 7.29 (d, J = 8.0 Hz, 2H), 4.25 – 4.16 (m, 1H), 3.40 (d, J = 12.4 Hz, 2H), 2.70 – 2.64 (m, 1H), 2.53 – 2.46 (m, 1H), 2.42 (s, 3H), 2.04 (d, J = 9.8 Hz, 2H), 1.71 – 1.61 (m, 2H), 1.48 – 1.38 (m, 1H), 1.09 – 1.03 (m, 1H), 0.79 – 0.73 (m, 2H), 0.65 – 0.57 (m, 1H).

^{13}C NMR (101 MHz, CDCl$_3$) δ 199.6, 167.68 (t, J = 25.3 Hz), 144.6, 132.4, 129.7, 128.5, 118.47 (t, J = 255.5 Hz), 44.57 (d, J = 14.7 Hz), 40.57 (dd, J = 28.8, 22.6 Hz), 38.42 (d, J = 10.3 Hz), 30.1, 27.3, 26.5, 21.7, 9.4, 5.8.

^{19}F NMR (376 MHz, CDCl$_3$) δ -90.19 (d, J = 258.5 Hz), -102.30 (d, J = 249.6 Hz).

HRMS (ESI-TOF) m/z: [M+H]$^+$ Calcd. for $C_{18}H_{22}F_2NO_2$ 322.1613; found: 322.1621.

5. Reference

[1] Ma, H.; Hou, C.-Y.; Jiang, Y.; Qi, X.; Wu, X.-F. Nickel-Catalyzed Carbonylative Cyclization of Bromodifluoroacetamides with Arylboronic Acids toward ***δ*-**Lactams. *Org. Lett.* **2025**, *27*, 10136-10140.

[2] Huo, X.; Yu, B.; Huang, H. Iron-Catalyzed Alkylative Aminosulfonylation of Alkenes and Alkynes via Radical-Anion Relay. *Org. Lett.* **2025**, *27*, 5343-5348.

6. Copy of ^{1}H ,^{13}C and ^{19}F NMR Spectra of Bromodifluoro acetamides (1a-1h).

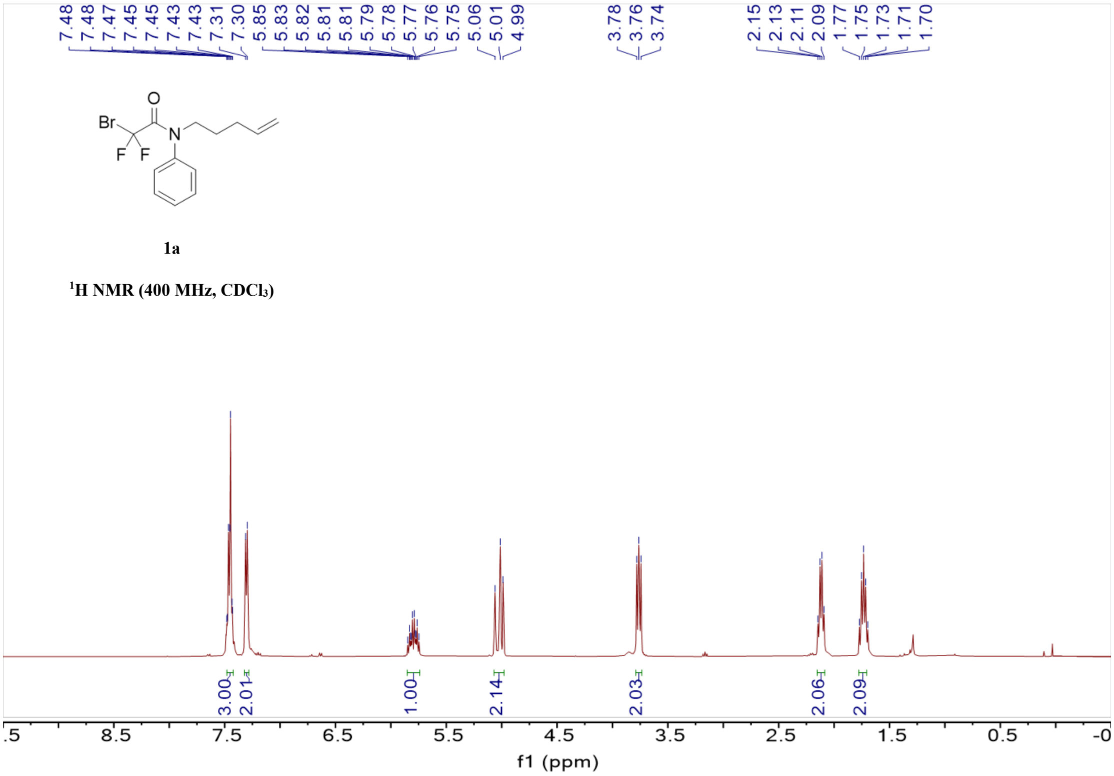

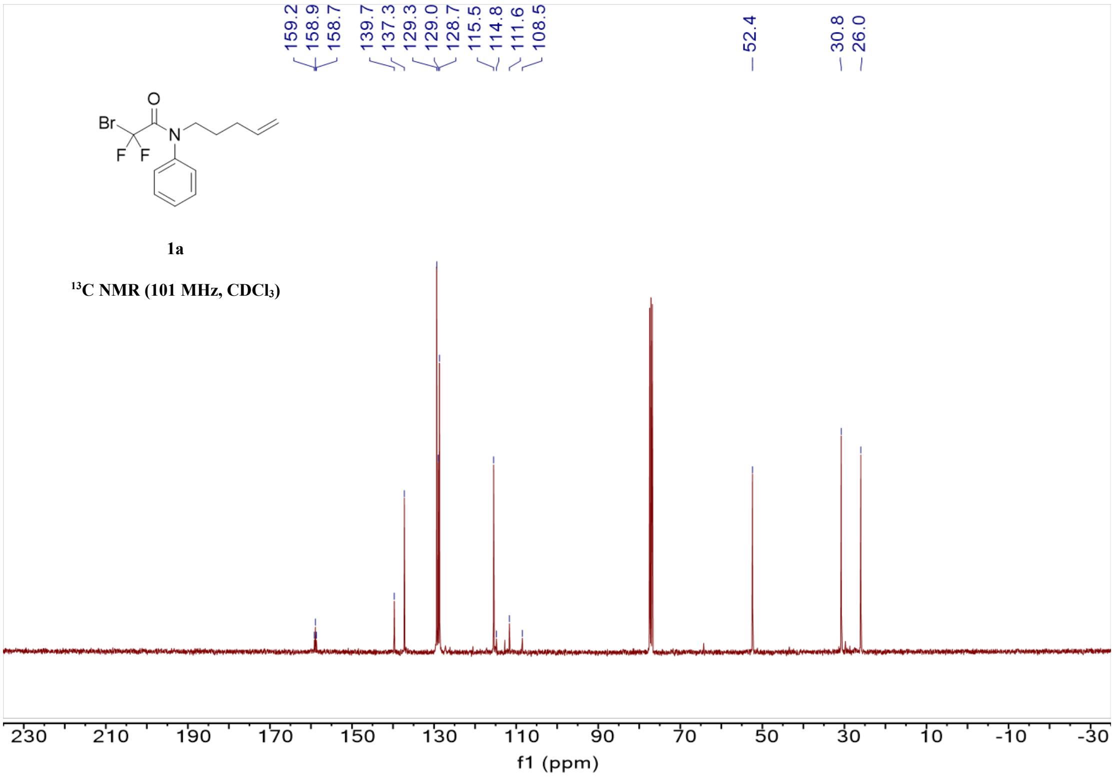

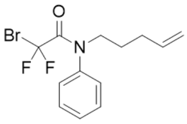

1a

^{19}F NMR (376 MHz, $CDCl_3$)

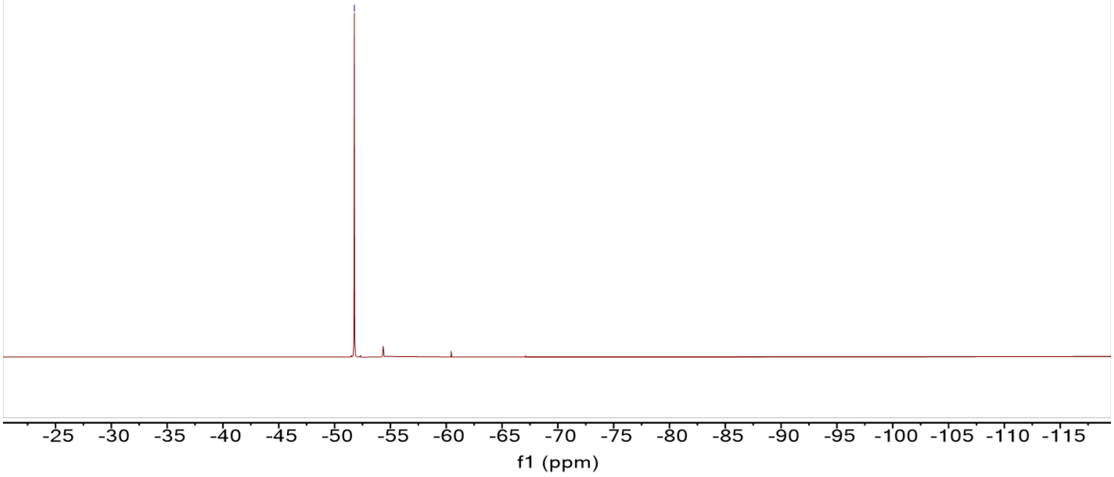

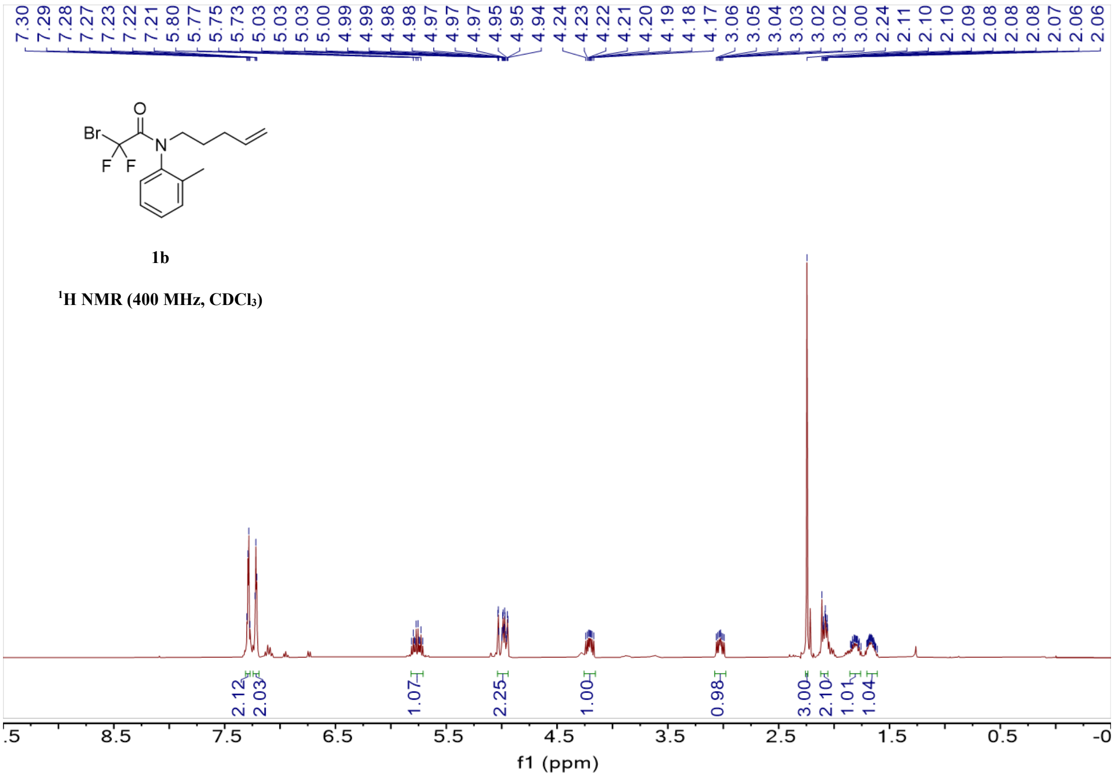

1b

^{1}H NMR (400 MHz, $CDCl_3$)

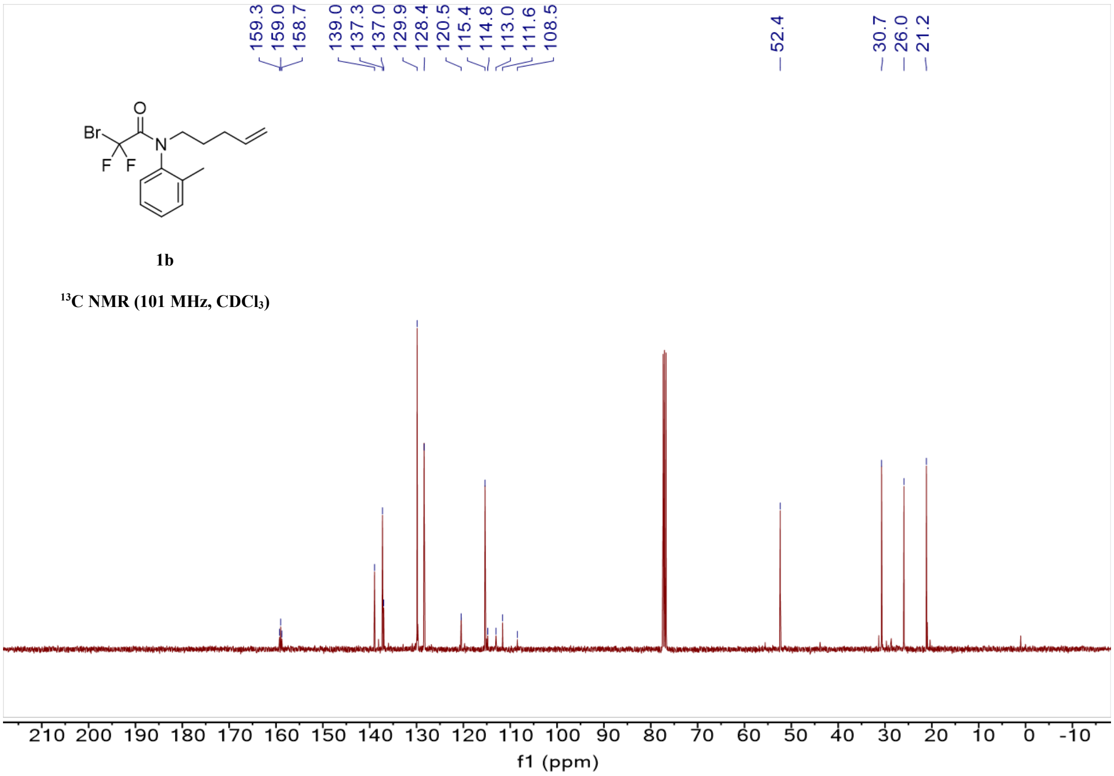

1b

^{13}C NMR (101 MHz, $CDCl_3$)

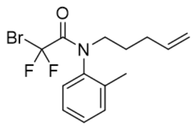

1b

^{19}F NMR (376 MHz, CDCl$_3$)

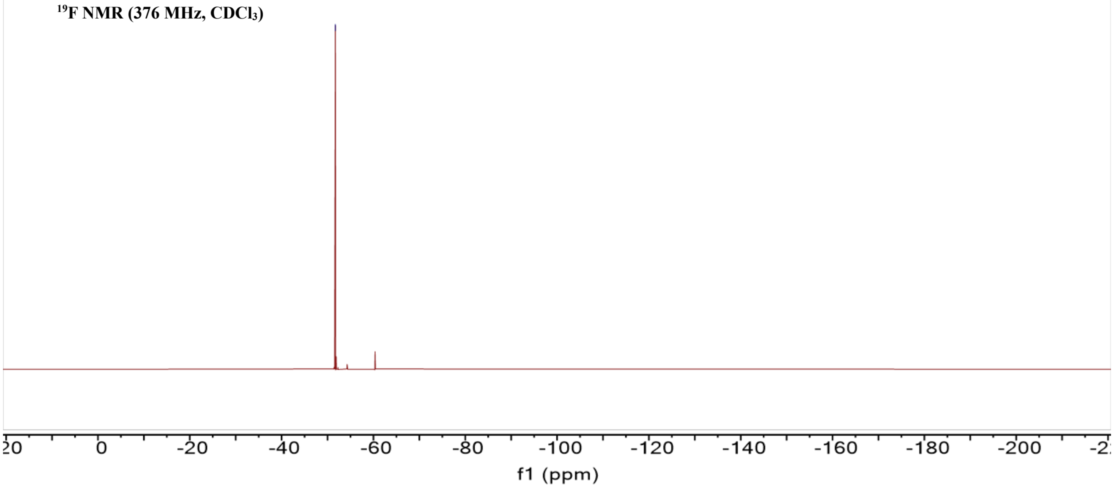

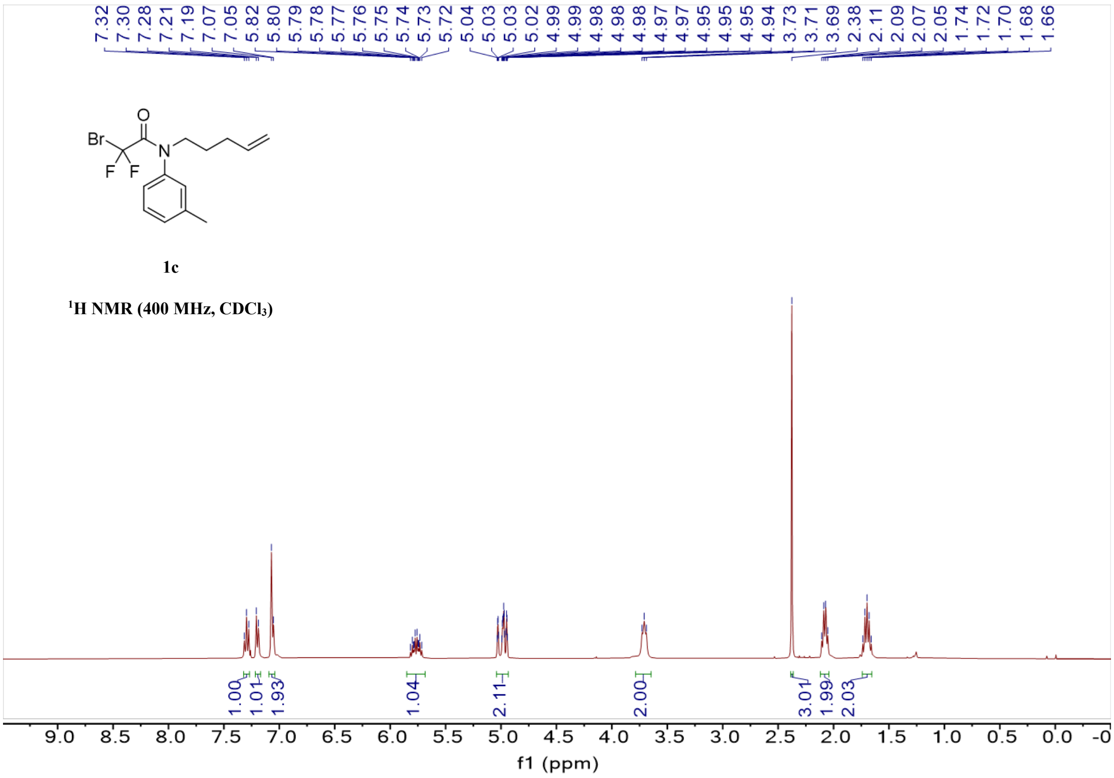

1c

^{1}H NMR (400 MHz, CDCl$_3$)

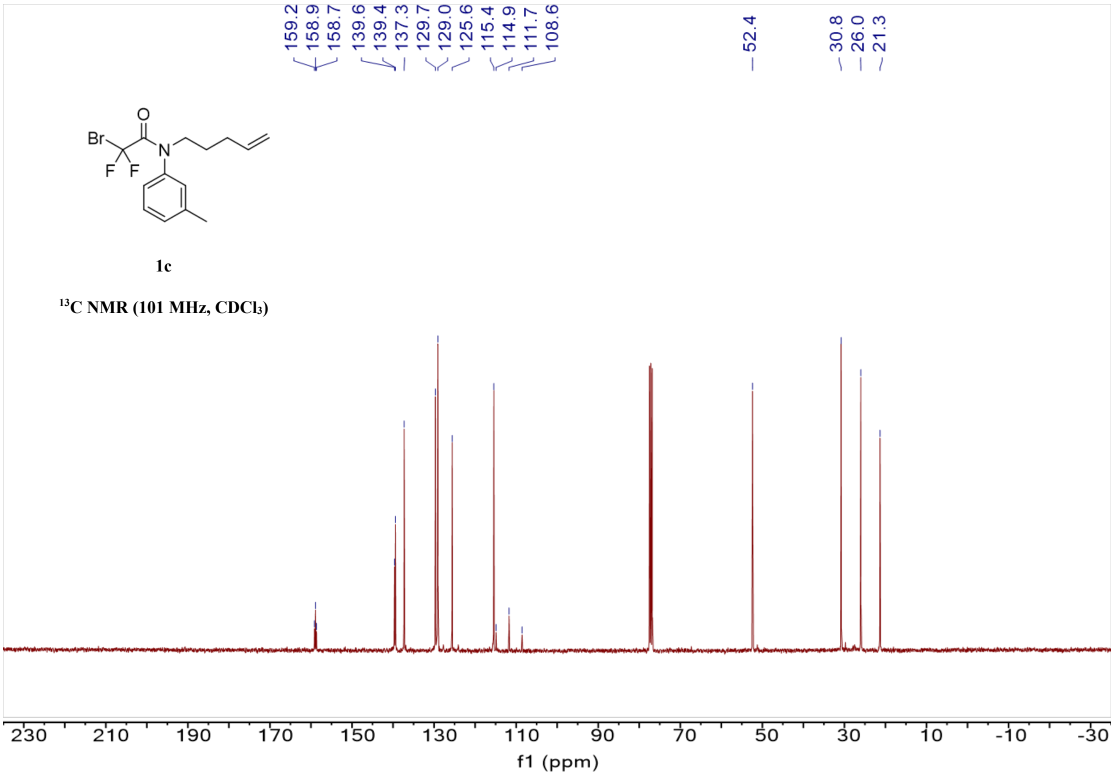

1c

^{13}C NMR (101 MHz, CDCl$_3$)

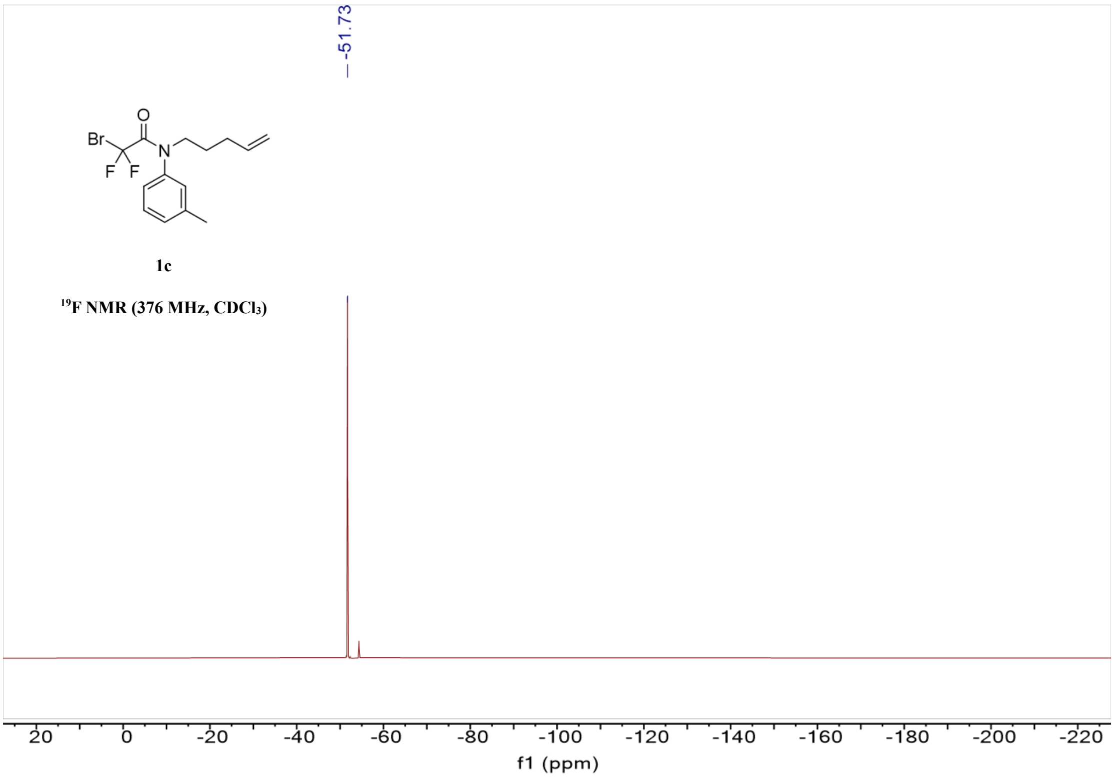

-51.73
Br
O
N
F F
1c
19F NMR (376 MHz, CDCl3)
20 0 -20 -40 -60 -80 -100 -120 -140 -160 -180 -200 -220
f1 (ppm)

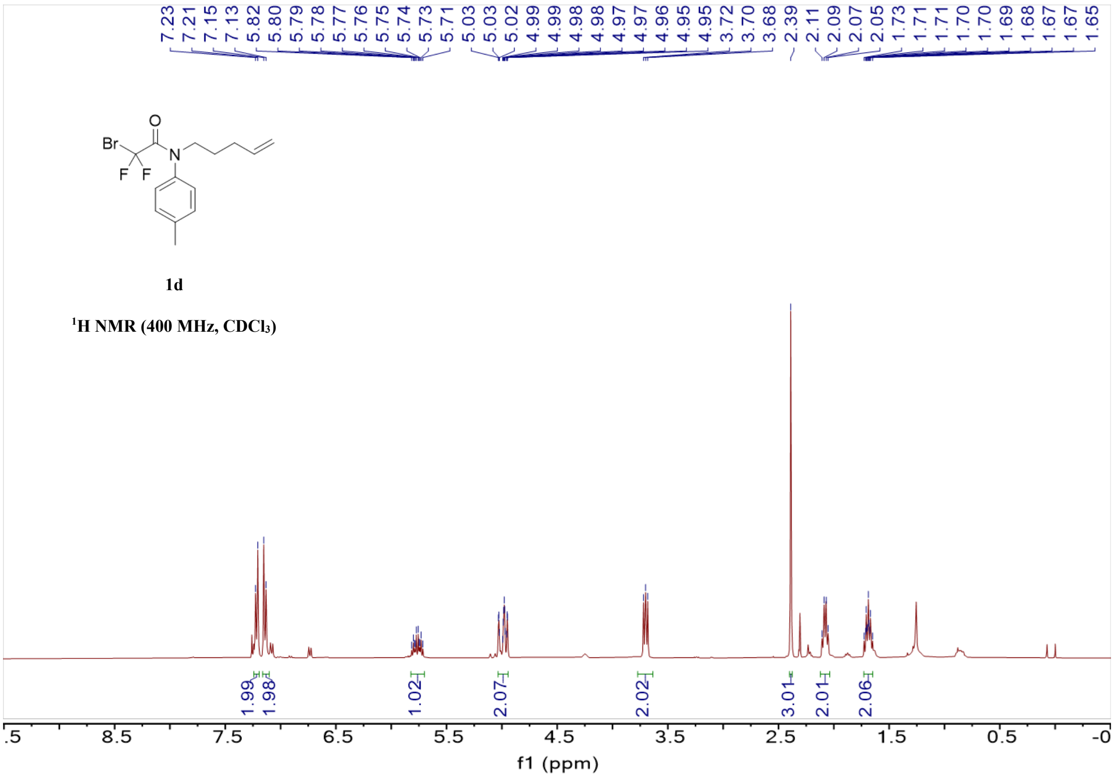

7.23 7.21 7.15 7.13 5.82 5.80 5.79 5.78 5.77 5.76 5.75 5.74 5.73 5.71 5.03 5.03 5.02 4.99 4.99 4.98 4.98 4.97 4.97 4.96 4.95 4.95 3.72 3.70 3.68 2.39 2.11 2.09 2.07 2.05 1.73 1.71 1.71 1.70 1.70 1.69 1.68 1.67 1.65
Br
O
N
F F
1d
1H NMR (400 MHz, CDCl3)
1.99 1.98 1.02 2.07 2.02 3.01 2.01 2.06
f1 (ppm)

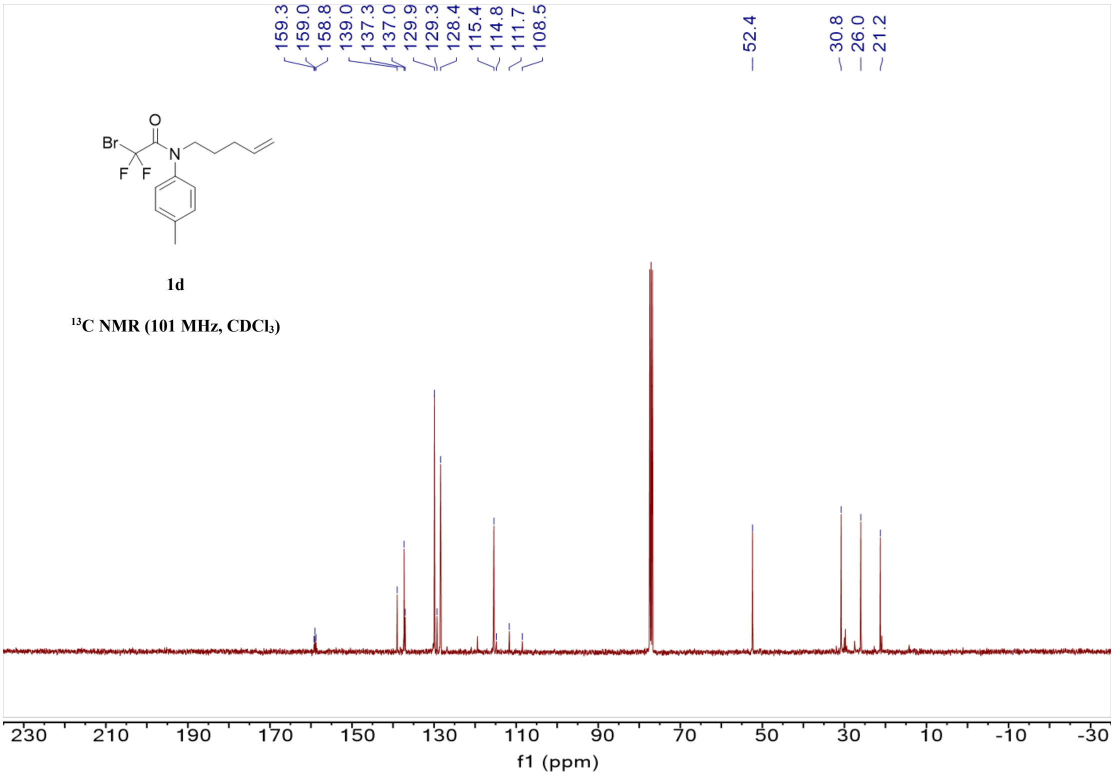

159.3 159.0 158.8 139.0 137.3 137.0 129.9 129.3 128.4 115.4 114.8 111.7 108.5 52.4 30.8 26.0 21.2
Br
O
N
F F
1d
13C NMR (101 MHz, CDCl3)
f1 (ppm)

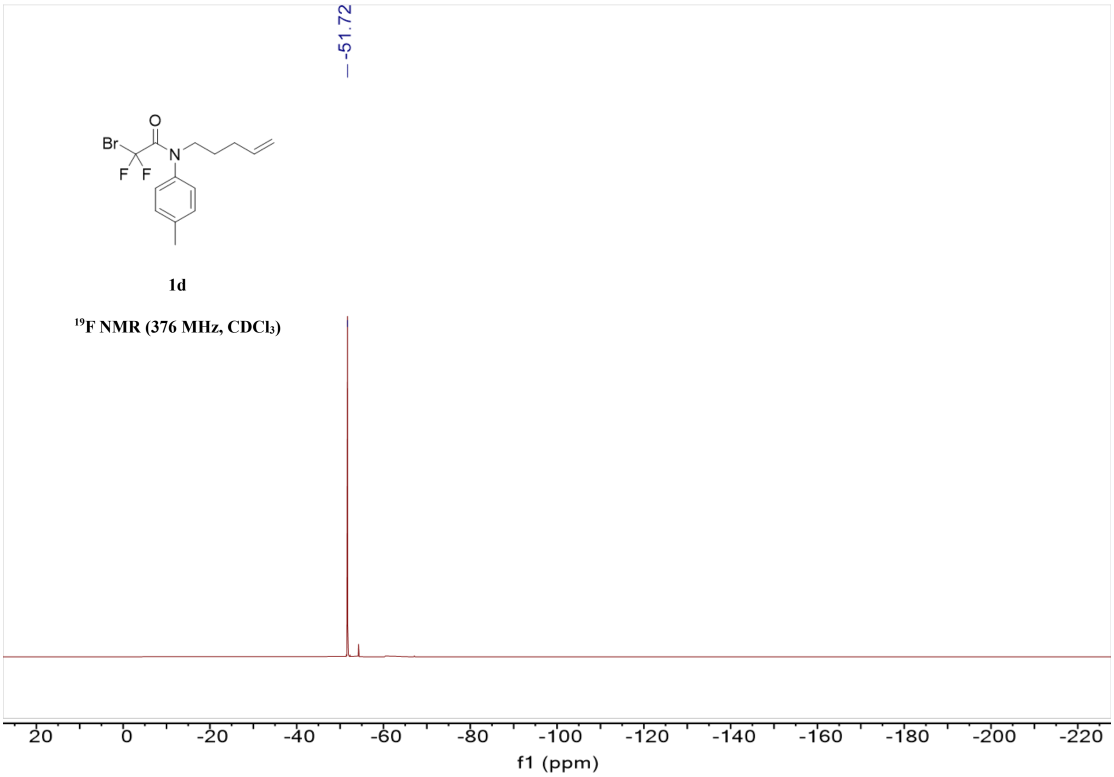

-51.72
Br
O
N
F F
1d
19F NMR (376 MHz, CDCl3)
20 0 -20 -40 -60 -80 -100 -120 -140 -160 -180 -200 -220
f1 (ppm)

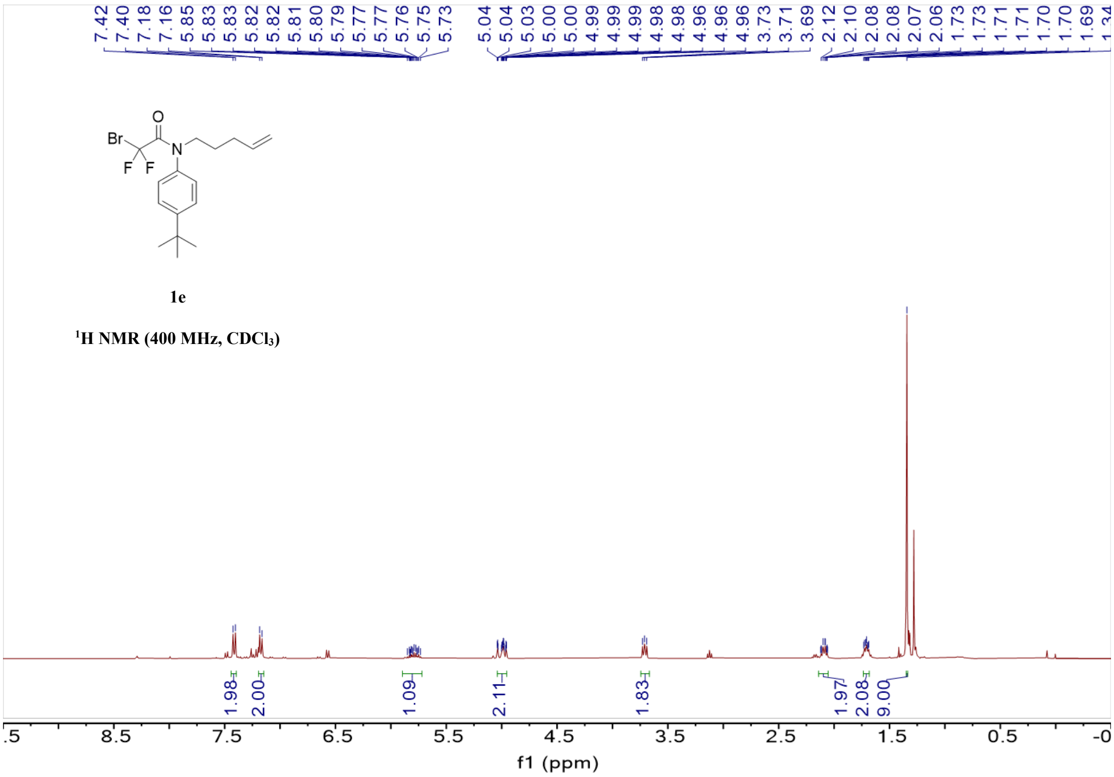

1e

^{1}H NMR (400 MHz, CDCl$_3$)

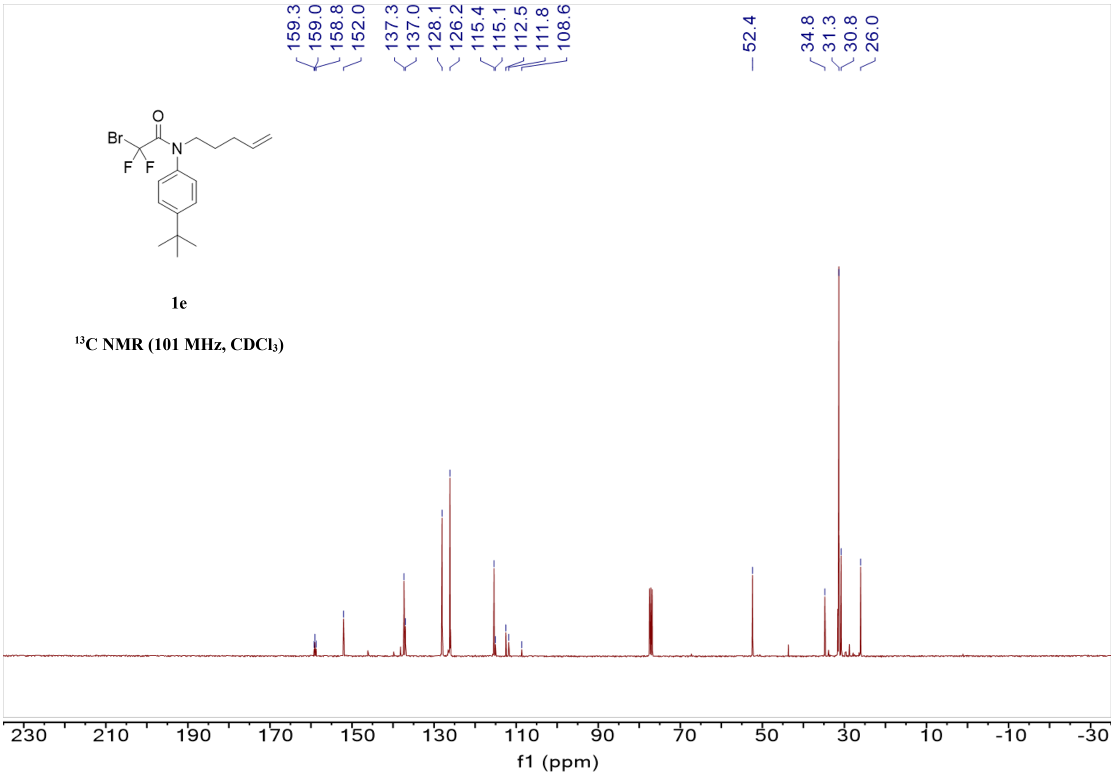

1e

^{13}C NMR (101 MHz, CDCl$_3$)

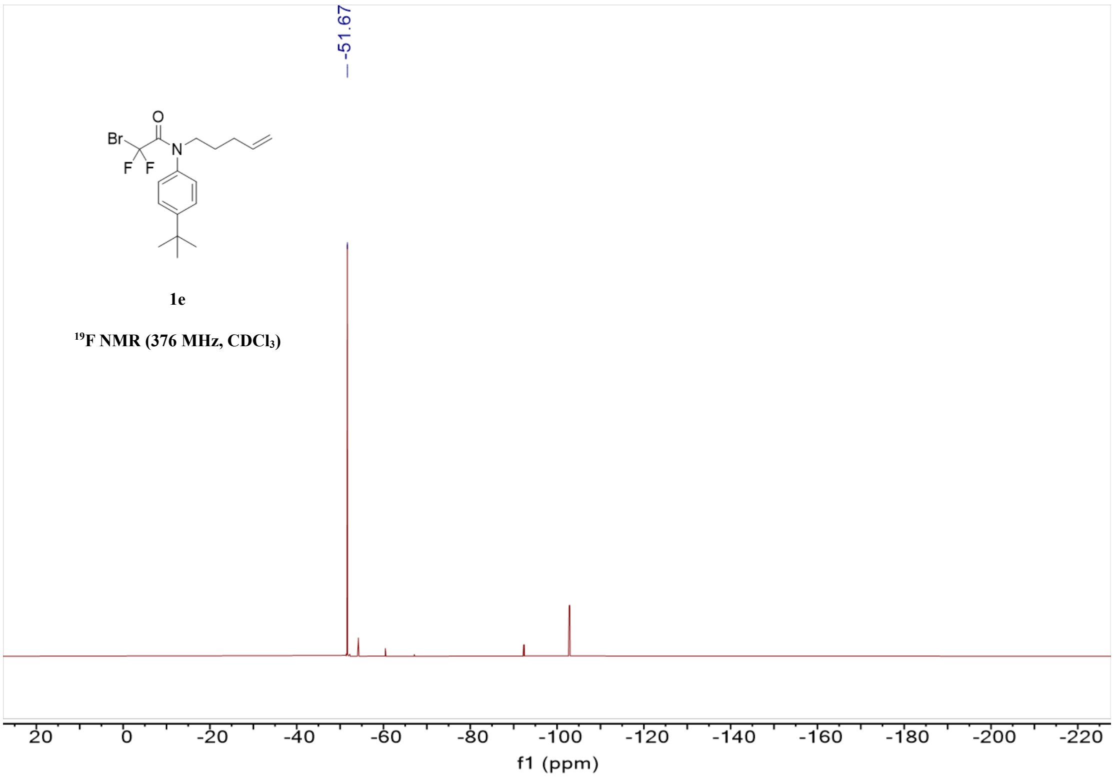

1e

^{19}F NMR (376 MHz, $CDCl_3$)

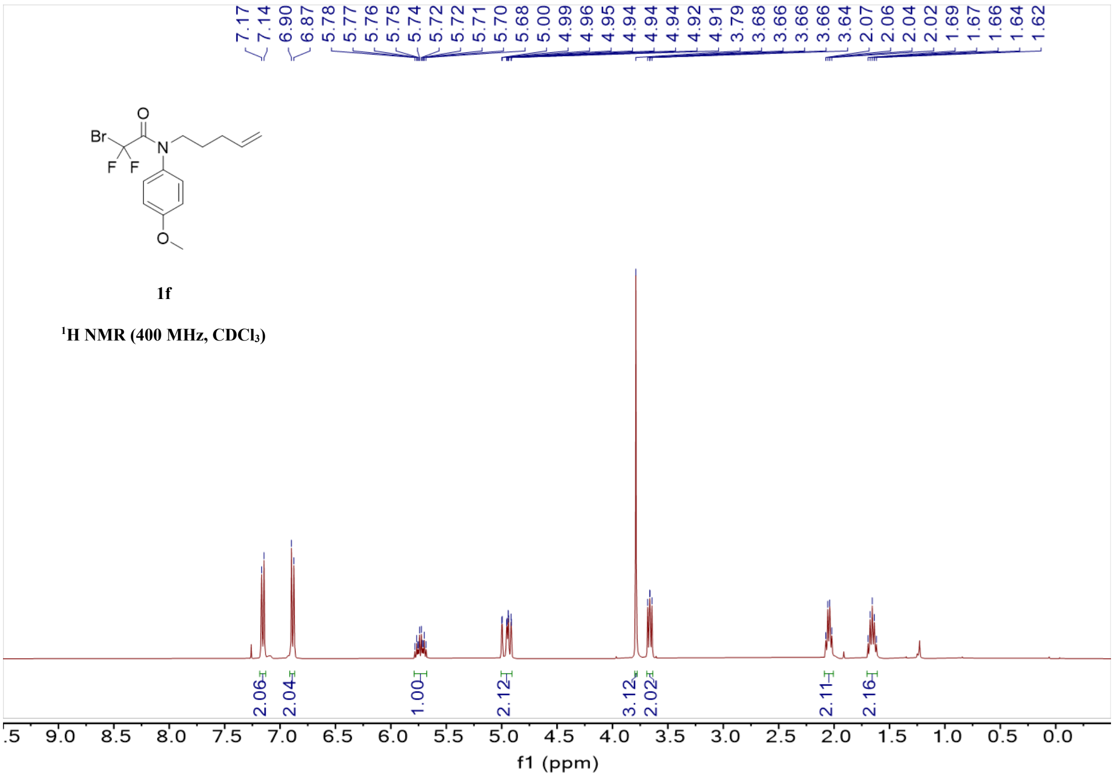
7.17
7.14
6.90
6.87
5.78
5.77
5.76
5.75
5.74
5.72
5.72
5.71
5.70
5.68
5.00
4.99
4.96
4.95
4.94
4.94
4.94
4.92
4.91
3.79
3.68
3.66
3.66
3.64
2.07
2.06
2.04
2.02
1.69
1.67
1.66
1.64
1.62
Br
O
N
F F
O
1f
1H NMR (400 MHz, CDCl3)
2.06
2.04
1.00
2.12
3.12
2.02
2.11
2.16
.5 9.0 8.5 8.0 7.5 7.0 6.5 6.0 5.5 5.0 4.5 4.0 3.5 3.0 2.5 2.0 1.5 1.0 0.5 0.0
f1 (ppm)

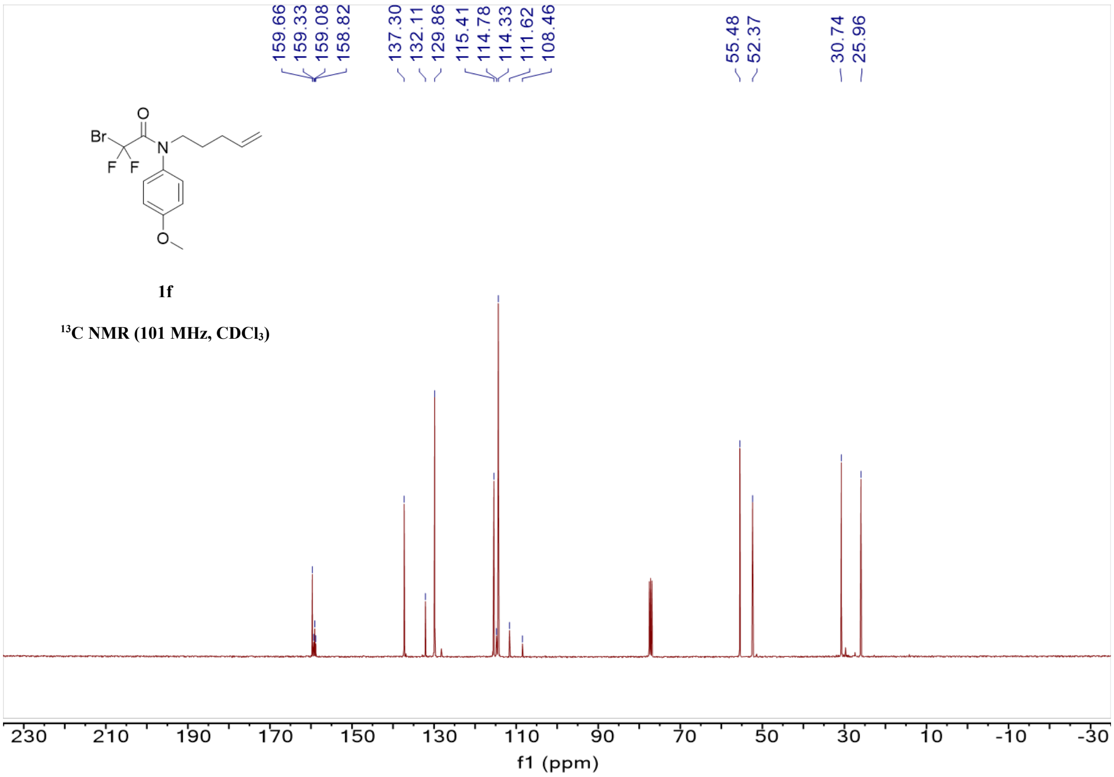
159.66
159.33
159.08
158.82
137.30
132.11
129.86
115.41
114.78
114.33
111.62
108.46
55.48
52.37
30.74
25.96
Br
O
N
F F
O
1f
13C NMR (101 MHz, CDCl3)
230 210 190 170 150 130 110 90 70 50 30 10 -10 -30
f1 (ppm)

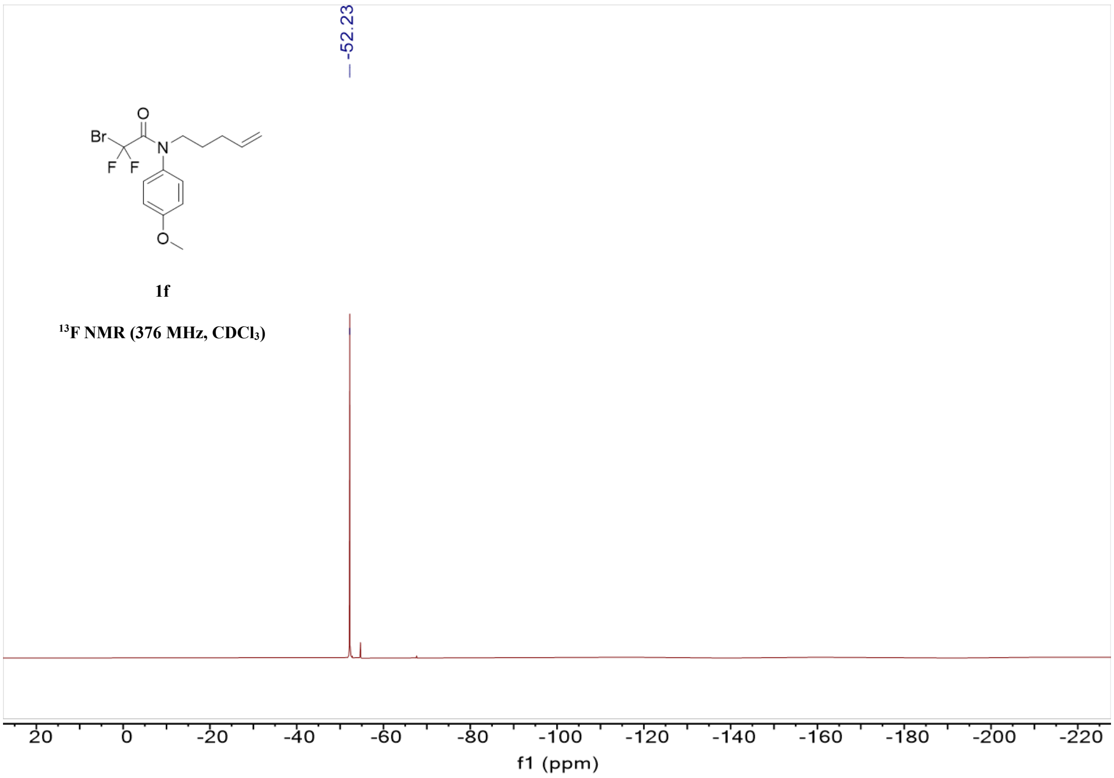

-52.23
Br
O
N
F F
O
1f
13F NMR (376 MHz, CDCl3)
20 0 -20 -40 -60 -80 -100 -120 -140 -160 -180 -200 -220
f1 (ppm)

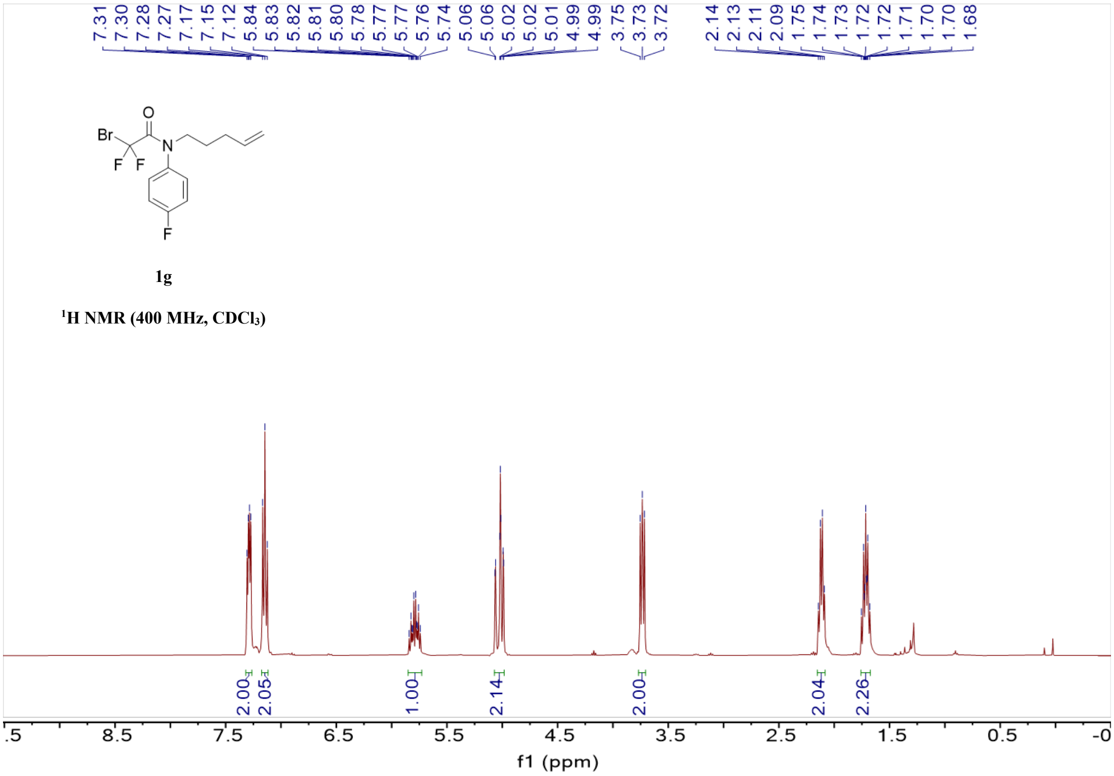
7.31 7.30 7.28 7.27 7.17 7.15 7.12 5.84 5.83 5.82 5.81 5.80 5.78 5.77 5.77 5.76 5.74 5.06 5.06 5.02 5.02 5.01 4.99 4.99 3.75 3.73 3.72 2.14 2.13 2.11 2.09 1.75 1.74 1.73 1.72 1.72 1.71 1.70 1.70 1.68
Br
O
N
F F
F
1g
^{1}H NMR (400 MHz, CDCl$_3$)
2.00 2.05 1.00 2.14 2.00 2.04 2.26
.5 8.5 7.5 6.5 5.5 4.5 3.5 2.5 1.5 0.5 -0
f1 (ppm)

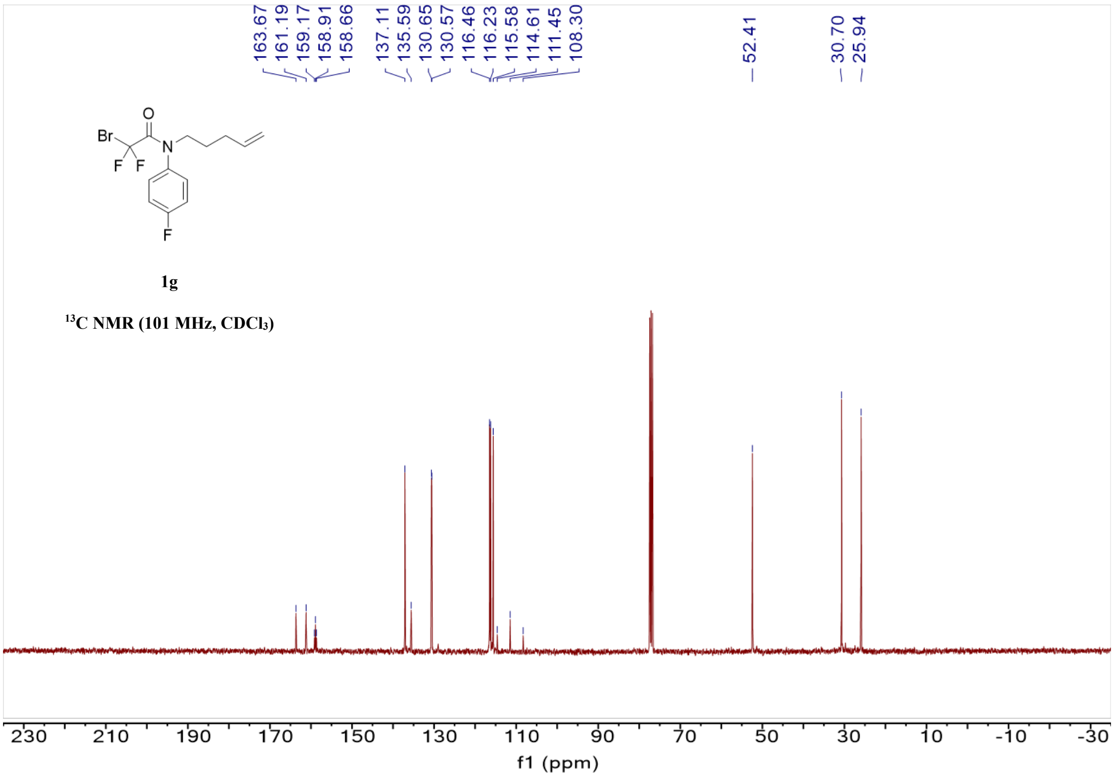
163.67 161.19 159.17 158.91 158.66 137.11 135.59 130.65 130.57 116.46 116.23 115.58 114.61 111.45 108.30 52.41 30.70 25.94
Br
O
N
F F
F
1g
^{13}C NMR (101 MHz, CDCl$_3$)
230 210 190 170 150 130 110 90 70 50 30 10 -10 -30
f1 (ppm)

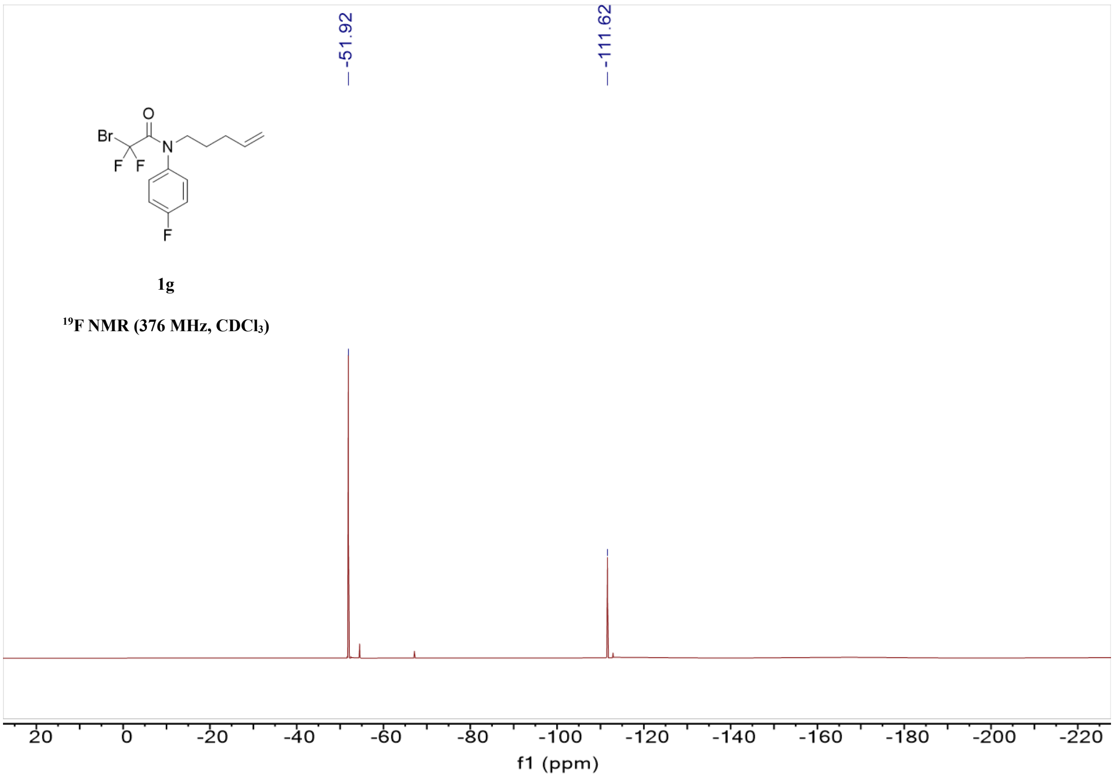

1g

^{19}F NMR (376 MHz, $CDCl_3$)

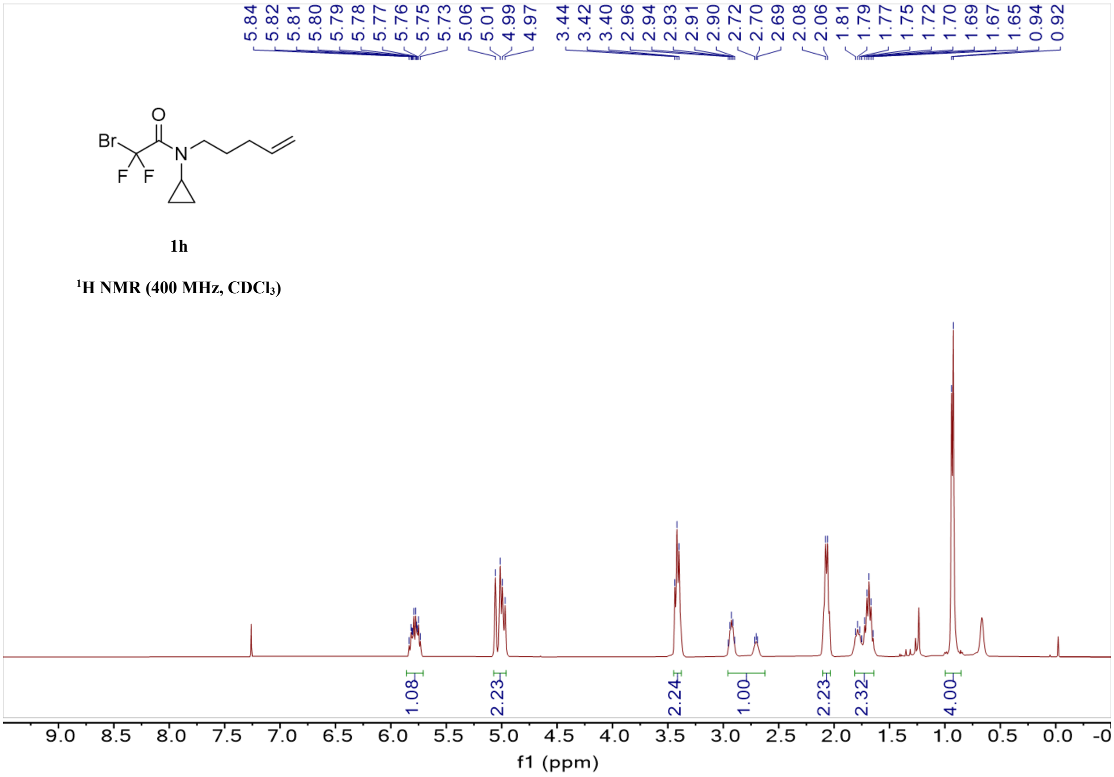
5.84 5.82 5.81 5.80 5.79 5.78 5.77 5.76 5.75 5.73 5.06 5.01 4.99 4.97 3.44 3.42 3.40 2.96 2.94 2.93 2.91 2.90 2.72 2.70 2.69 2.08 2.06 1.81 1.79 1.77 1.75 1.72 1.70 1.69 1.67 1.65 0.94 0.92
Br
O
N
F F
1h
1H NMR (400 MHz, CDCl3)
1.08 2.23 2.24 1.00 2.23 2.32 4.00
9.0 8.5 8.0 7.5 7.0 6.5 6.0 5.5 5.0 4.5 4.0 3.5 3.0 2.5 2.0 1.5 1.0 0.5 0.0 -0
f1 (ppm)

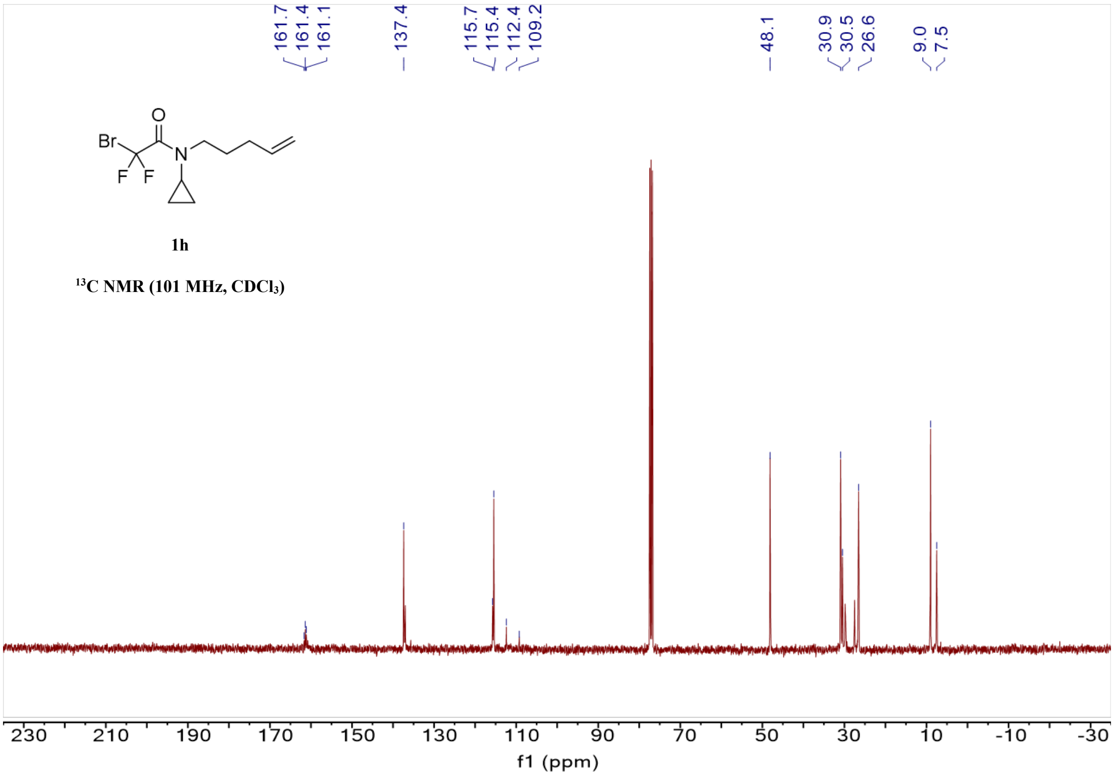
161.7 161.4 161.1 137.4 115.7 115.4 112.4 109.2 48.1 30.9 30.5 26.6 9.0 7.5
Br
O
N
F F
1h
13C NMR (101 MHz, CDCl3)
230 210 190 170 150 130 110 90 70 50 30 10 -10 -30
f1 (ppm)

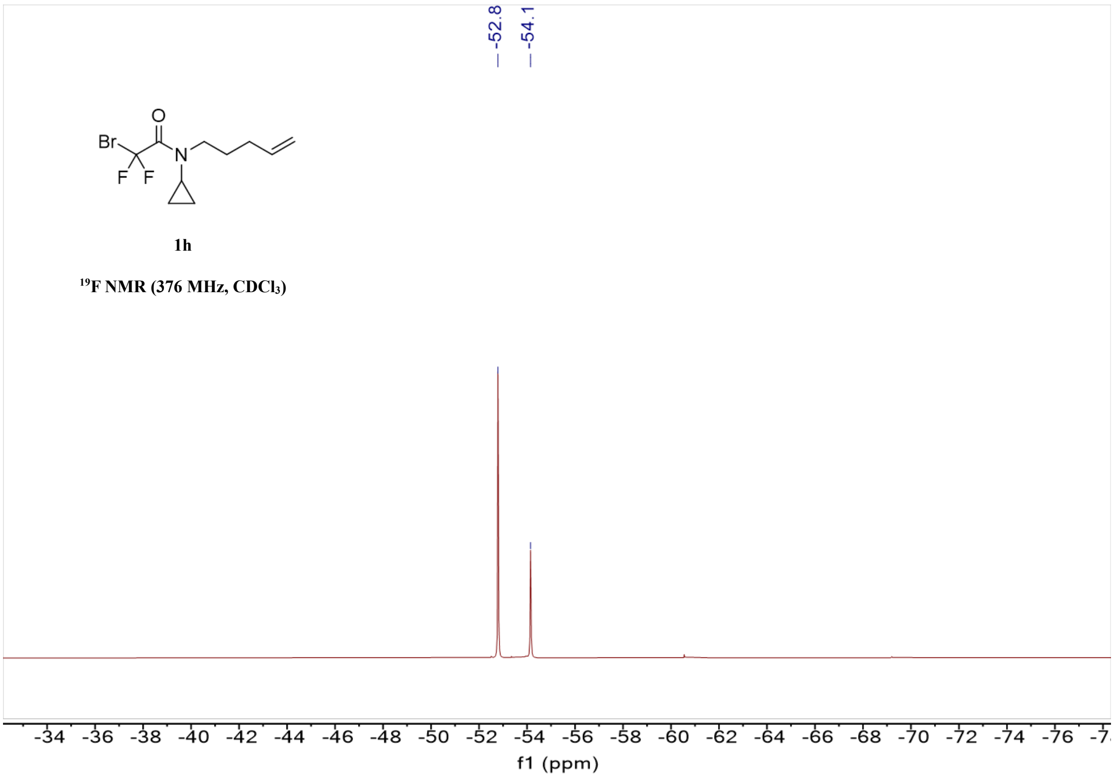

-52.8
-54.1
Br
O
N
F F
1h
19F NMR (376 MHz, CDCl3)
-34 -36 -38 -40 -42 -44 -46 -48 -50 -52 -54 -56 -58 -60 -62 -64 -66 -68 -70 -72 -74 -76
f1 (ppm)

7. Copy of ^{1}H , ^{13}C and ^{19}F NMR Spectra of Products.

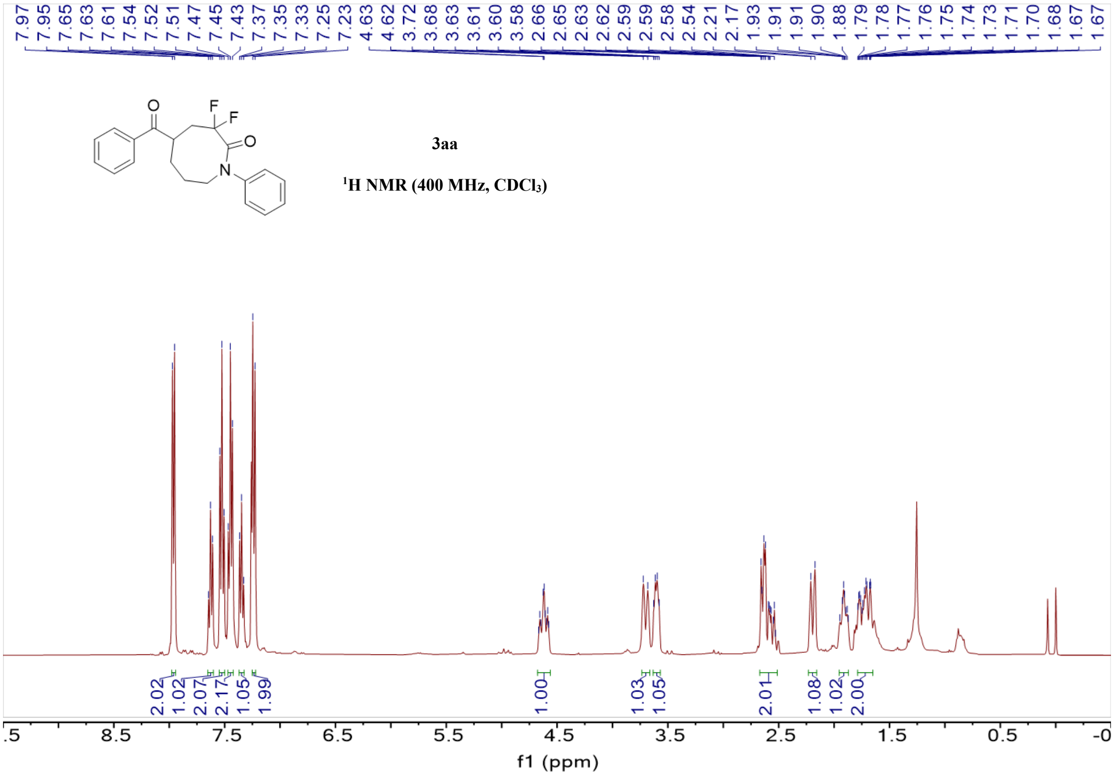

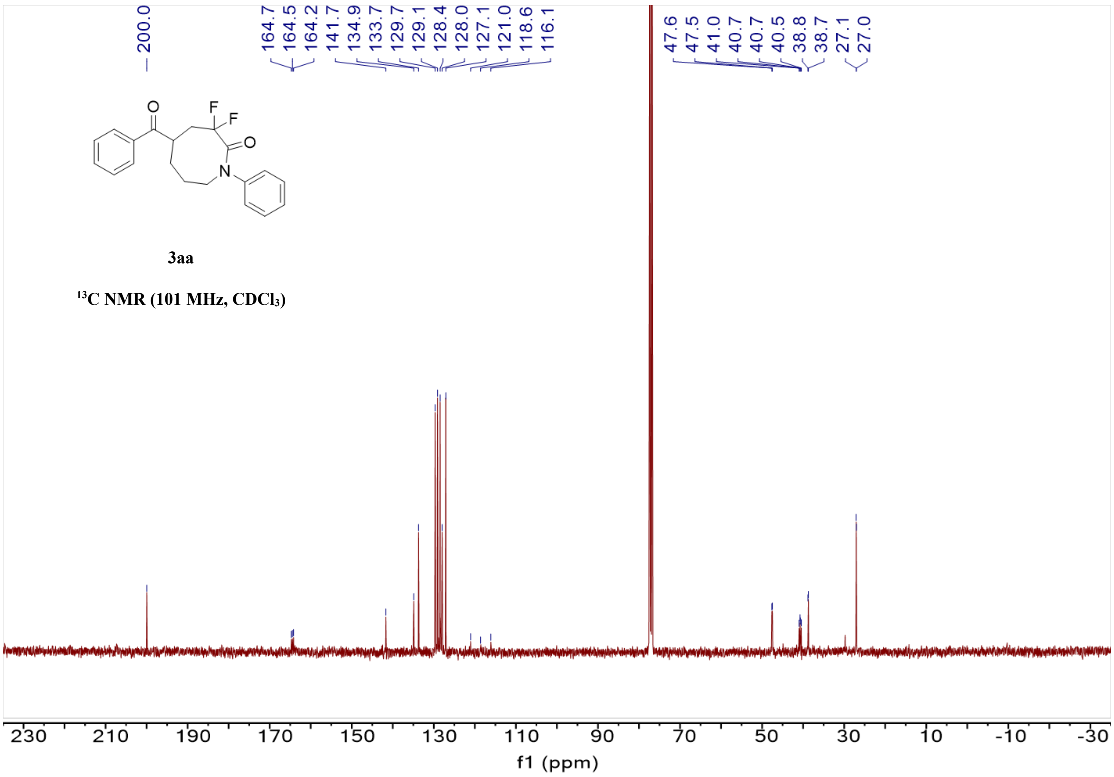

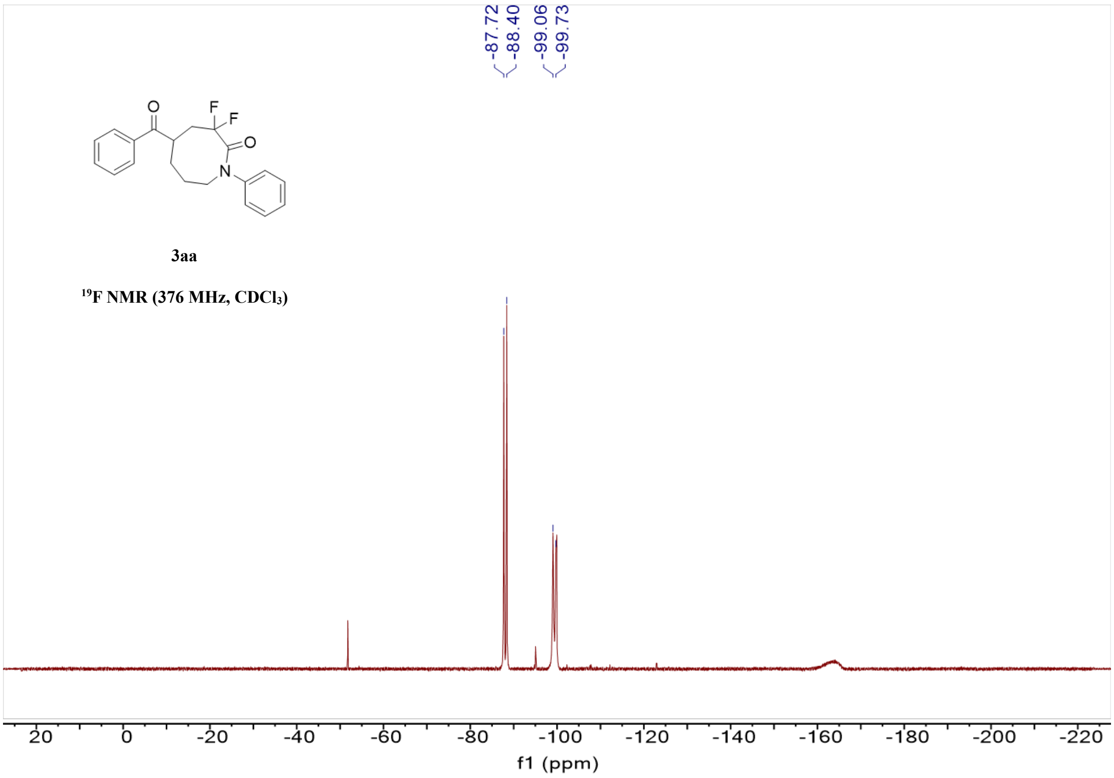

3aa

^{19}F NMR (376 MHz, CDCl$_3$)

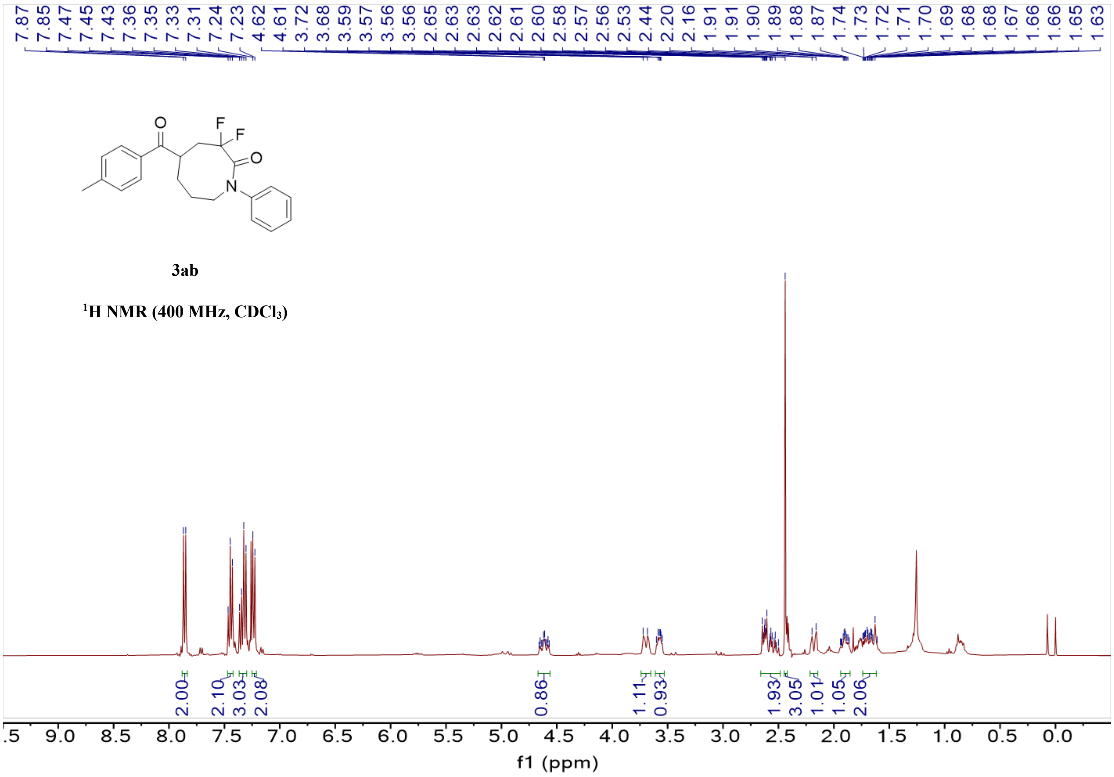
7.87 7.85 7.47 7.45 7.43 7.36 7.35 7.33 7.31 7.24 7.23 4.62 4.61 3.72 3.68 3.59 3.57 3.56 3.56 2.65 2.63 2.63 2.62 2.61 2.60 2.58 2.57 2.56 2.53 2.44 2.20 2.16 1.91 1.91 1.90 1.89 1.88 1.87 1.74 1.73 1.72 1.71 1.70 1.69 1.68 1.68 1.67 1.66 1.66 1.65 1.63
F
F
O
O
N
3ab
^{1}H NMR (400 MHz, CDCl$_3$)
2.00 2.10 3.03 2.08 0.86 1.11 0.93 1.93 3.05 1.01 1.05 2.06
f1 (ppm)

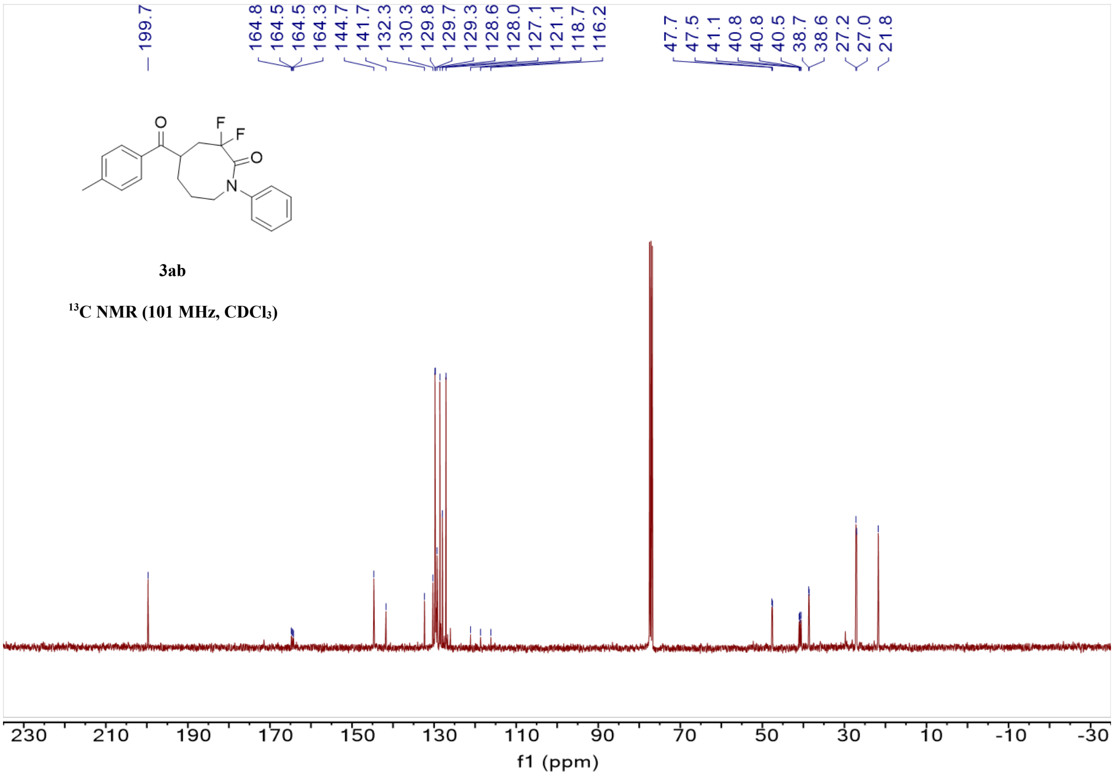
199.7
164.8 164.5 164.5 164.3 144.7 141.7 132.3 130.3 129.8 129.7 129.3 128.6 128.0 127.1 121.1 118.7 116.2
47.7 47.5 41.1 40.8 40.8 40.5 38.7 38.6 27.2 27.0 21.8
F
F
O
O
N
3ab
^{13}C NMR (101 MHz, CDCl$_3$)
f1 (ppm)

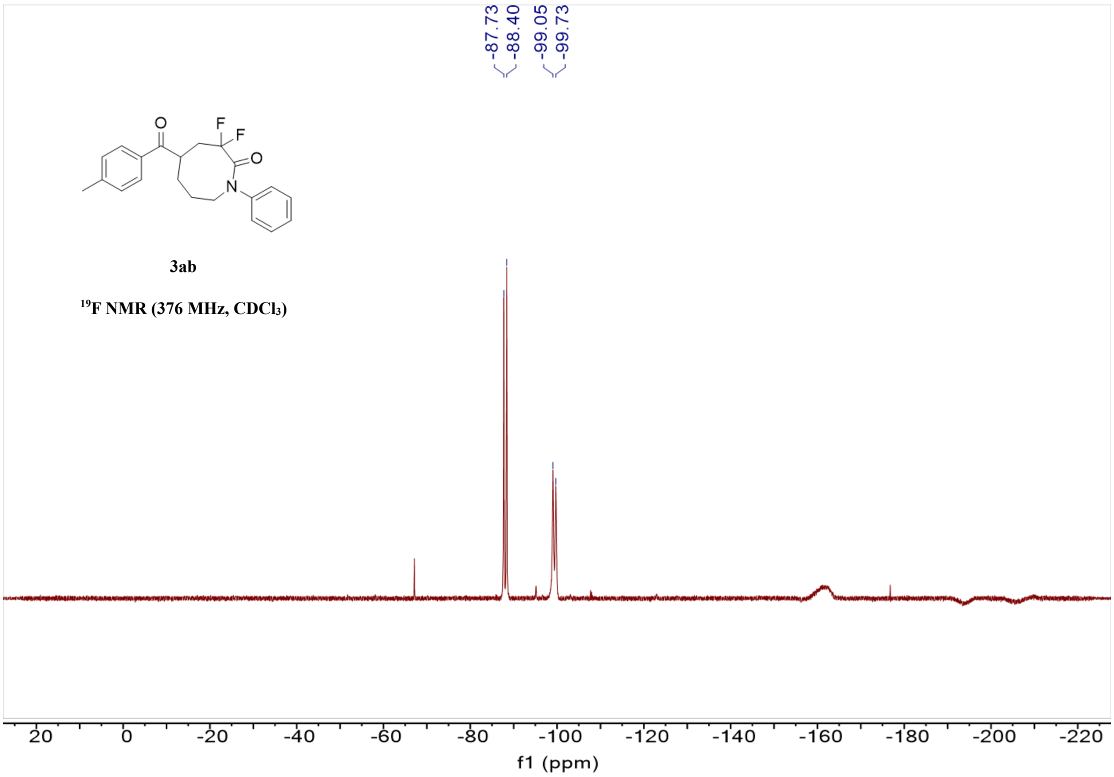
-87.73
-88.40
-99.05
-99.73
3ab
19F NMR (376 MHz, CDCl3)
20 0 -20 -40 -60 -80 -100 -120 -140 -160 -180 -200 -220
f1 (ppm)

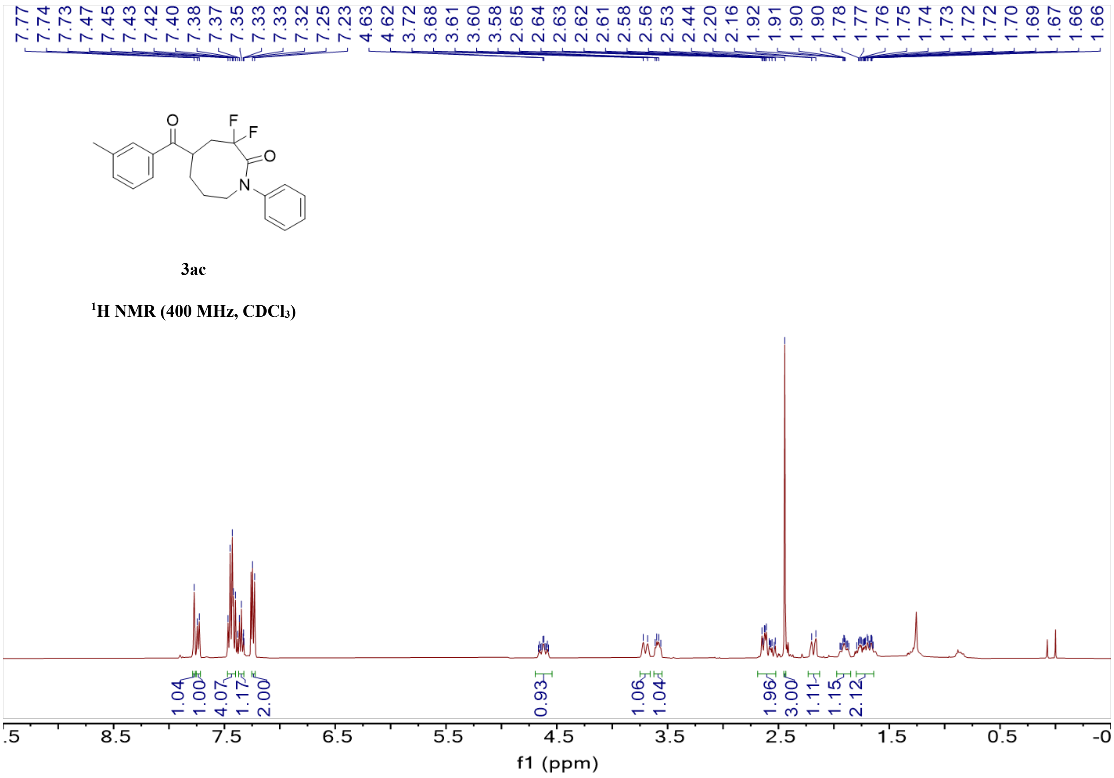
7.77 7.74 7.73 7.47 7.45 7.43 7.42 7.40 7.38 7.37 7.35 7.33 7.33 7.32 7.25 7.23 4.63 4.62 3.72 3.68 3.61 3.60 3.58 2.65 2.64 2.63 2.62 2.61 2.58 2.56 2.53 2.44 2.20 2.16 1.92 1.91 1.90 1.78 1.77 1.76 1.75 1.74 1.73 1.72 1.72 1.70 1.69 1.67 1.66 1.66
O
F
F
O
N
3ac
1H NMR (400 MHz, CDCl3)
1.04 1.00 4.07 1.17 2.00 0.93 1.06 1.04 1.96 3.00 1.11 1.15 2.12
.5 8.5 7.5 6.5 5.5 4.5 3.5 2.5 1.5 0.5 -0
f1 (ppm)

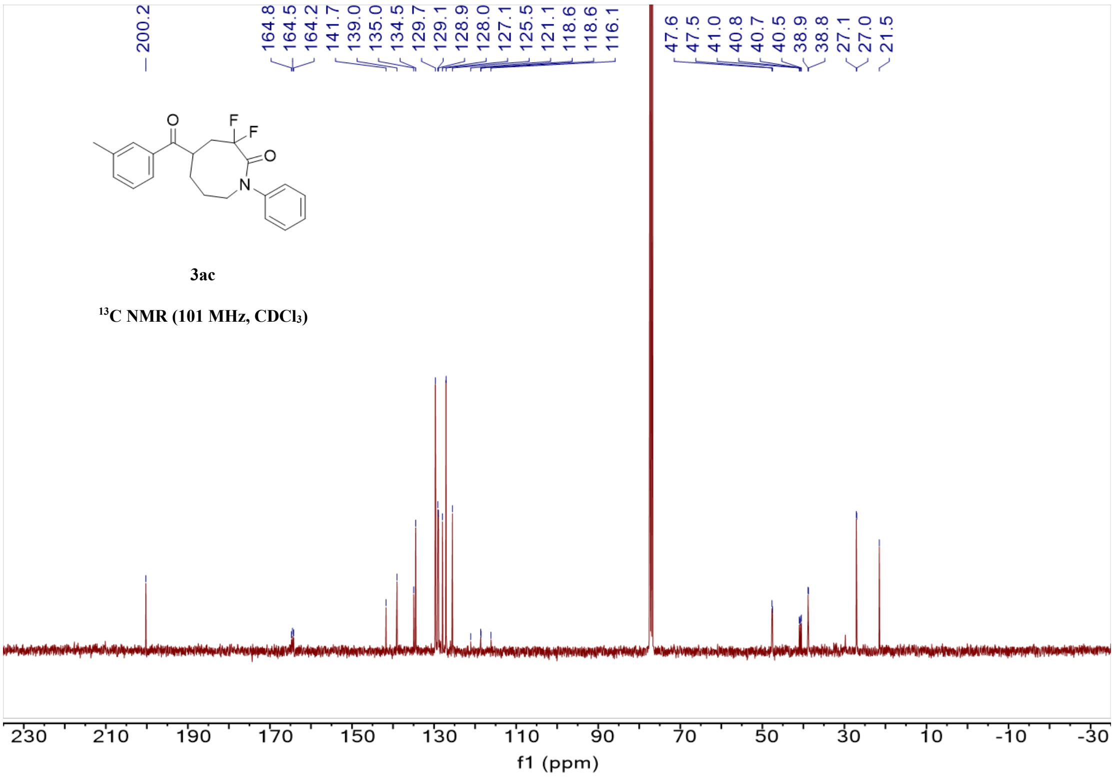
200.2
164.8 164.5 164.2 141.7 139.0 135.0 134.5 129.7 129.1 128.9 128.0 127.1 125.5 121.1 118.6 118.6 116.1
47.6 47.5 41.0 40.8 40.7 40.5 38.9 38.8 27.1 27.0 21.5
O
F
F
O
N
3ac
13C NMR (101 MHz, CDCl3)
230 210 190 170 150 130 110 90 70 50 30 10 -10 -30
f1 (ppm)

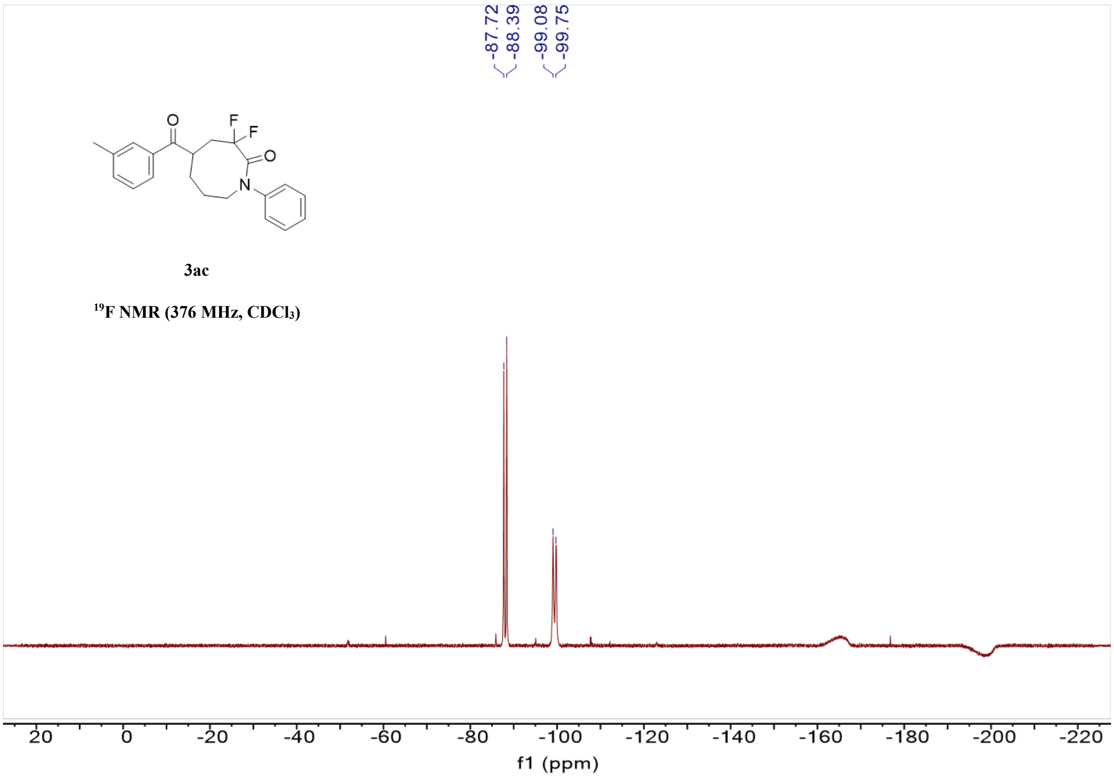

3ac

^{19}F NMR (376 MHz, CDCl$_3$)

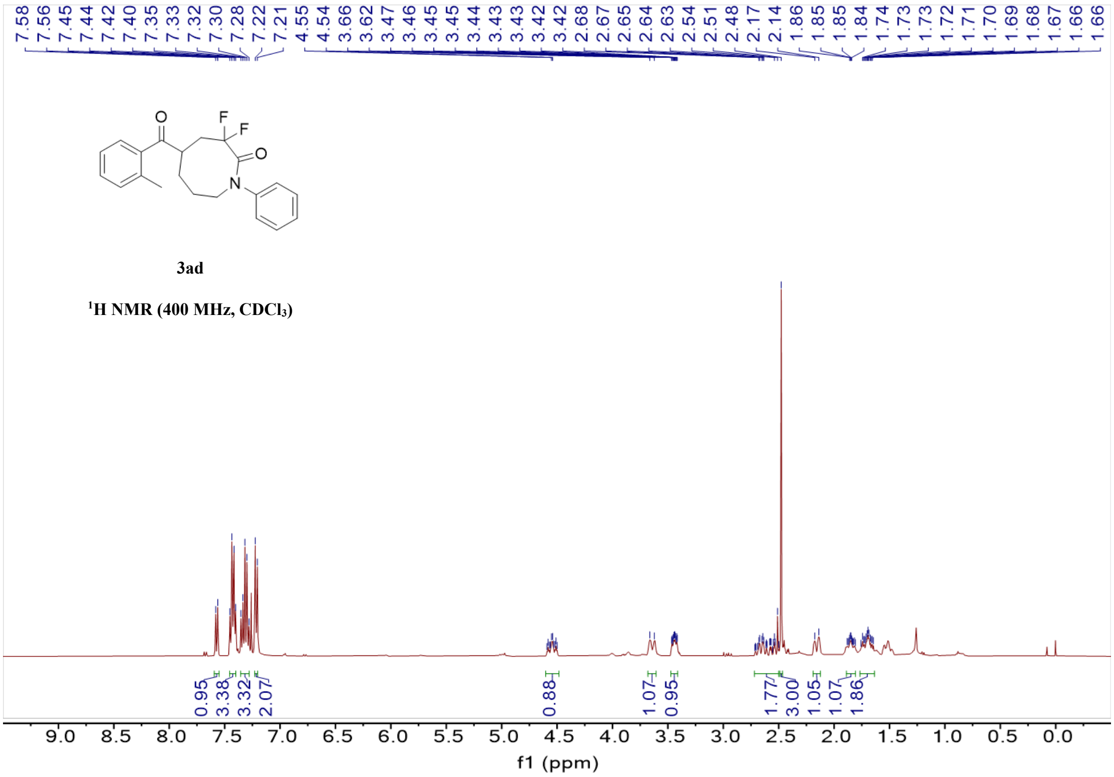
7.58 7.56 7.45 7.44 7.42 7.40 7.35 7.33 7.32 7.30 7.28 7.22 7.21 4.55 4.54 3.66 3.62 3.47 3.46 3.45 3.45 3.44 3.43 3.43 3.42 3.42 2.68 2.67 2.65 2.64 2.63 2.54 2.51 2.48 2.17 2.14 1.86 1.85 1.85 1.84 1.74 1.73 1.73 1.72 1.71 1.70 1.69 1.68 1.67 1.66 1.66
O
F
F
O
N
3ad
^{1}H NMR (400 MHz, CDCl$_3$)
0.95 3.38 3.32 2.07 0.88 1.07 0.95 1.77 3.00 1.05 1.07 1.86
9.0 8.5 8.0 7.5 7.0 6.5 6.0 5.5 5.0 4.5 4.0 3.5 3.0 2.5 2.0 1.5 1.0 0.5 0.0
f1 (ppm)

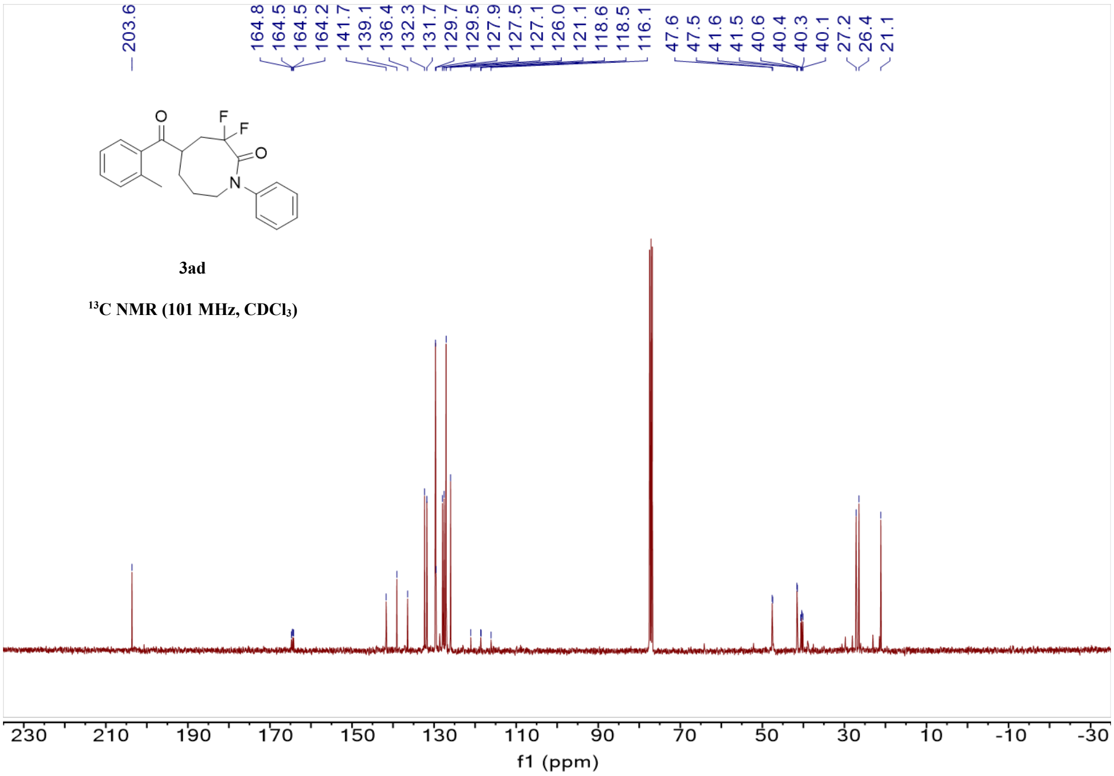
203.6
164.8 164.5 164.5 164.2 141.7 139.1 136.4 132.3 131.7 129.7 129.5 127.9 127.5 127.1 126.0 121.1 118.6 118.5 116.1
47.6 47.5 41.6 41.5 40.6 40.4 40.3 40.1 27.2 26.4 21.1
O
F
F
O
N
3ad
^{13}C NMR (101 MHz, CDCl$_3$)
230 210 190 170 150 130 110 90 70 50 30 10 -10 -30
f1 (ppm)

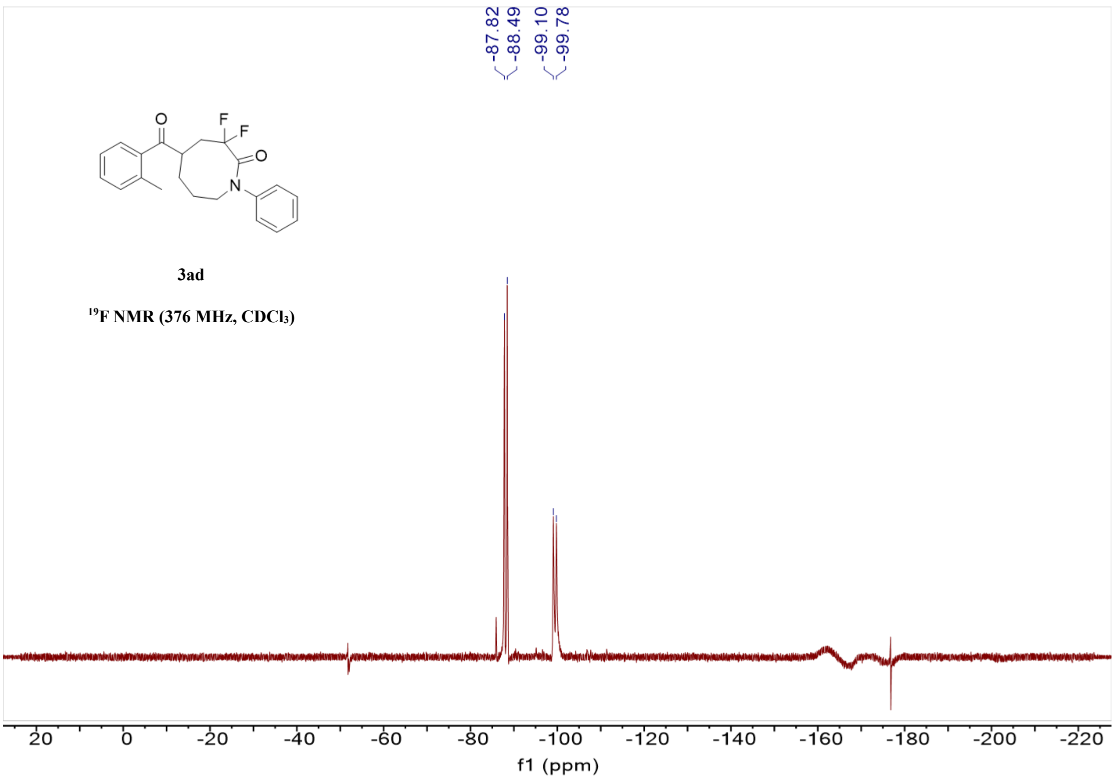
-87.82
-88.49
-99.10
-99.78
O
F
F
O
N
3ad
^{19}F NMR (376 MHz, CDCl$_3$)
20
0
-20
-40
-60
-80
-100
-120
-140
-160
-180
-200
-220
f1 (ppm)

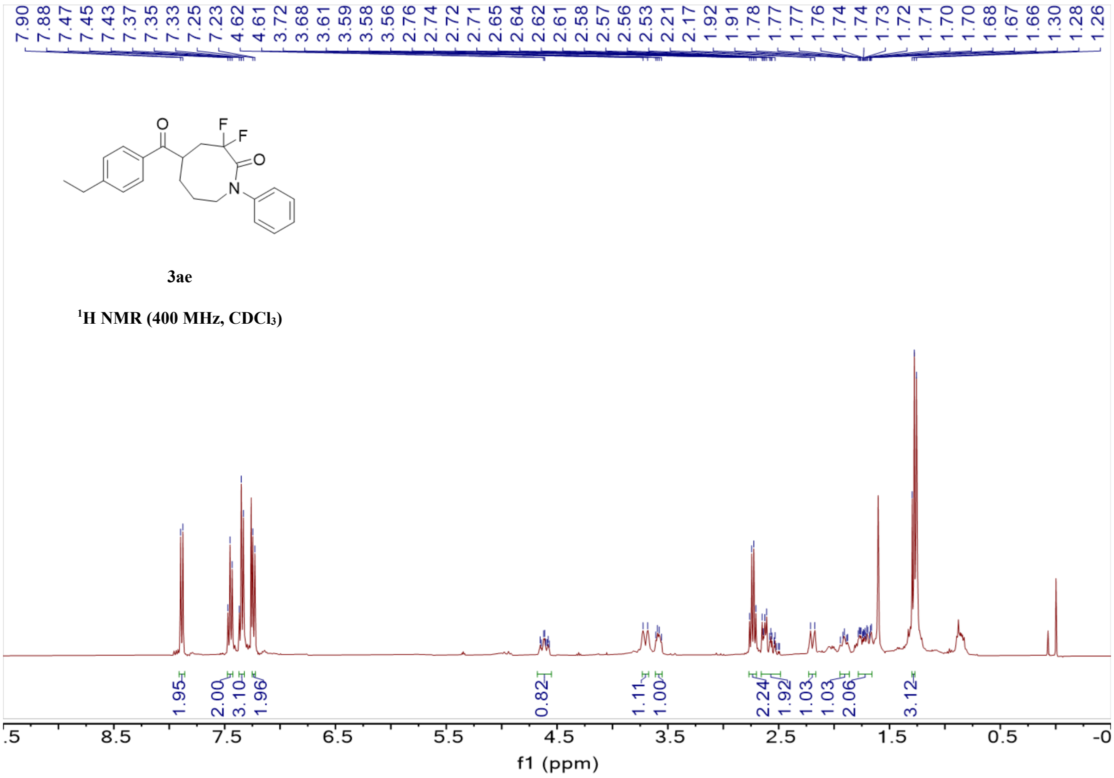

3ae

^{1}H NMR (400 MHz, CDCl$_3$)

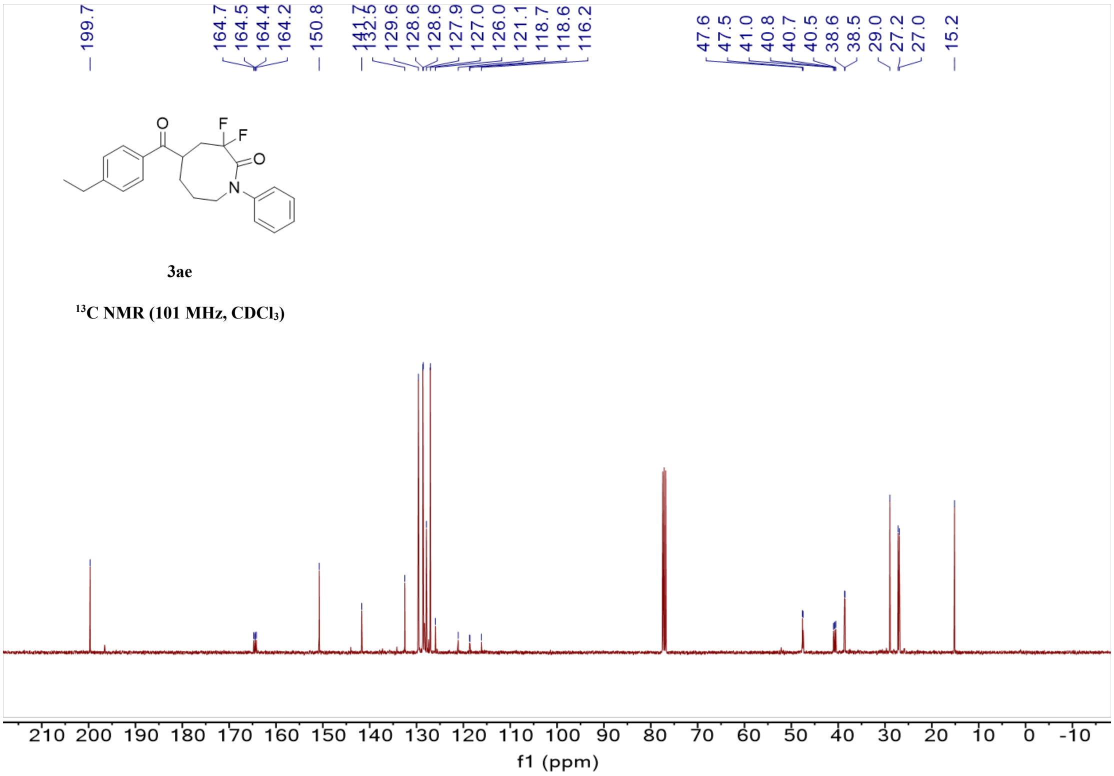

3ae

^{13}C NMR (101 MHz, CDCl$_3$)

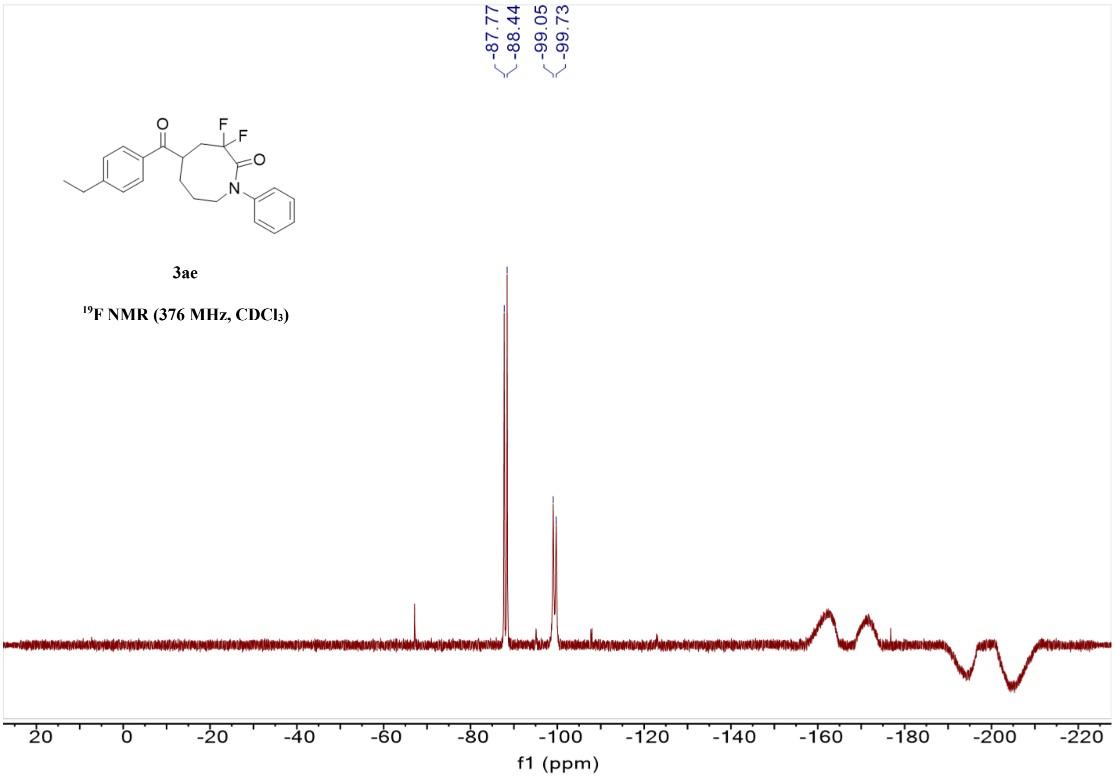
-87.77
-88.44
-99.05
-99.73
3ae
^{19}F NMR (376 MHz, CDCl$_3$)
20 0 -20 -40 -60 -80 -100 -120 -140 -160 -180 -200 -220
f1 (ppm)

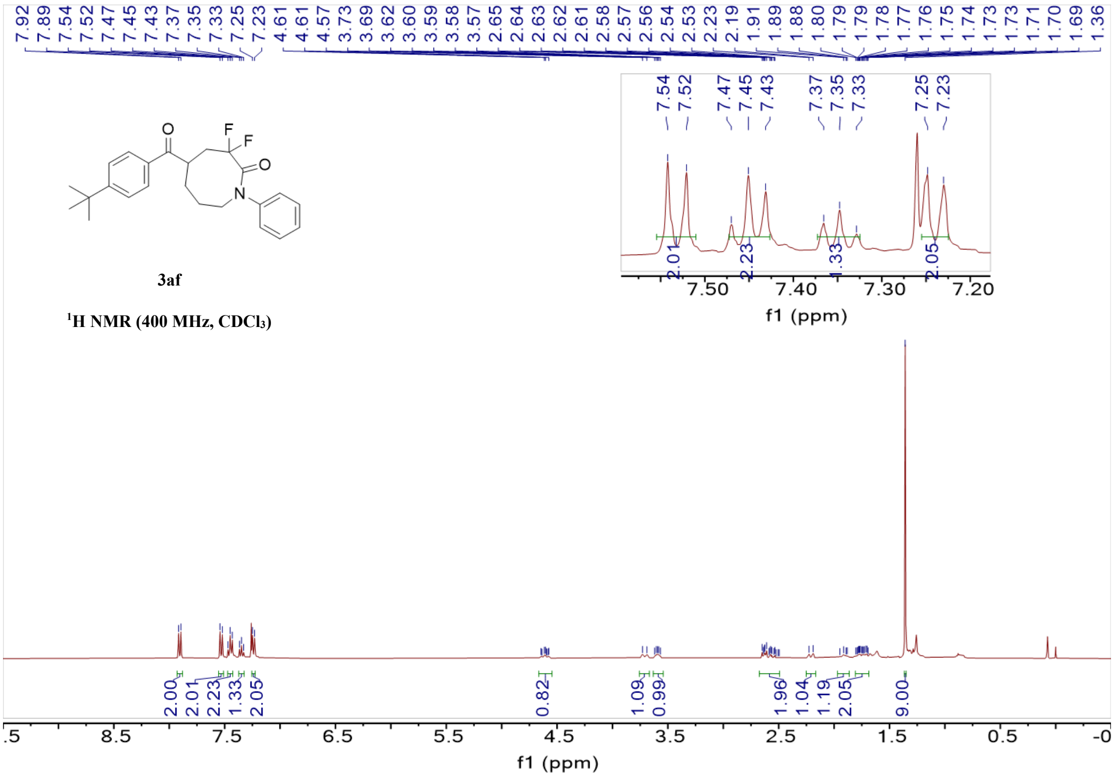

7.92 7.89 7.54 7.52 7.47 7.45 7.43 7.37 7.35 7.33 7.25 7.23 4.61 4.57 3.73 3.69 3.62 3.60 3.59 3.58 3.57 2.65 2.64 2.63 2.62 2.61 2.58 2.57 2.56 2.54 2.53 2.23 2.19 1.91 1.89 1.88 1.80 1.79 1.79 1.78 1.77 1.76 1.75 1.74 1.73 1.73 1.71 1.70 1.69 1.36
3af
^{1}H NMR (400 MHz, CDCl$_3$)
f1 (ppm)

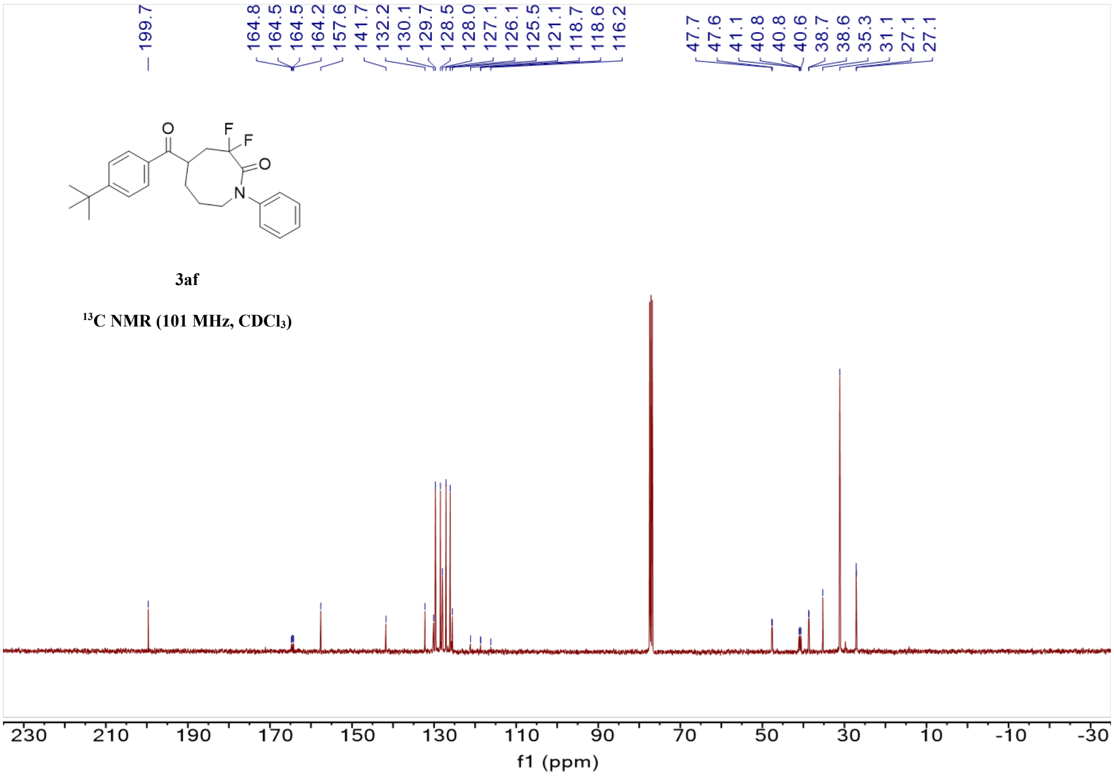

199.7 164.8 164.5 164.5 164.2 157.6 141.7 132.2 130.1 129.7 128.5 128.0 127.1 126.1 125.5 121.1 118.7 118.6 116.2 47.7 47.6 41.1 40.8 40.8 40.6 38.7 38.6 35.3 31.1 27.1 27.1
3af
^{13}C NMR (101 MHz, CDCl$_3$)
f1 (ppm)

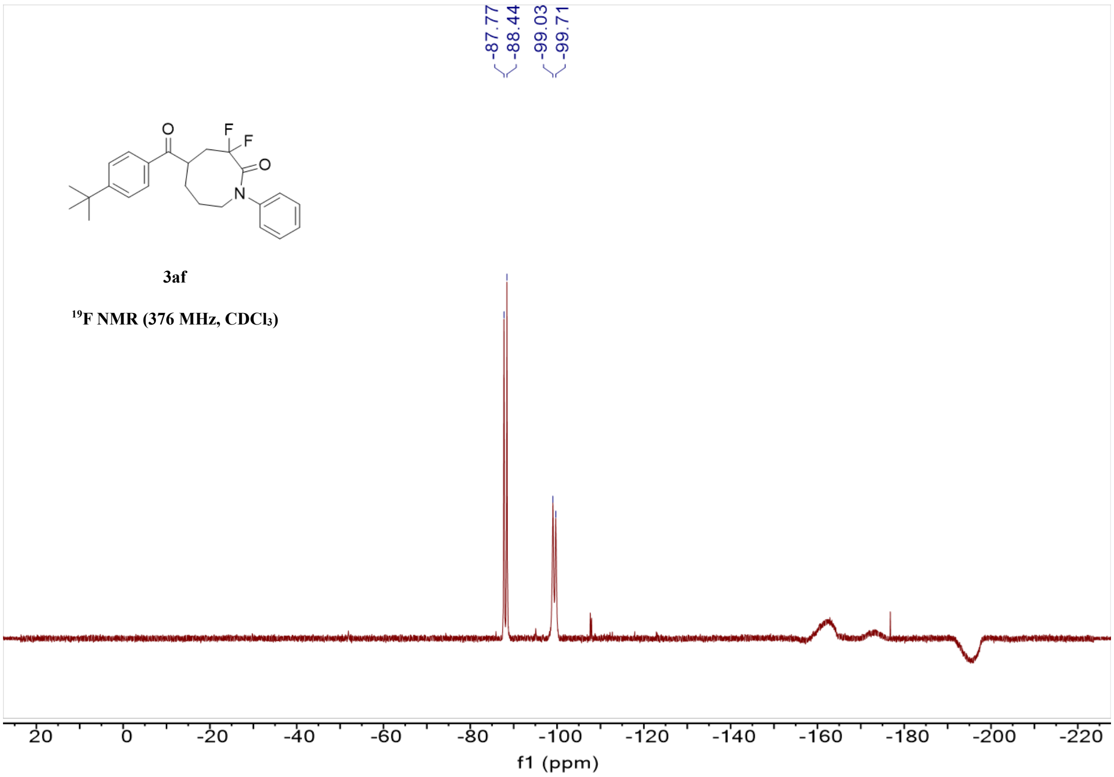
-87.77
-88.44
-99.03
-99.71
3af
19F NMR (376 MHz, CDCl3)
20
0
-20
-40
-60
-80
-100
-120
-140
-160
-180
-200
-220
f1 (ppm)

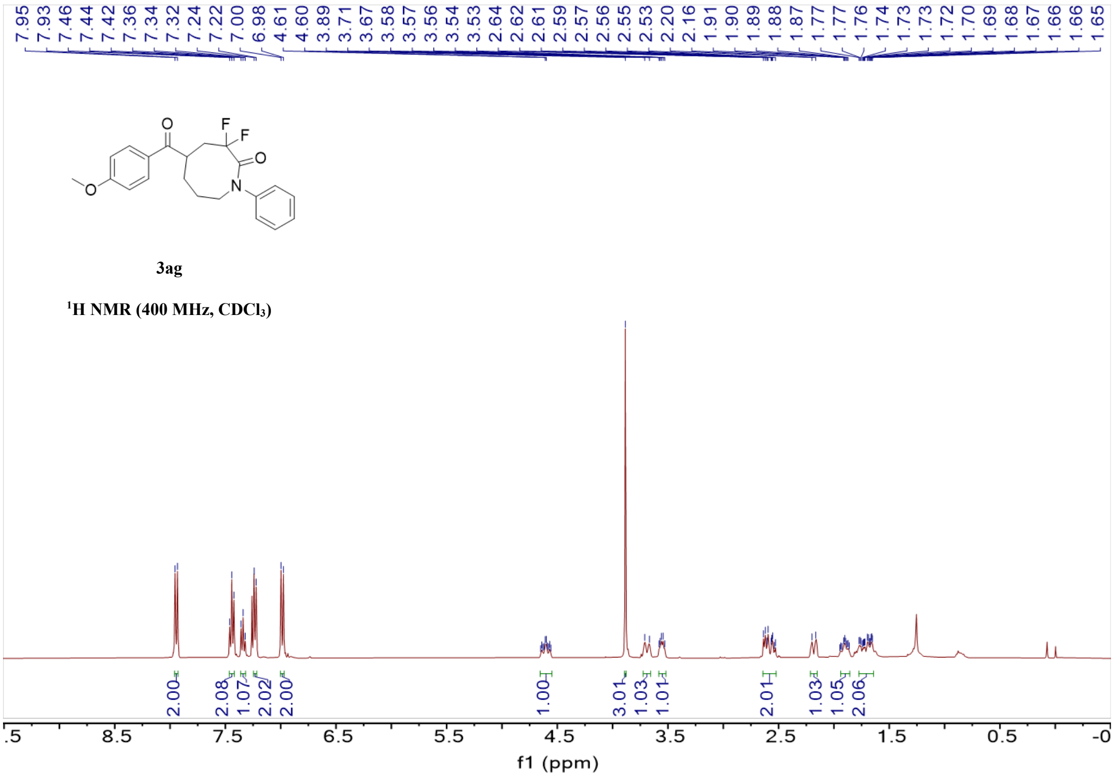
7.95 7.93 7.46 7.44 7.42 7.36 7.34 7.32 7.24 7.22 7.00 6.98 4.61 4.60 3.89 3.71 3.67 3.58 3.57 3.56 3.54 3.53 2.64 2.62 2.61 2.59 2.57 2.56 2.55 2.53 2.20 2.16 1.91 1.90 1.89 1.88 1.87 1.77 1.77 1.76 1.74 1.73 1.73 1.72 1.70 1.69 1.68 1.67 1.66 1.66 1.65
O
F
F
O
O
N
3ag
^{1}H NMR (400 MHz, CDCl3)
2.00 2.08 1.07 2.02 2.00 1.00 3.01 1.03 1.01 2.01 1.03 1.05 2.06
.5 8.5 7.5 6.5 5.5 4.5 3.5 2.5 1.5 0.5 -0
f1 (ppm)

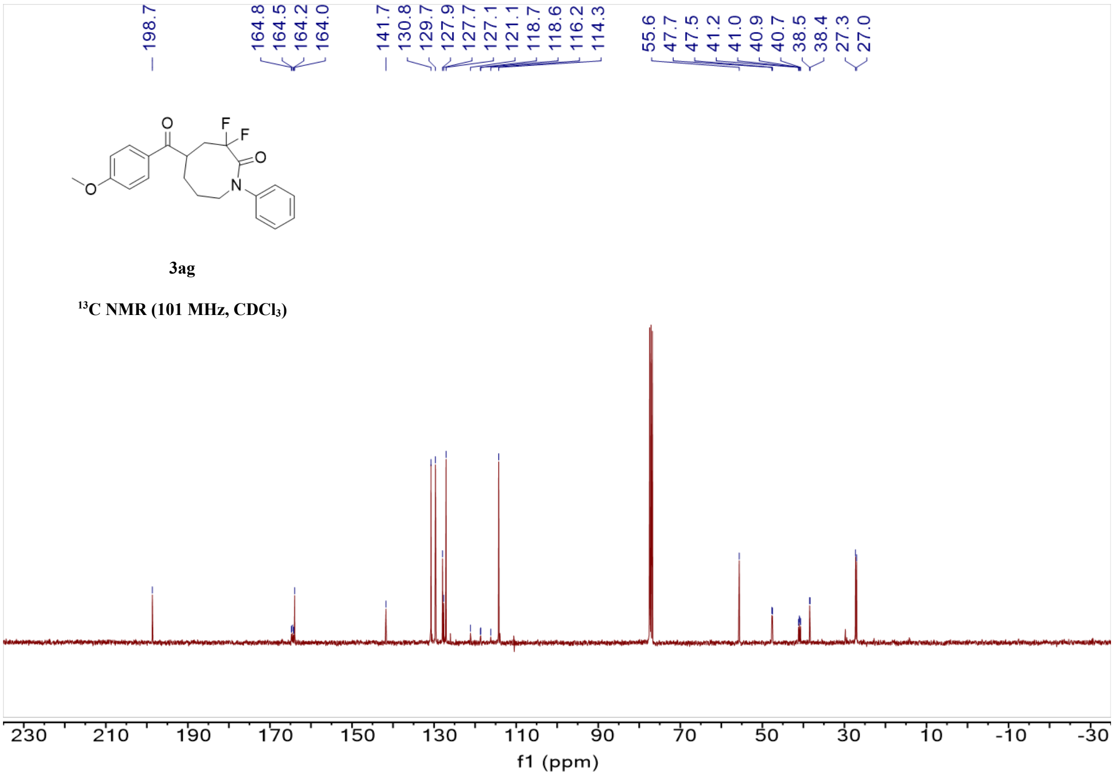
198.7
164.8 164.5 164.2 164.0
141.7 130.8 129.7 127.9 127.7 127.1 121.1 118.7 118.6 116.2 114.3
55.6 47.7 47.5 41.2 41.0 40.9 40.7 38.5 38.4 27.3 27.0
O
F
F
O
O
N
3ag
^{13}C NMR (101 MHz, CDCl3)
230 210 190 170 150 130 110 90 70 50 30 10 -10 -30
f1 (ppm)

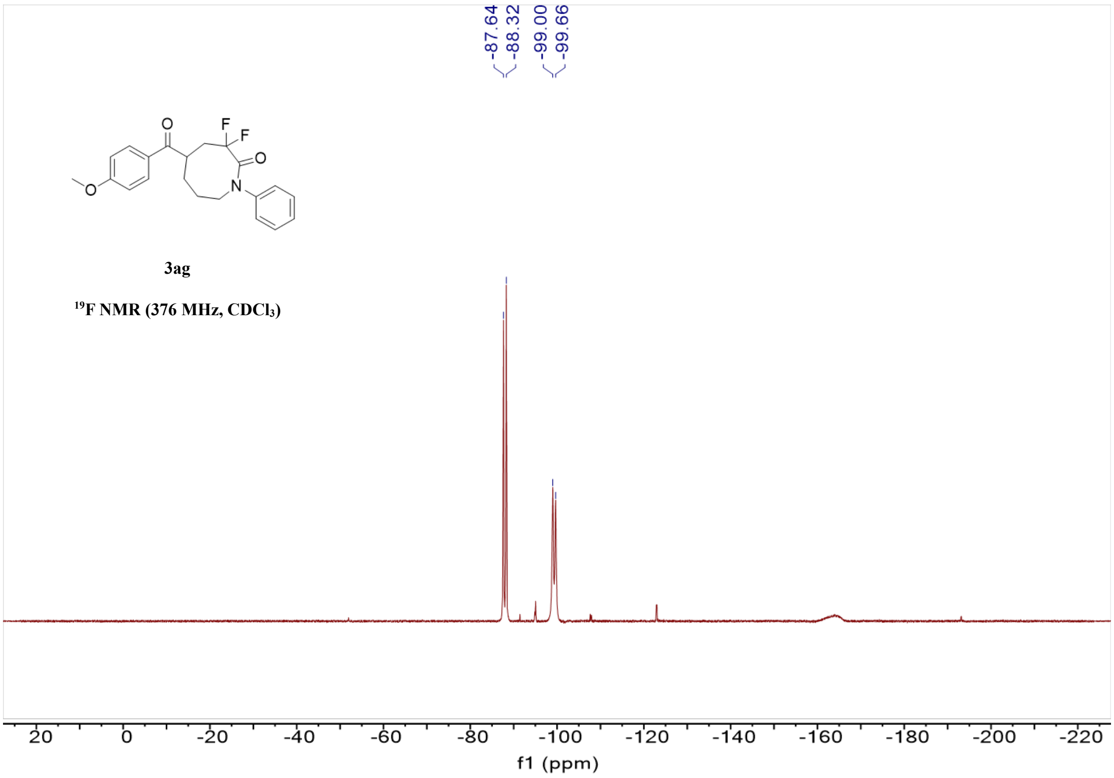

-87.64
-88.32
-99.00
-99.66
O
F
F
O
O
N
3ag
19F NMR (376 MHz, CDCl3)
20 0 -20 -40 -60 -80 -100 -120 -140 -160 -180 -200 -220
f1 (ppm)

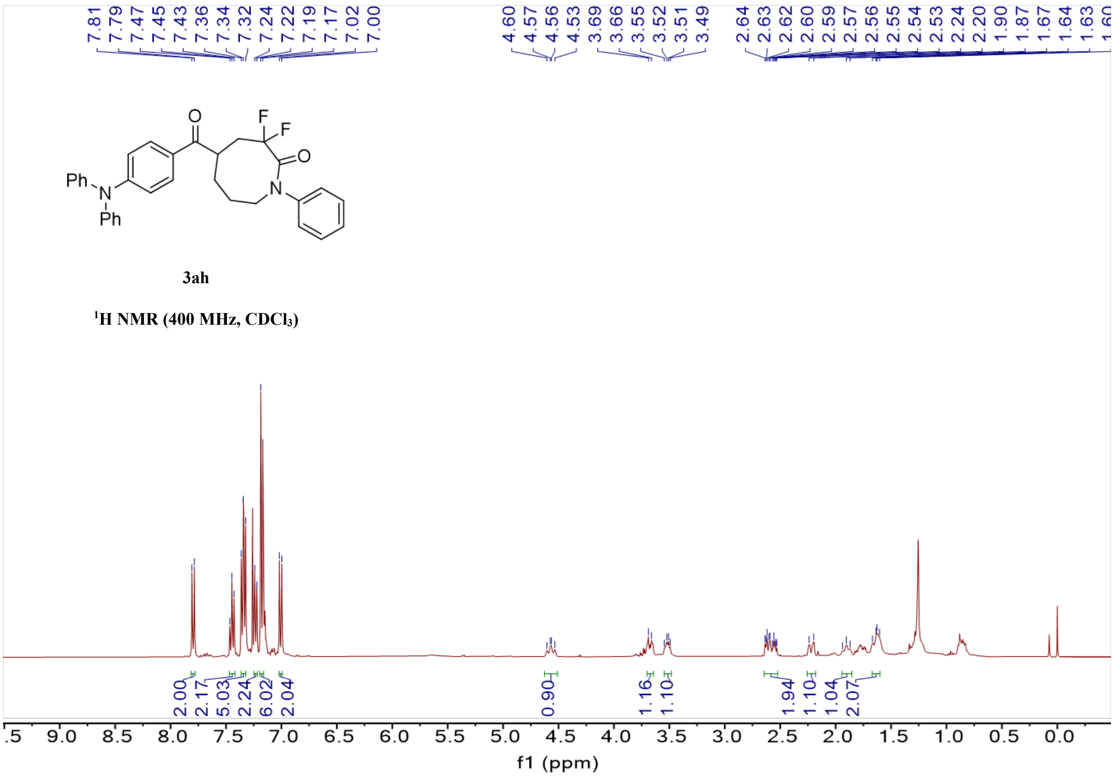
7.81 7.79 7.47 7.45 7.43 7.36 7.34 7.32 7.24 7.22 7.19 7.17 7.02 7.00
4.60 4.57 4.56 4.53 3.69 3.66 3.55 3.52 3.51 3.49
2.64 2.63 2.62 2.60 2.59 2.57 2.56 2.55 2.54 2.53 2.24 2.20 1.90 1.87 1.67 1.64 1.63
3ah
^{1}H NMR (400 MHz, CDCl$_3$)
2.00 2.17 5.03 2.24 6.02 2.04 0.90 1.16 1.10 1.94 1.10 1.04 2.07
f1 (ppm)

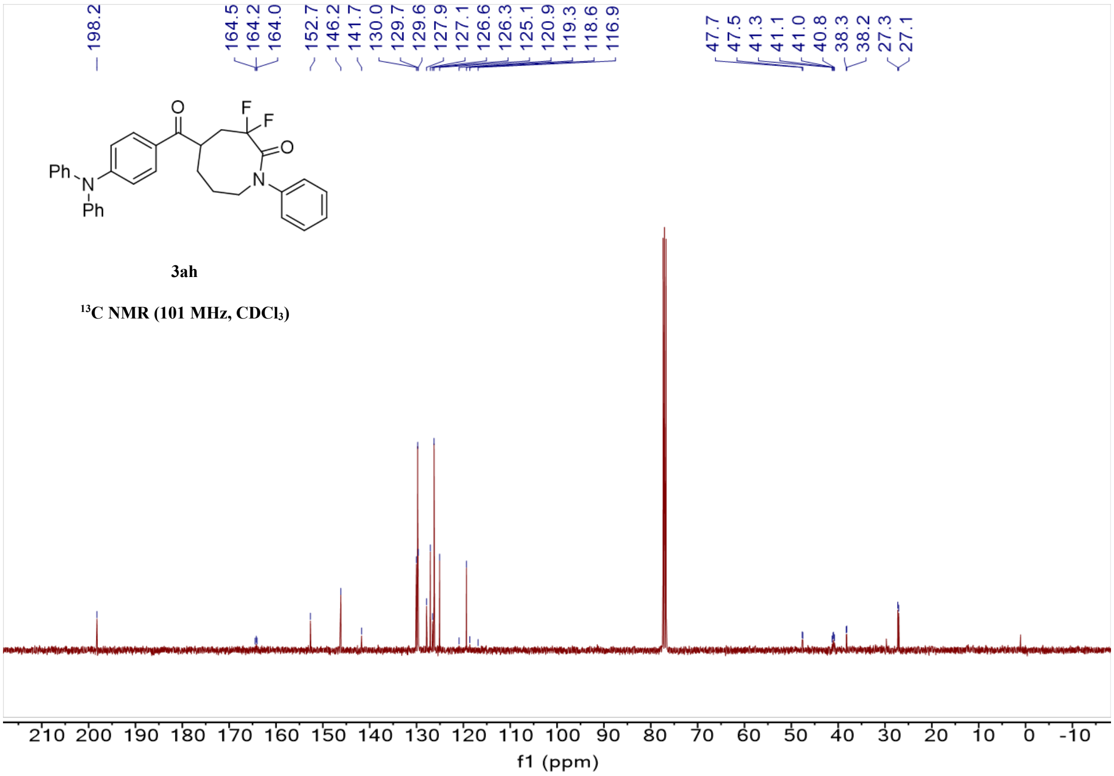
198.2
164.5 164.2 164.0 152.7 146.2 141.7 130.0 129.7 129.6 127.9 127.1 126.6 126.3 125.1 120.9 119.3 118.6 116.9
47.7 47.5 41.3 41.1 41.0 40.8 38.3 38.2 27.3 27.1
3ah
^{13}C NMR (101 MHz, CDCl$_3$)
f1 (ppm)

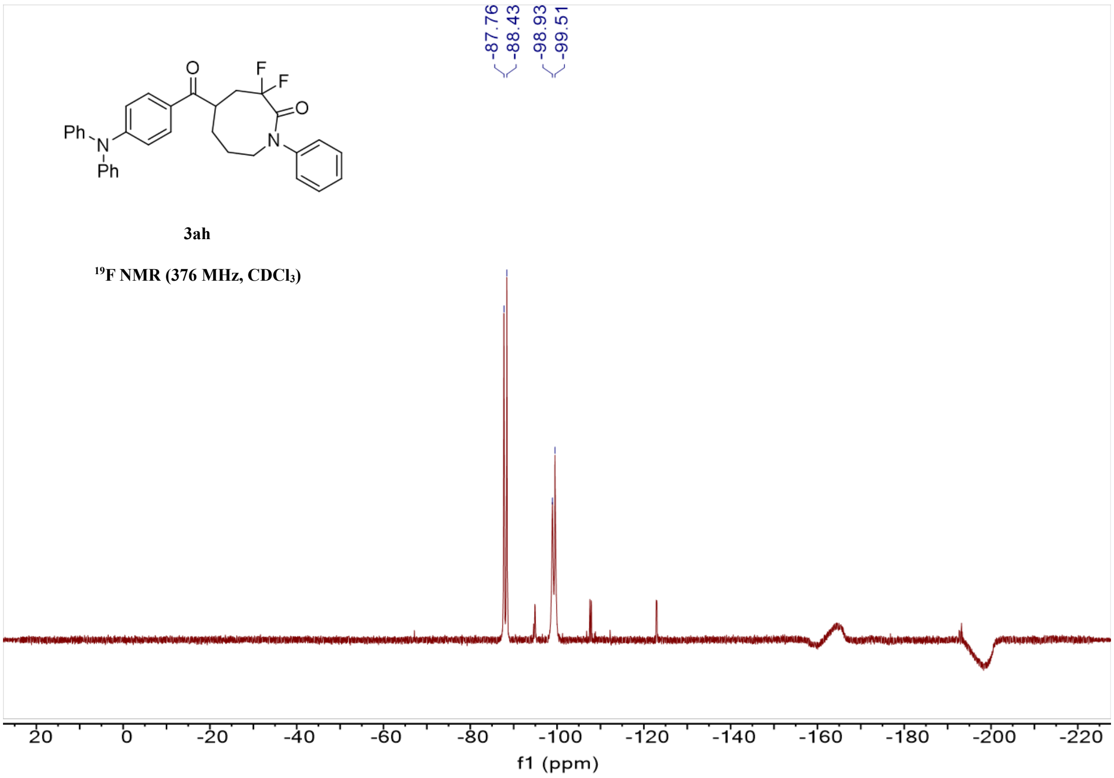

3ah

^{19}F NMR (376 MHz, CDCl$_3$)

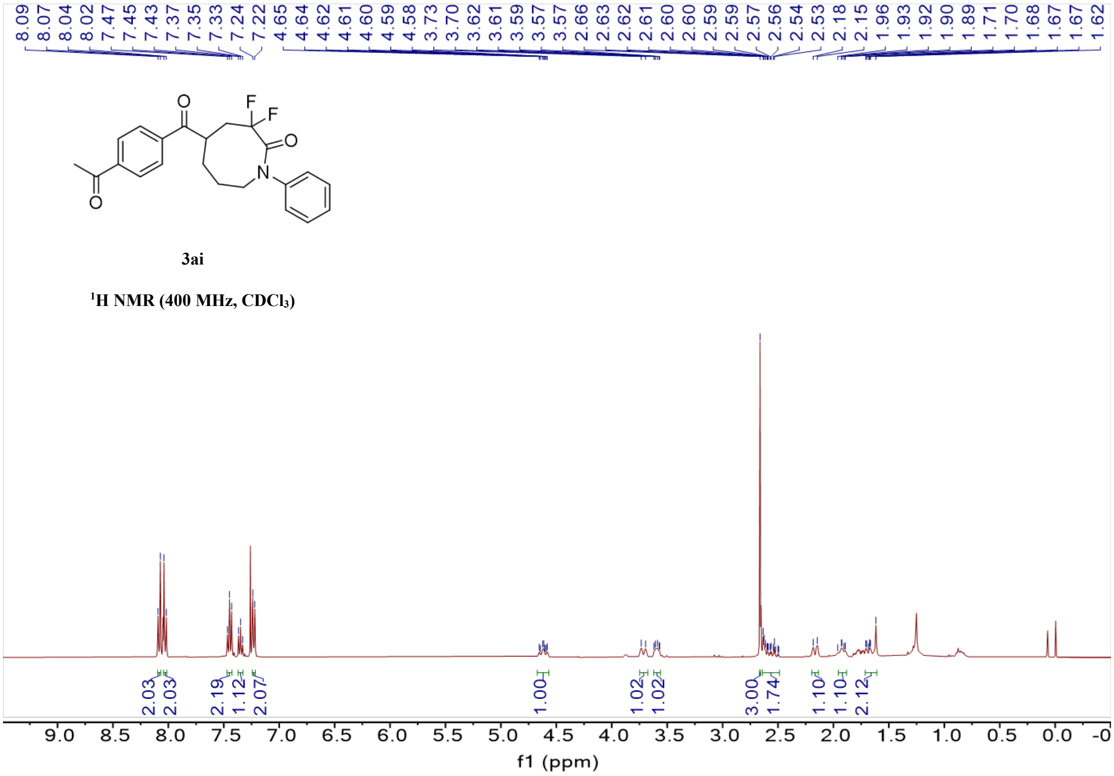

3ai

^{1}H NMR (400 MHz, $CDCl_3$)

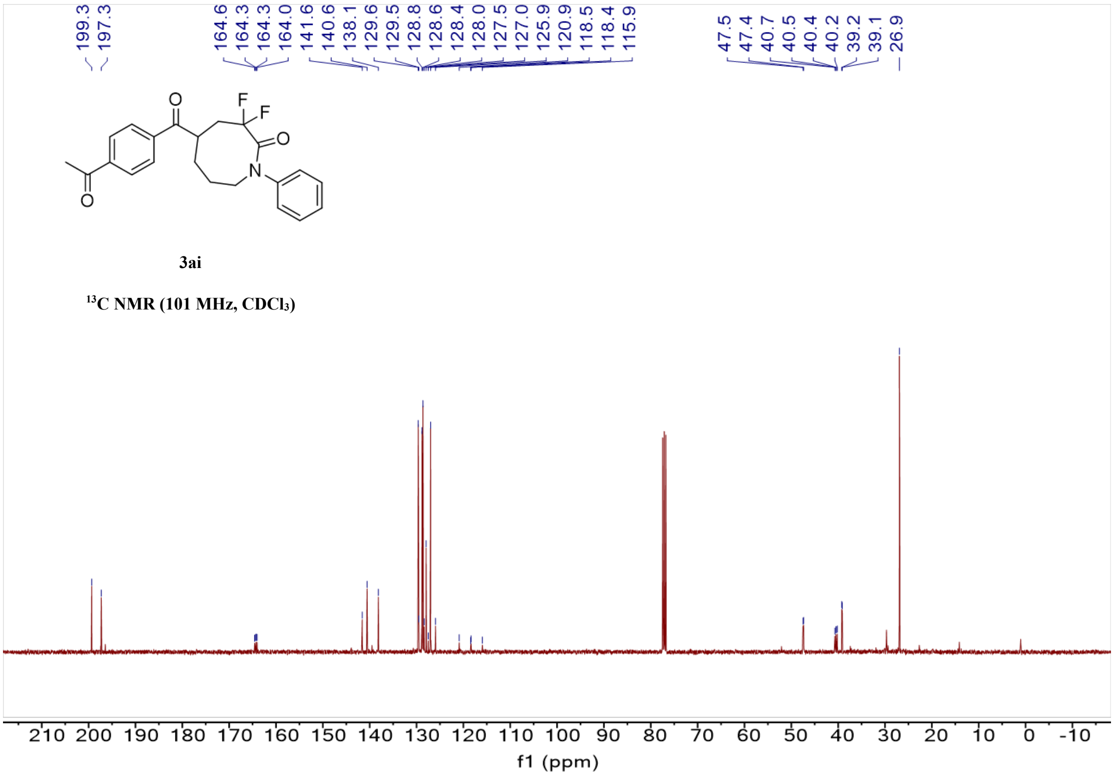

3ai

^{13}C NMR (101 MHz, $CDCl_3$)

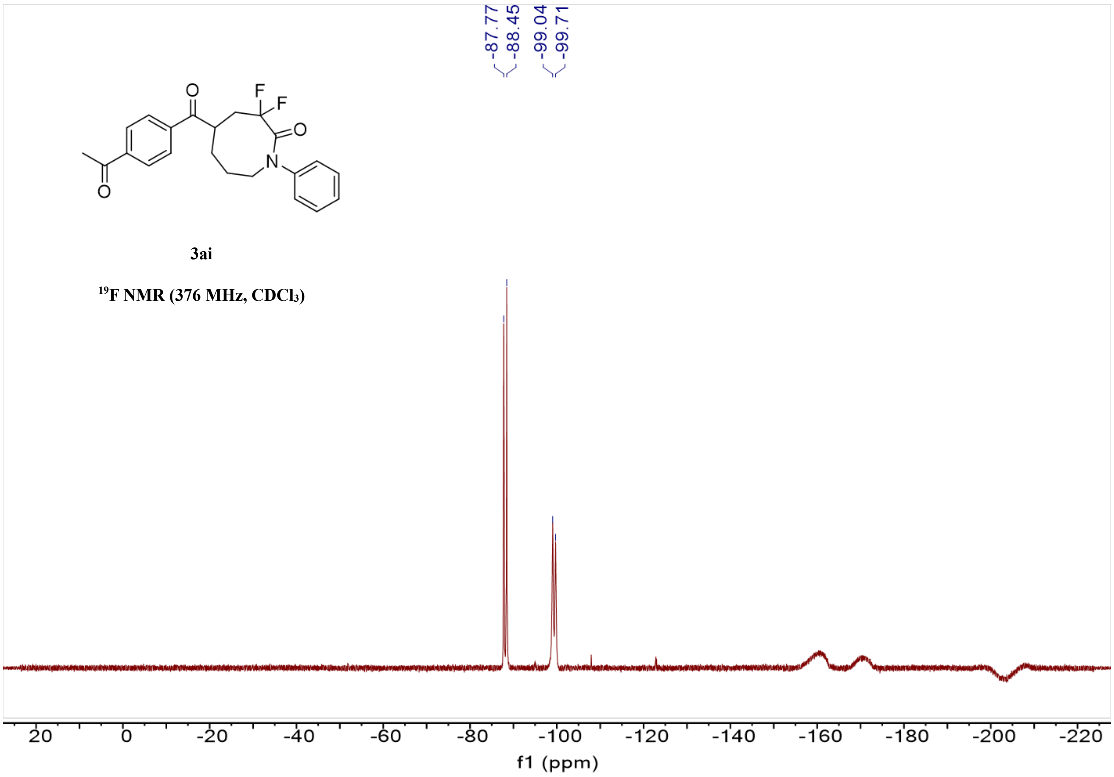

-87.77
-88.45
-99.04
-99.71
3ai
19F NMR (376 MHz, CDCl3)
20
0
-20
-40
-60
-80
-100
-120
-140
-160
-180
-200
-220
f1 (ppm)

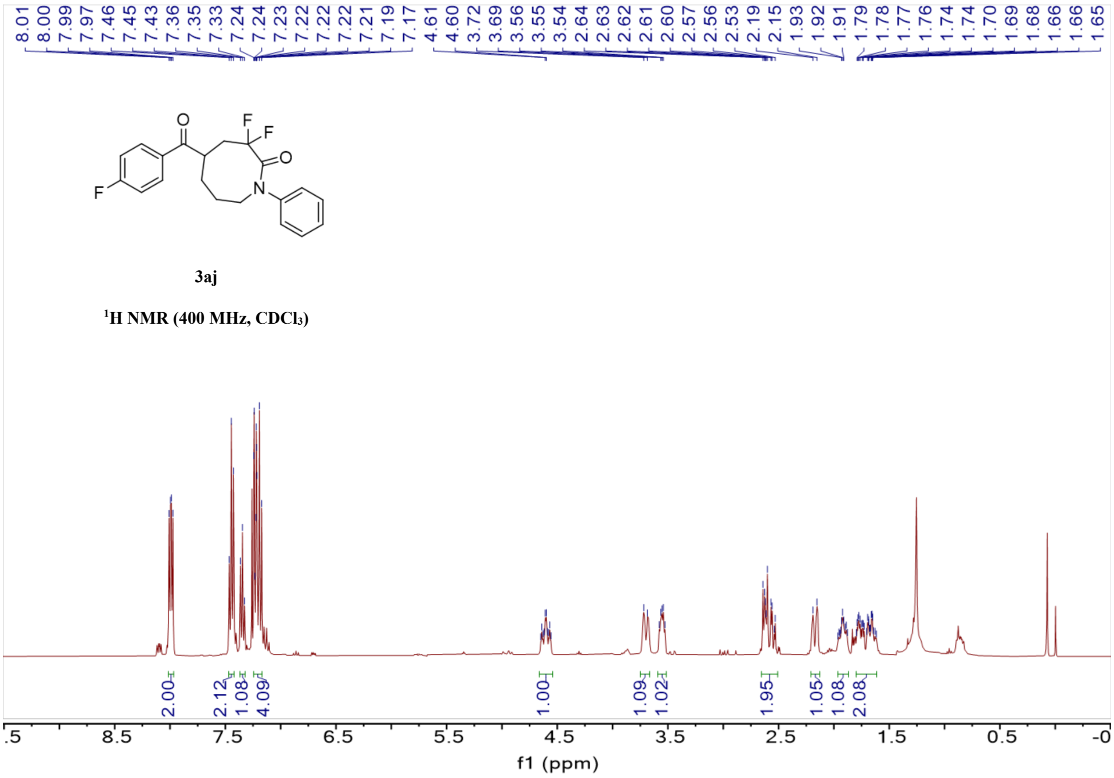
8.01 8.00 7.99 7.97 7.46 7.45 7.43 7.36 7.35 7.33 7.24 7.24 7.23 7.22 7.22 7.22 7.21 7.19 7.17 4.61 4.60 3.72 3.69 3.56 3.55 3.54 2.64 2.63 2.62 2.61 2.60 2.57 2.56 2.53 2.19 2.15 1.93 1.92 1.91 1.79 1.78 1.77 1.76 1.74 1.70 1.69 1.68 1.66 1.65
3aj
^{1}H NMR (400 MHz, CDCl$_3$)
2.00 2.12 1.08 4.09 1.00 1.09 1.02 1.95 1.05 1.08 2.08
f1 (ppm)

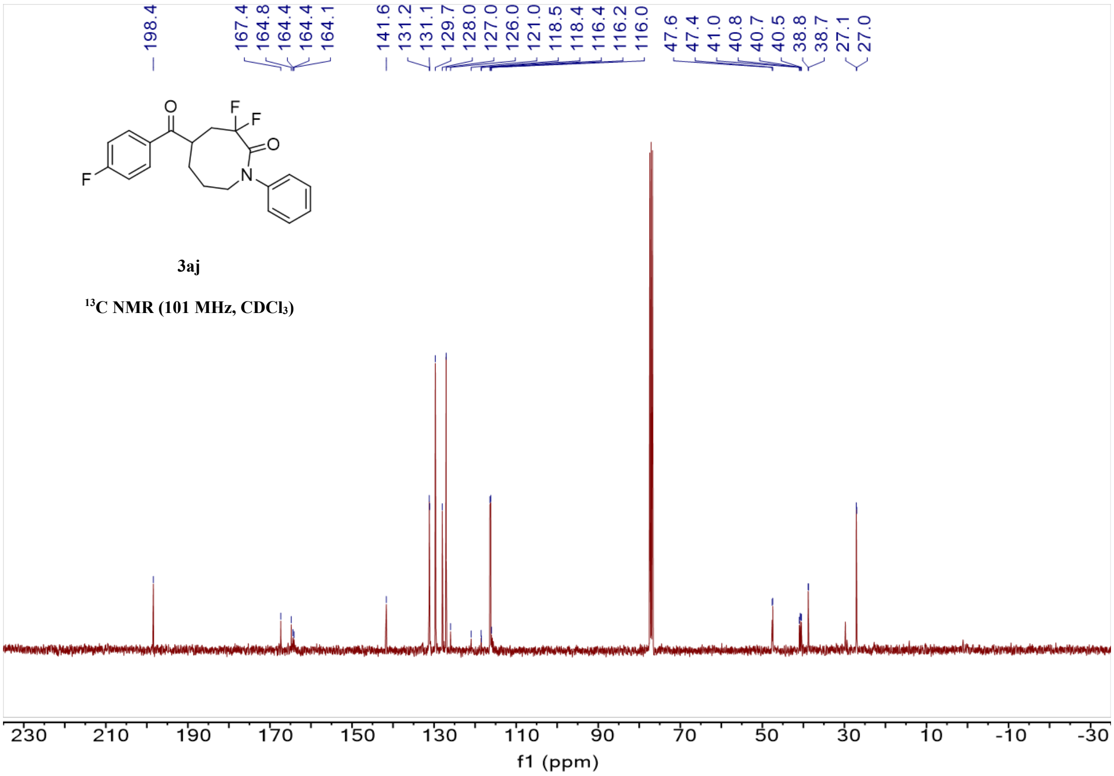
198.4 167.4 164.8 164.4 164.4 164.1 141.6 131.2 131.1 129.7 128.0 127.0 126.0 121.0 118.5 118.4 116.4 116.2 116.0 47.6 47.4 41.0 40.8 40.7 40.5 38.8 38.7 27.1 27.0
3aj
^{13}C NMR (101 MHz, CDCl$_3$)
f1 (ppm)

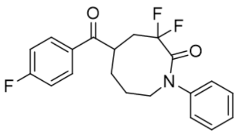

3aj

^{19}F NMR (376 MHz, CDCl$_3$)

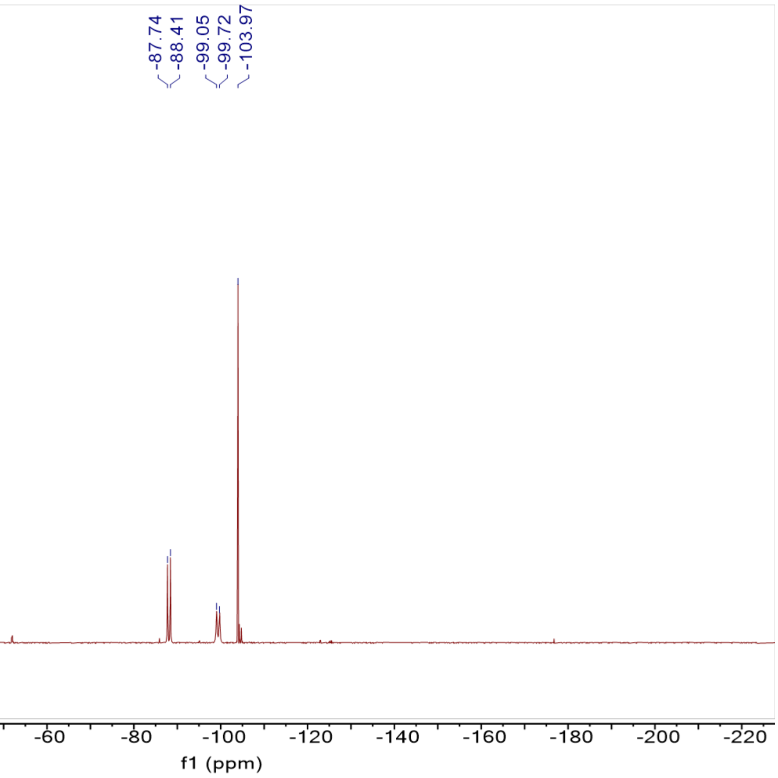

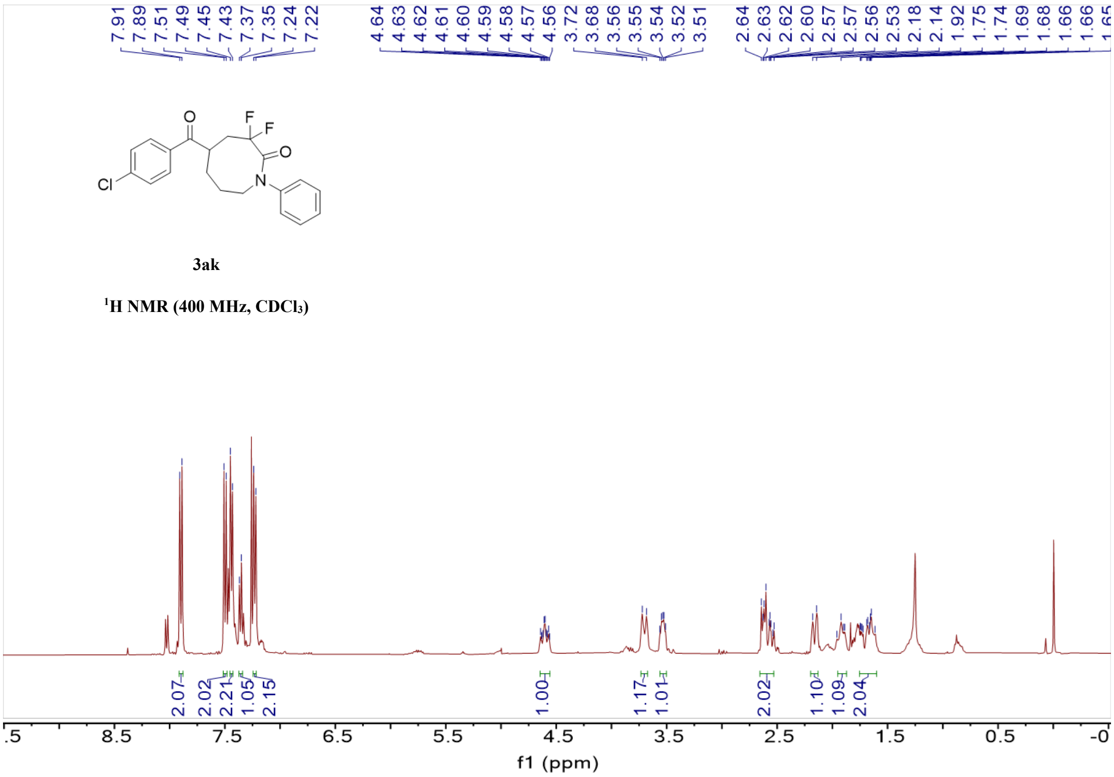
7.91 7.89 7.51 7.49 7.45 7.43 7.37 7.35 7.24 7.22 4.64 4.63 4.62 4.61 4.60 4.59 4.58 4.57 4.56 3.72 3.68 3.56 3.55 3.54 3.52 3.51 2.64 2.63 2.62 2.60 2.57 2.57 2.56 2.53 2.18 2.14 1.92 1.75 1.74 1.69 1.68 1.66 1.66
O
F
F
O
Cl
N
3ak
1H NMR (400 MHz, CDCl3)
2.07 2.02 2.21 1.05 2.15 1.00 1.17 1.01 2.02 1.10 1.09 2.04
.5 8.5 7.5 6.5 5.5 4.5 3.5 2.5 1.5 0.5 -0
f1 (ppm)

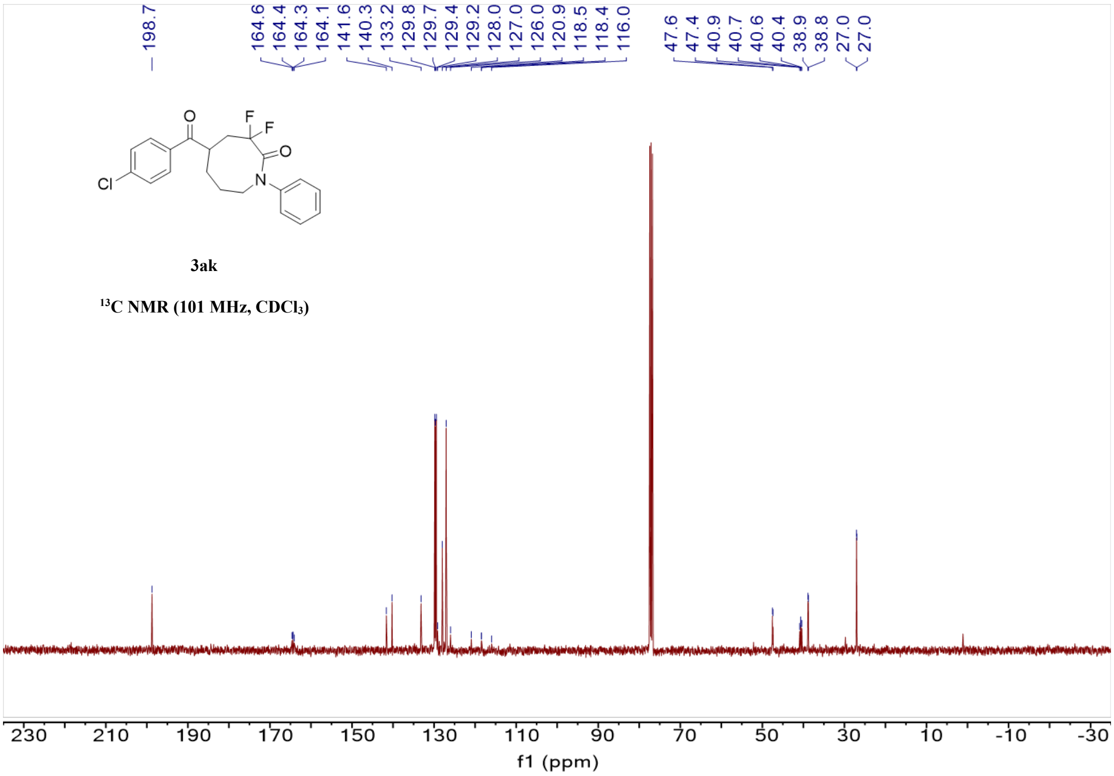
198.7 164.6 164.4 164.3 164.1 141.6 140.3 133.2 129.8 129.7 129.4 129.2 128.0 127.0 126.0 120.9 118.5 118.4 116.0 47.6 47.4 40.9 40.7 40.6 40.4 38.9 38.8 27.0 27.0
O
F
F
O
Cl
N
3ak
13C NMR (101 MHz, CDCl3)
230 210 190 170 150 130 110 90 70 50 30 10 -10 -30
f1 (ppm)

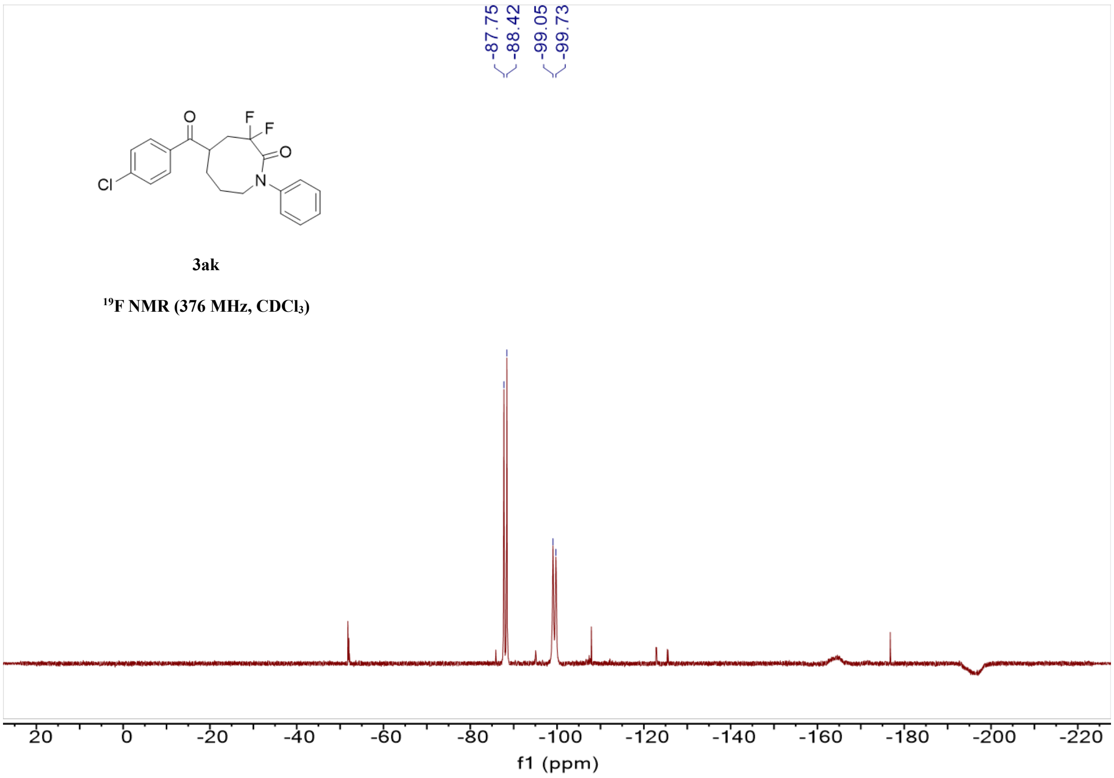

3ak

^{19}F NMR (376 MHz, $CDCl_3$)

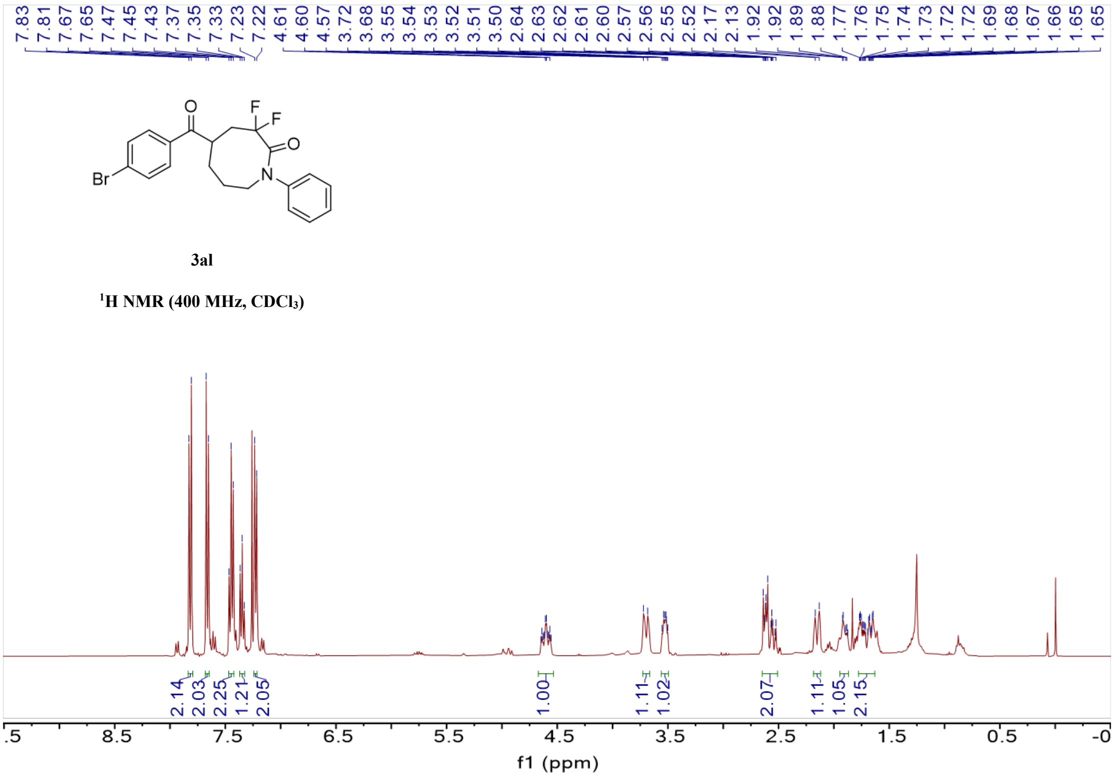
3al
^{1}H NMR (400 MHz, $CDCl_3$)
f1 (ppm)

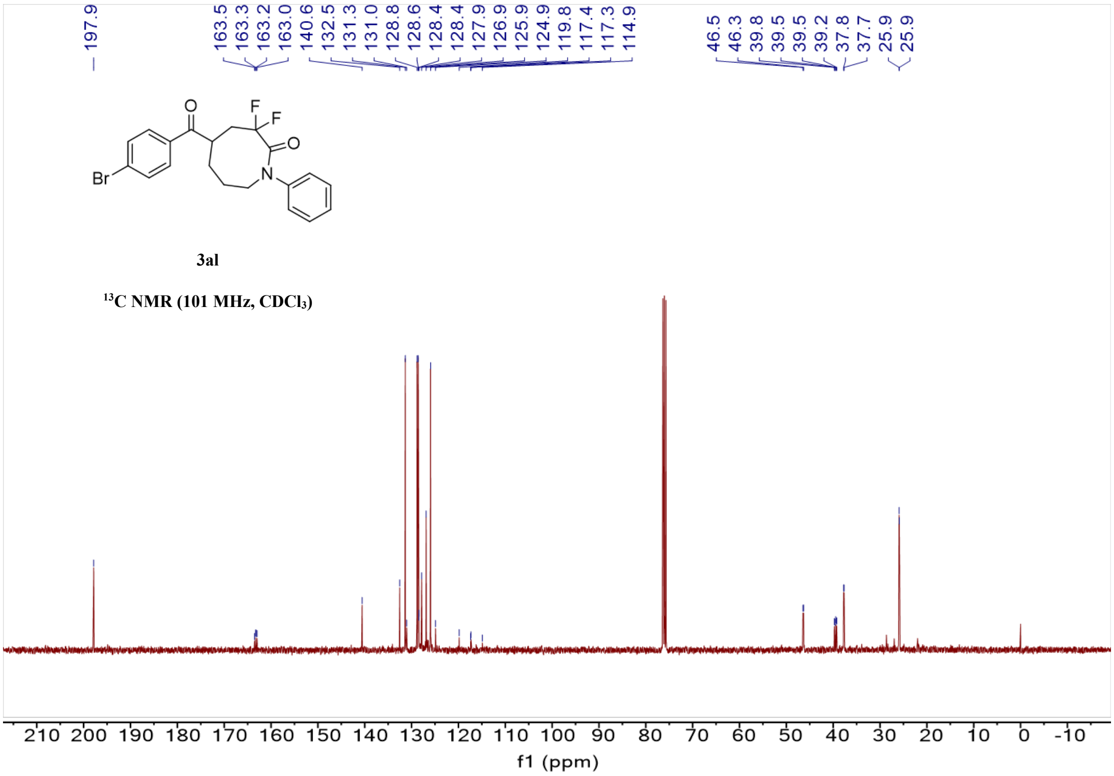
3al
^{13}C NMR (101 MHz, $CDCl_3$)
f1 (ppm)

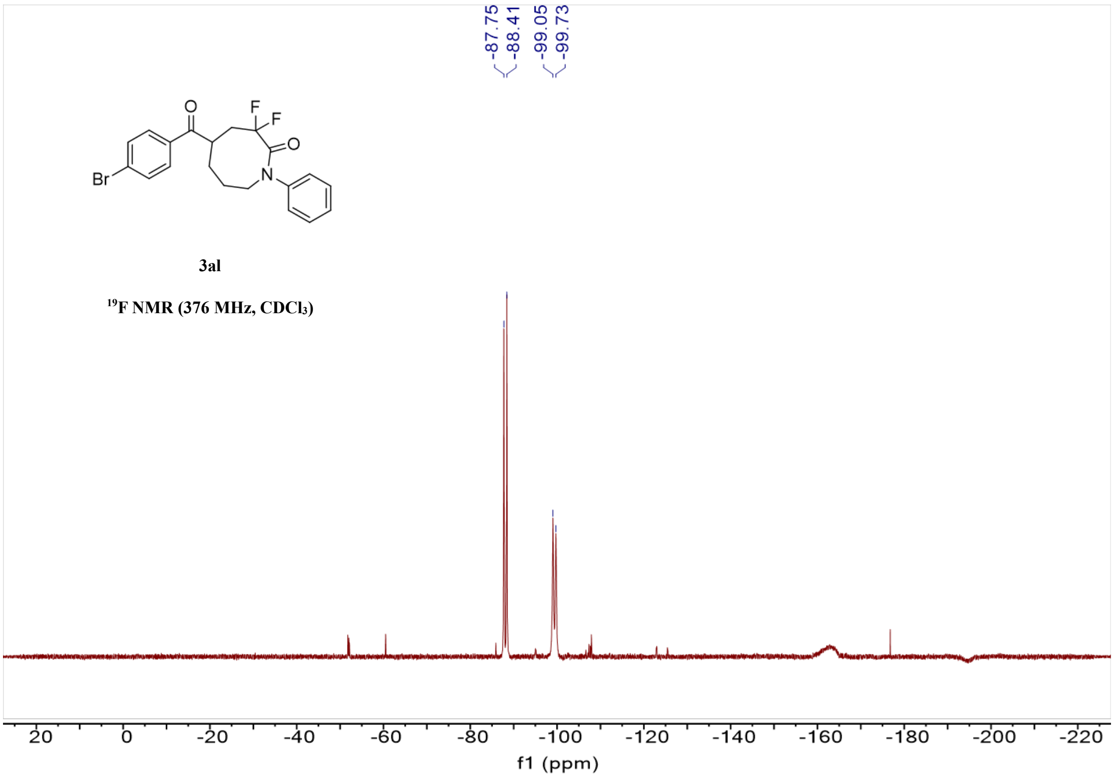

3al

^{19}F NMR (376 MHz, $CDCl_3$)

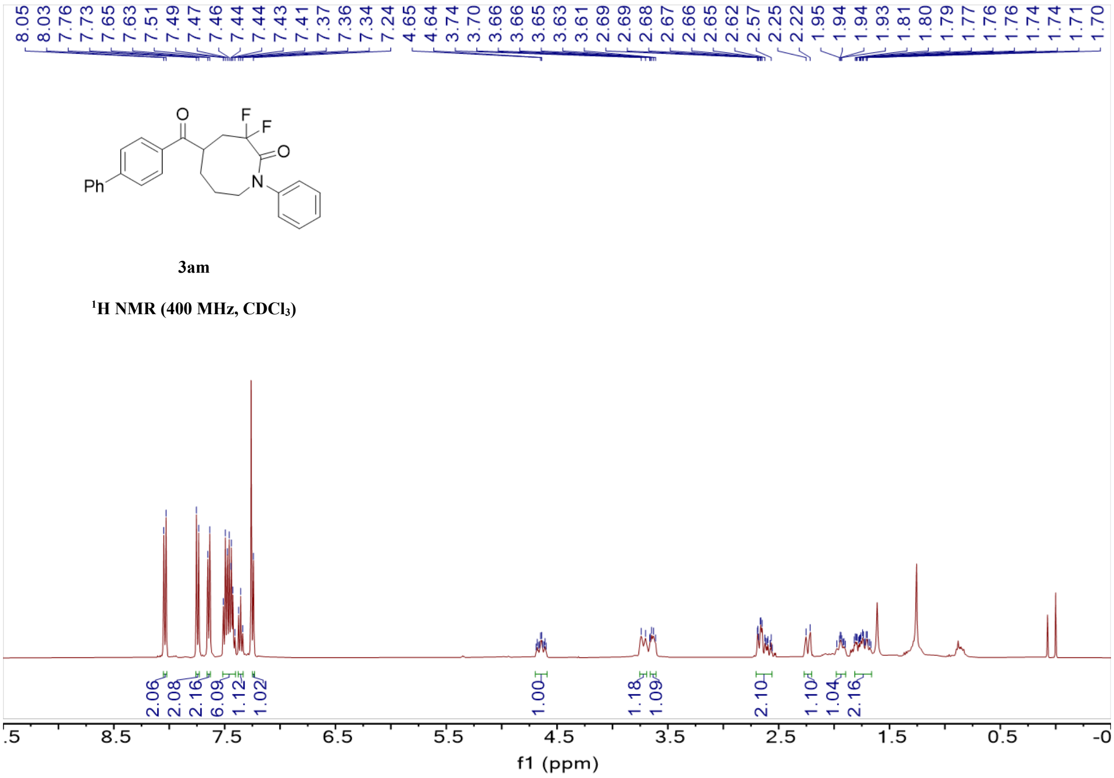
8.05 8.03 7.76 7.73 7.65 7.63 7.51 7.49 7.47 7.46 7.44 7.44 7.43 7.41 7.37 7.36 7.34 7.24 4.65 4.64 3.74 3.70 3.66 3.65 3.63 3.61 2.69 2.69 2.68 2.67 2.66 2.65 2.62 2.57 2.25 2.22 1.95 1.94 1.94 1.93 1.81 1.80 1.79 1.77 1.76 1.76 1.74 1.74 1.71 1.70
O
F
F
O
N
Ph
3am
1H NMR (400 MHz, CDCl3)
2.06 2.08 2.16 6.09 1.12 1.02 1.00 1.18 1.09 2.10 1.10 1.04 2.16
f1 (ppm)

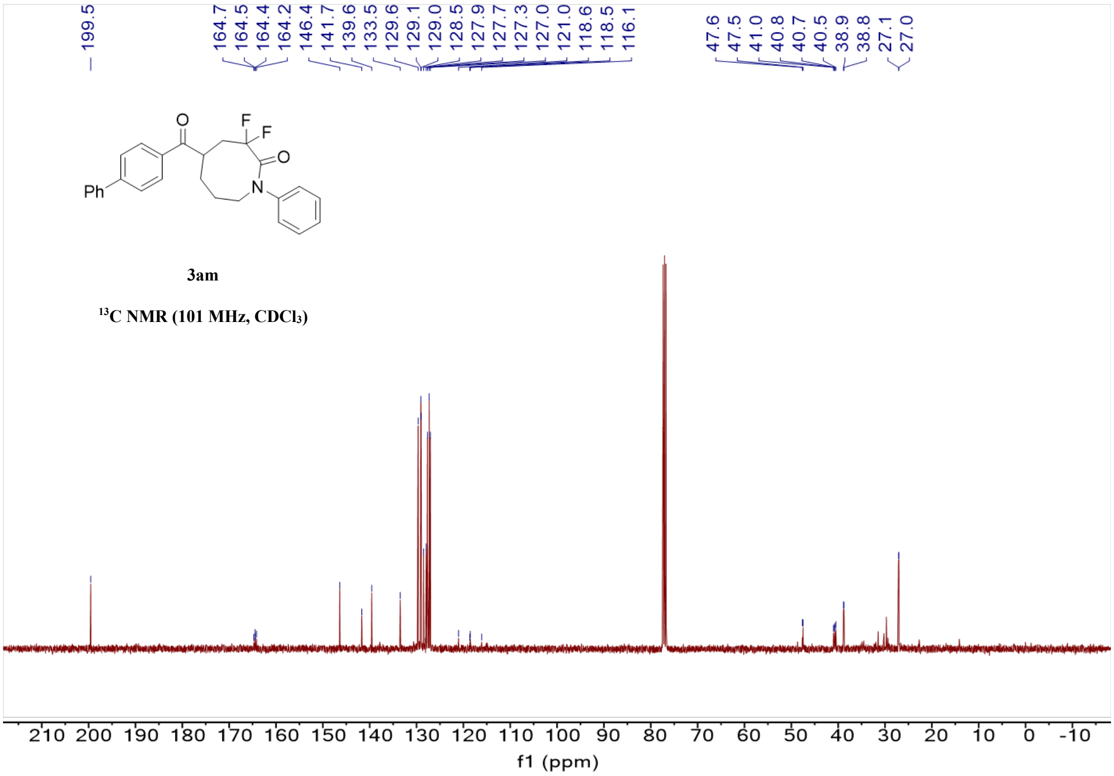
199.5
164.7 164.5 164.4 164.2 146.4 141.7 139.6 133.5 129.6 129.1 129.0 128.5 127.9 127.7 127.3 127.0 121.0 118.6 118.5 116.1
47.6 47.5 41.0 40.8 40.7 40.5 38.9 38.8 27.1 27.0
O
F
F
O
N
Ph
3am
13C NMR (101 MHz, CDCl3)
f1 (ppm)

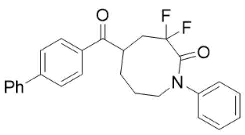

3am

^{19}F NMR (376 MHz, CDCl$_3$)

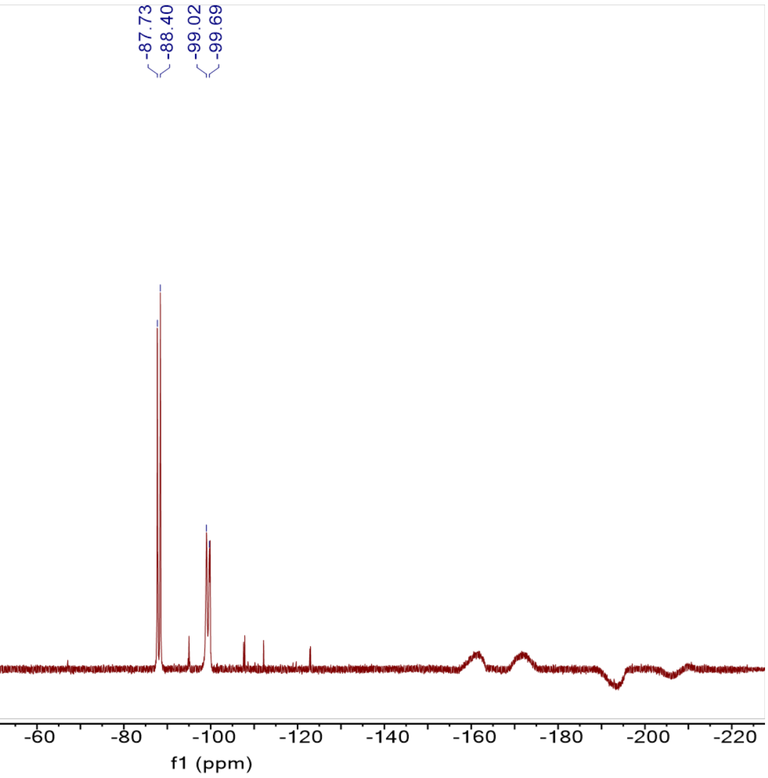

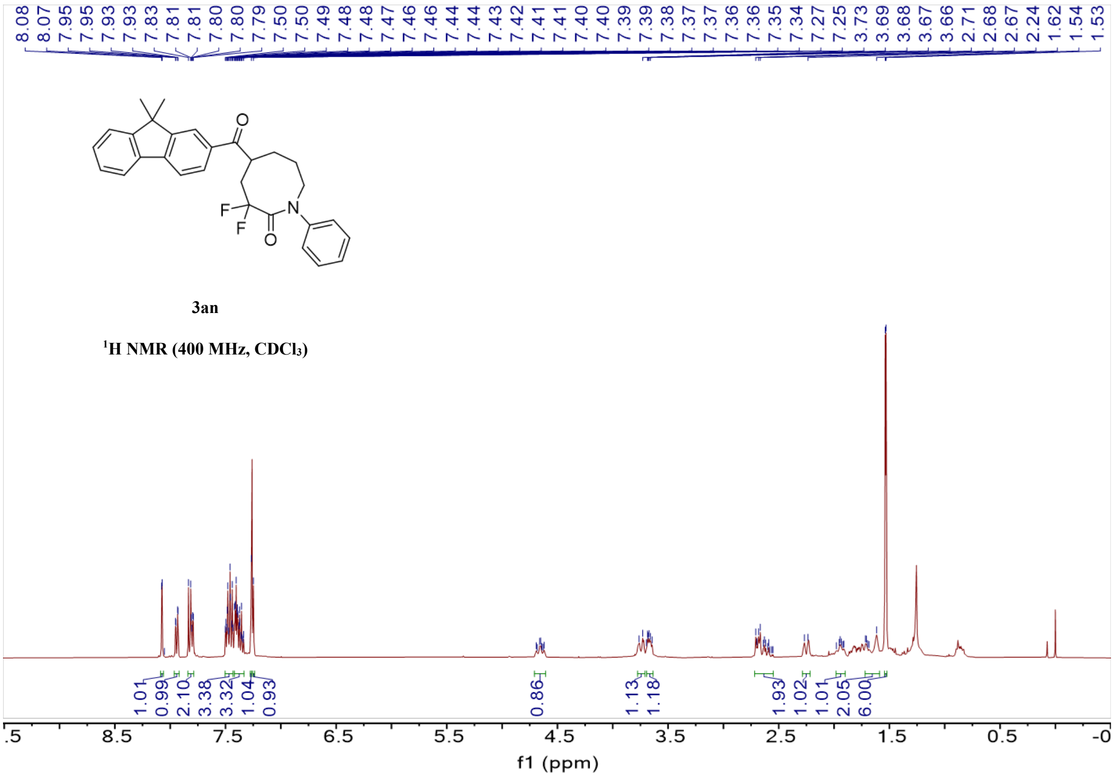
3an
^{1}H NMR (400 MHz, $CDCl_3$)
f1 (ppm)

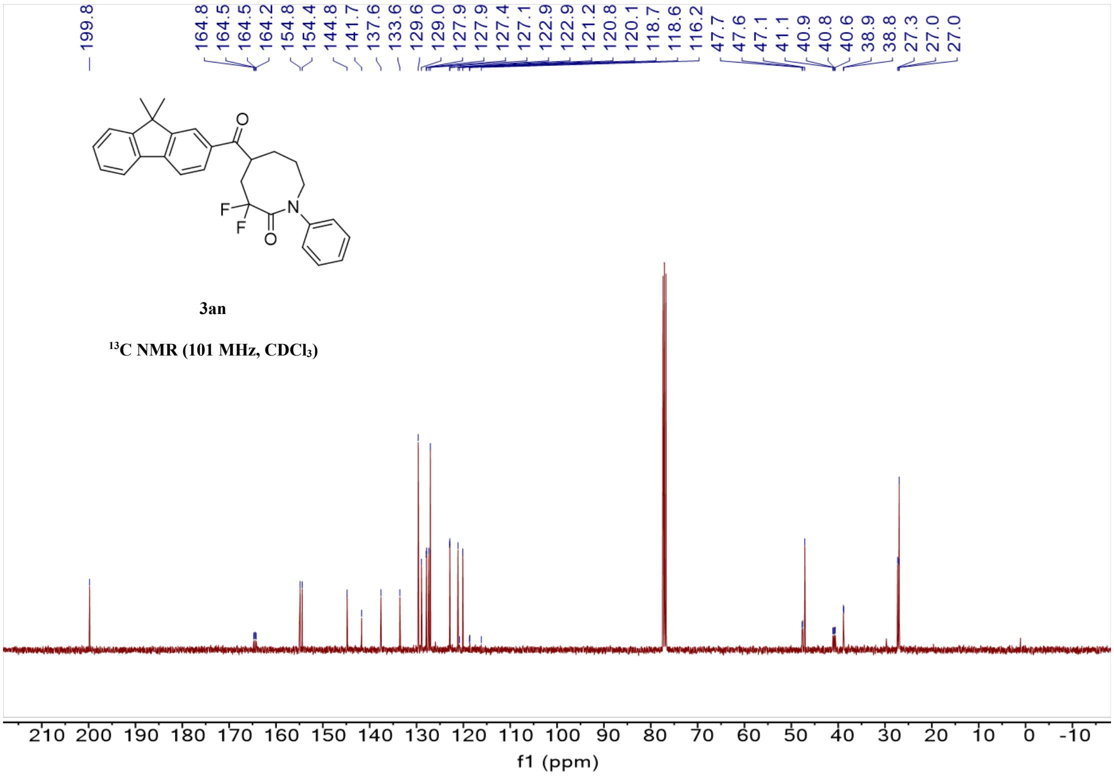
3an
^{13}C NMR (101 MHz, $CDCl_3$)
f1 (ppm)

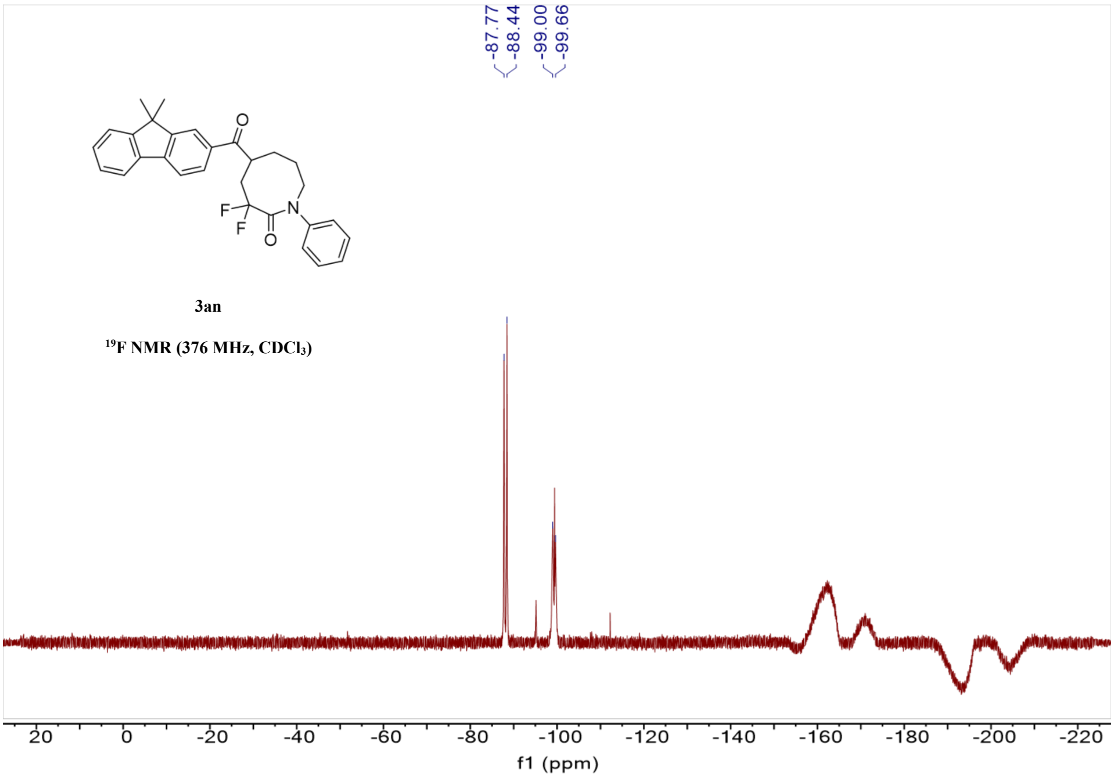

-87.77
-88.44
-99.00
-99.66
3an
^{19}F NMR (376 MHz, CDCl$_3$)
f1 (ppm)

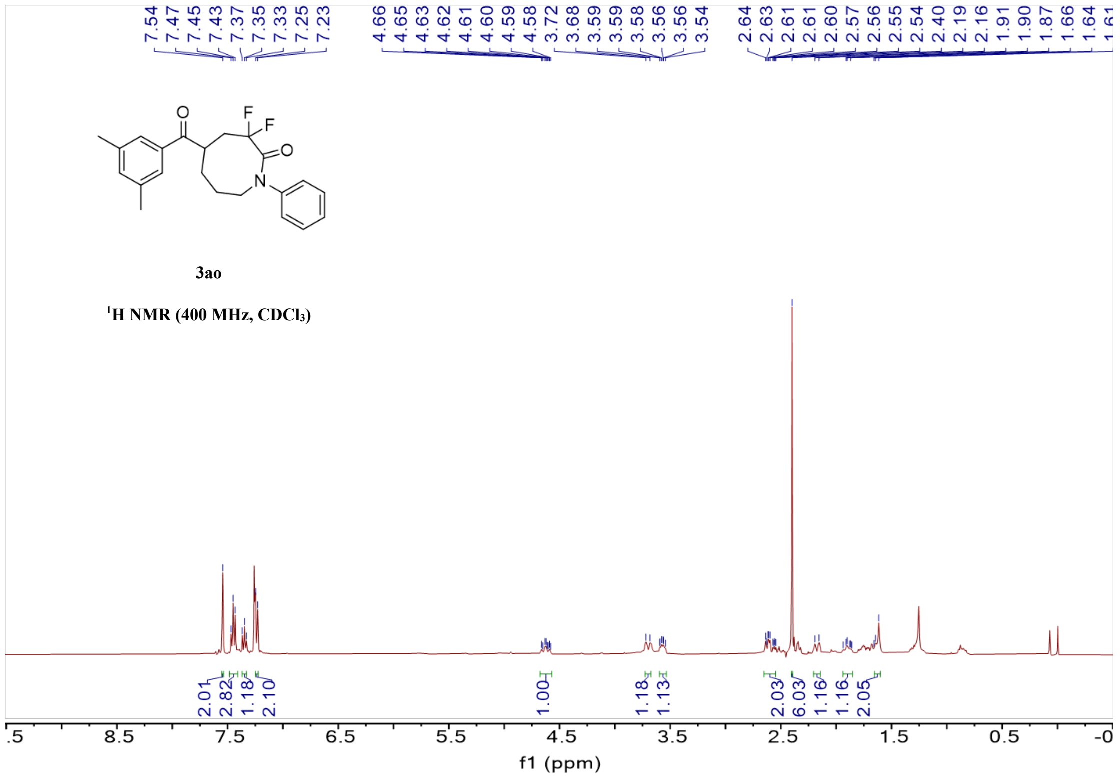

3ao

^{1}H NMR (400 MHz, CDCl$_3$)

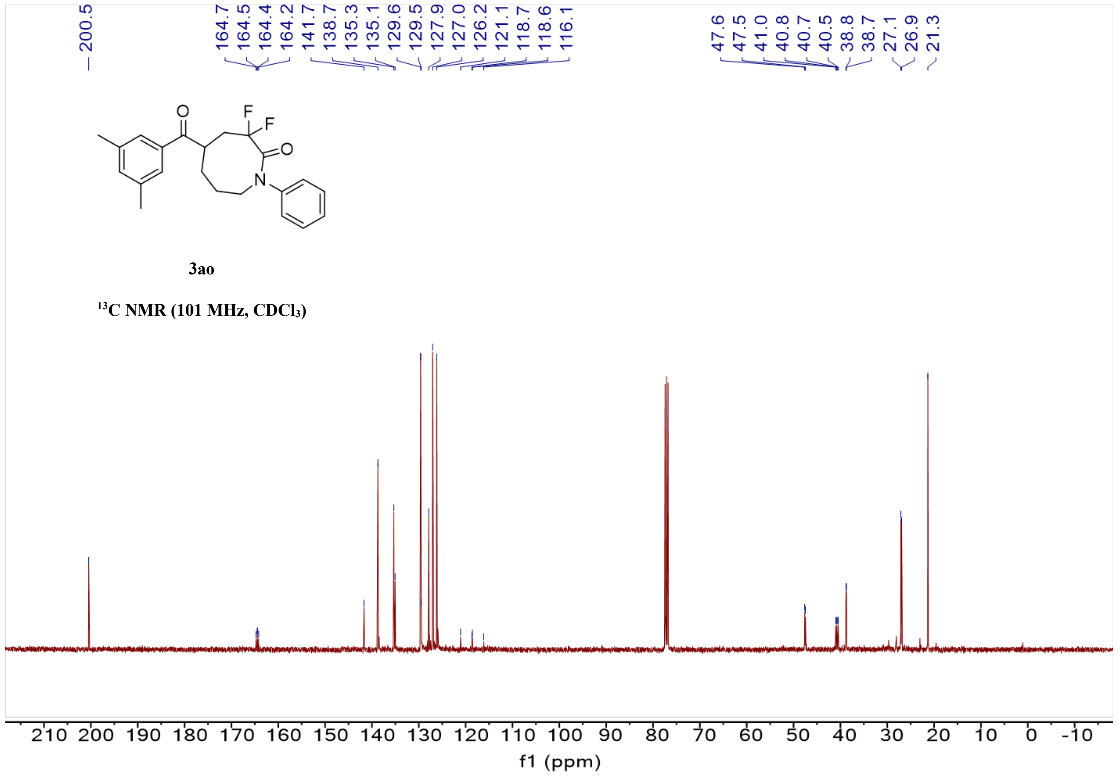

3ao

^{13}C NMR (101 MHz, CDCl$_3$)

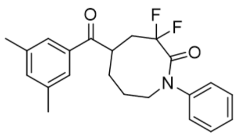

3ao

^{19}F NMR (376 MHz, CDCl$_3$)

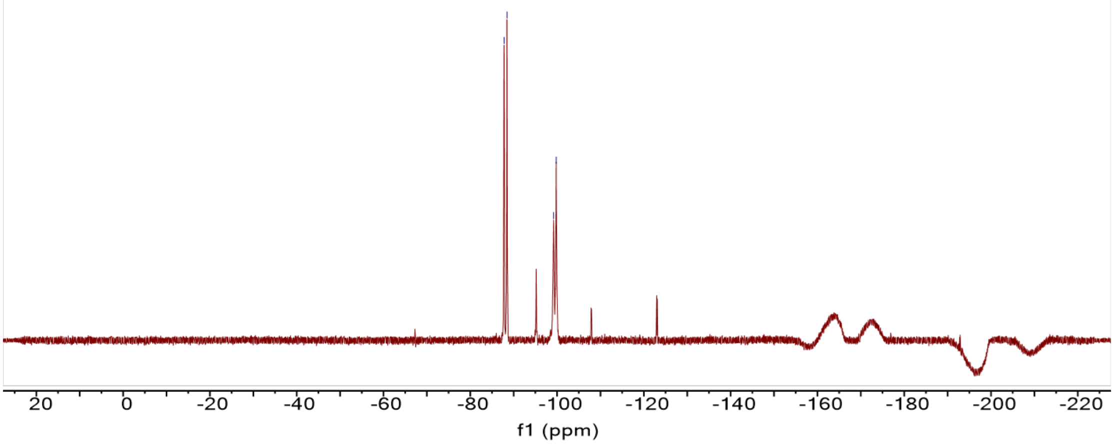

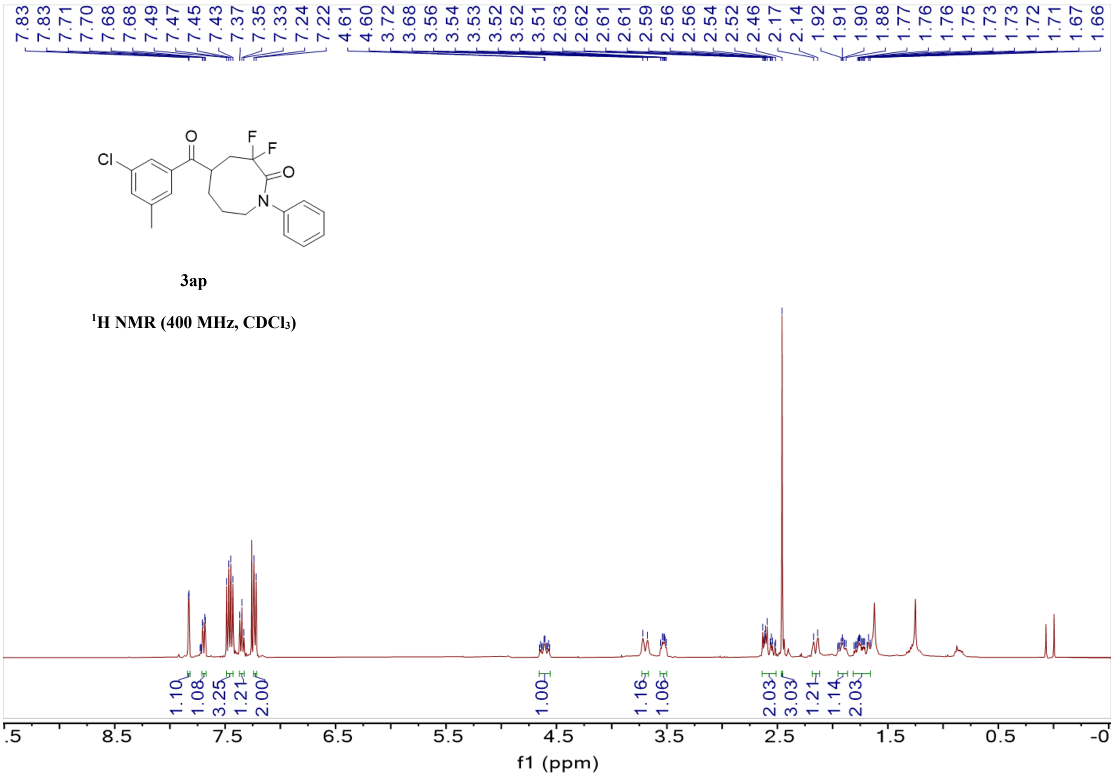
7.83 7.83 7.71 7.70 7.68 7.68 7.49 7.47 7.45 7.43 7.37 7.35 7.33 7.24 7.22 4.61 4.60 3.72 3.68 3.56 3.54 3.53 3.52 3.51 2.63 2.62 2.61 2.59 2.56 2.54 2.52 2.46 2.17 2.14 1.92 1.91 1.90 1.88 1.77 1.76 1.76 1.75 1.73 1.73 1.72 1.71 1.67 1.66
Cl
O
F
F
O
N
3ap
^{1}H NMR (400 MHz, CDCl$_3$)
1.10 1.08 3.25 1.21 2.00 1.00 1.16 1.06 2.03 3.03 1.21 1.14 2.03
.5 8.5 7.5 6.5 5.5 4.5 3.5 2.5 1.5 0.5 -0
f1 (ppm)

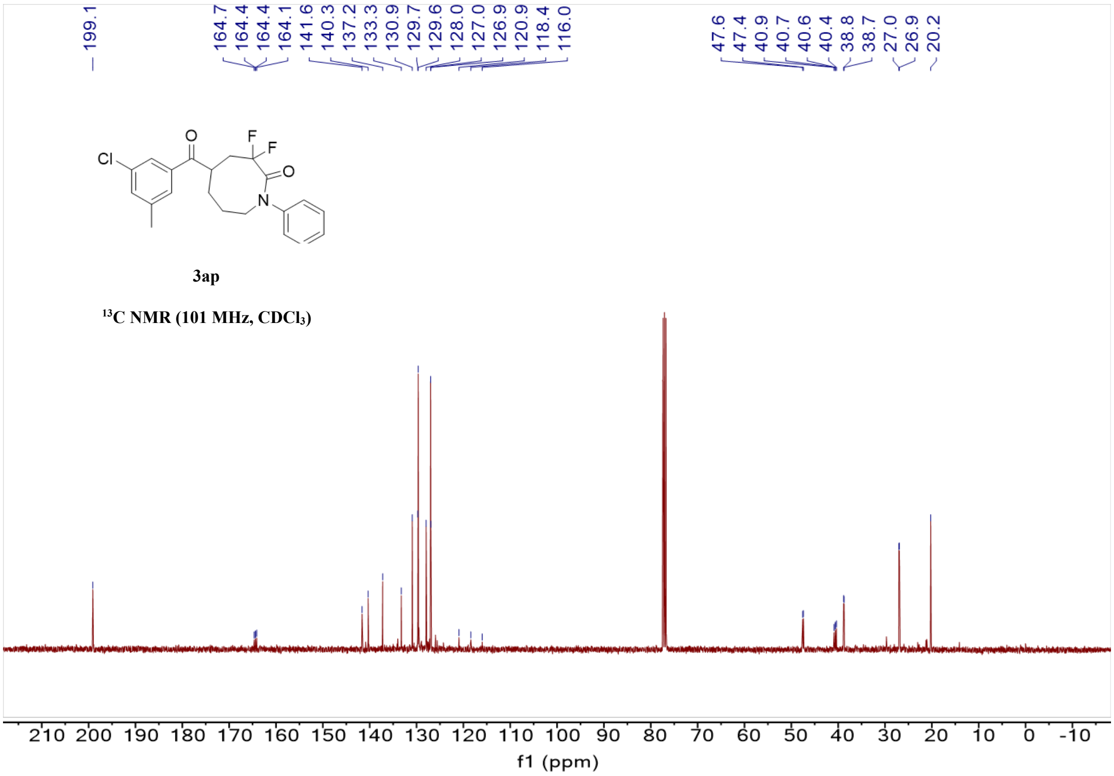
199.1
164.7 164.4 164.4 164.1 141.6 140.3 137.2 133.3 130.9 129.7 129.6 128.0 127.0 126.9 120.9 118.4 116.0
47.6 47.4 40.9 40.7 40.6 40.4 38.8 38.7 27.0 26.9 20.2
Cl
O
F
F
O
N
3ap
^{13}C NMR (101 MHz, CDCl$_3$)
210 200 190 180 170 160 150 140 130 120 110 100 90 80 70 60 50 40 30 20 10 0 -10
f1 (ppm)

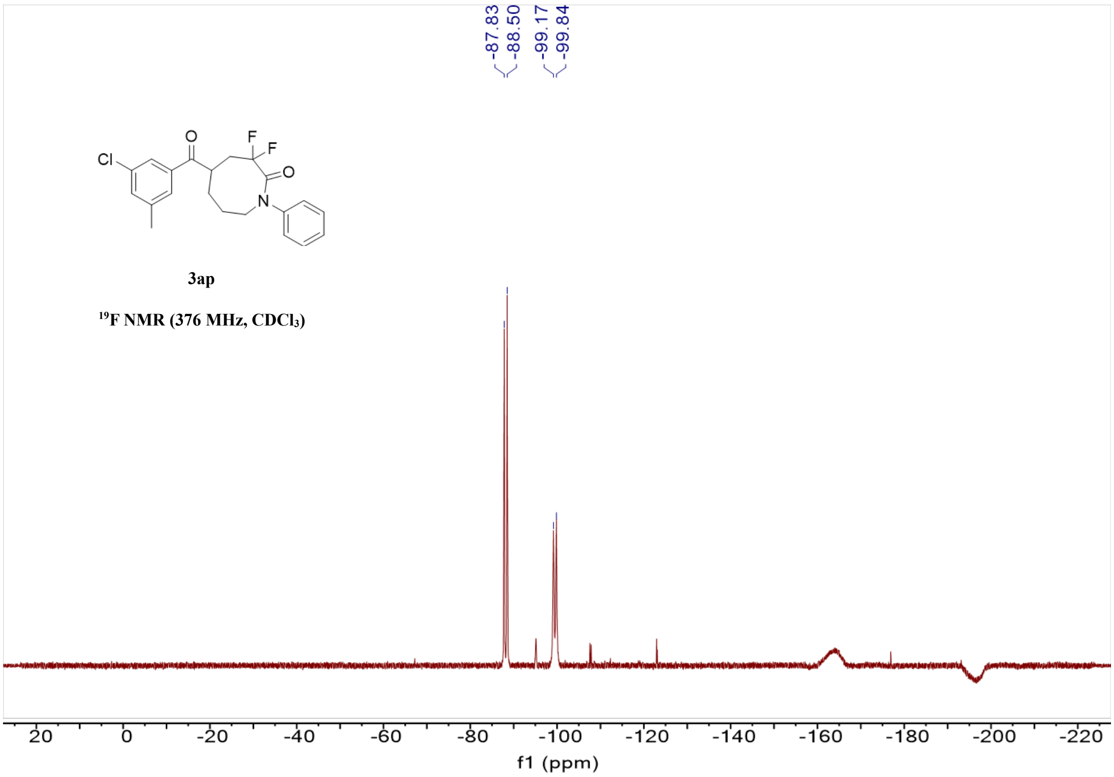

3ap

^{19}F NMR (376 MHz, CDCl$_3$)

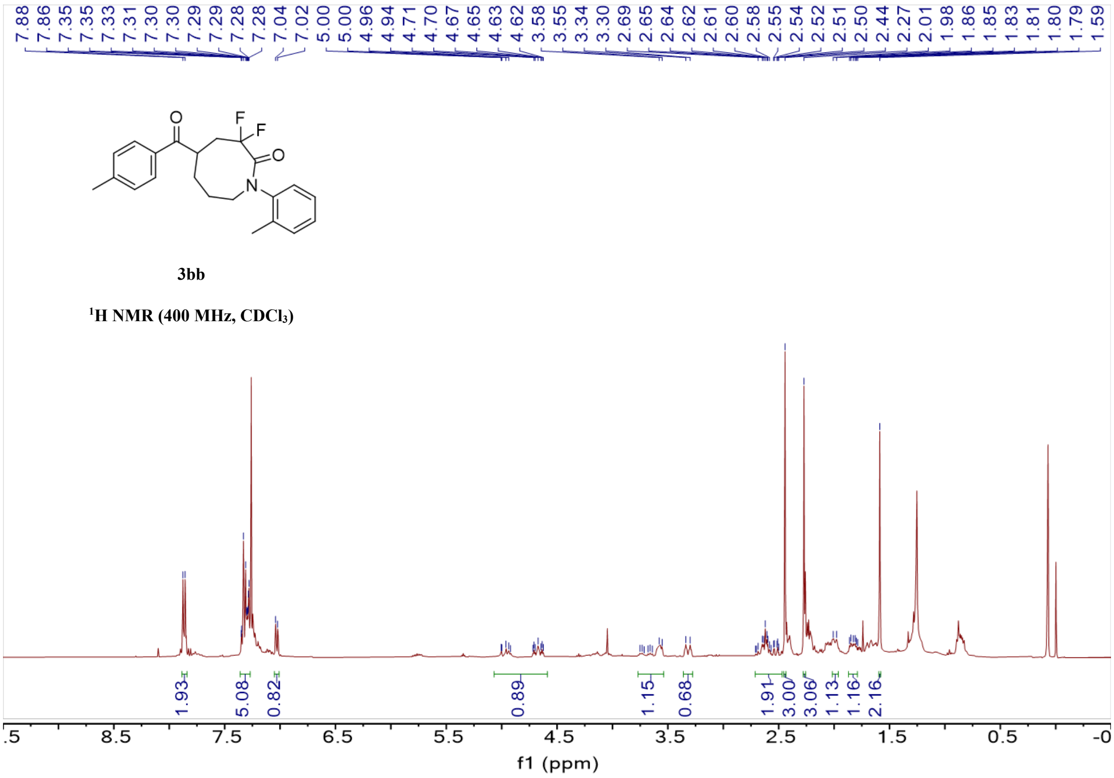
7.88 7.86 7.35 7.35 7.33 7.31 7.30 7.30 7.29 7.29 7.28 7.28 7.04 7.02 5.00 5.00 4.96 4.94 4.71 4.70 4.67 4.65 4.63 4.62 3.58 3.55 3.34 3.30 2.69 2.65 2.64 2.62 2.61 2.60 2.58 2.55 2.54 2.52 2.51 2.50 2.44 2.27 2.01 1.98 1.86 1.85 1.83 1.81 1.80 1.79 1.59
O
F
F
O
N
3bb
1H NMR (400 MHz, CDCl3)
1.93 5.08 0.82 0.89 1.15 0.68 1.91 3.00 3.06 1.13 1.16 2.16
.5 8.5 7.5 6.5 5.5 4.5 3.5 2.5 1.5 0.5 -0
f1 (ppm)

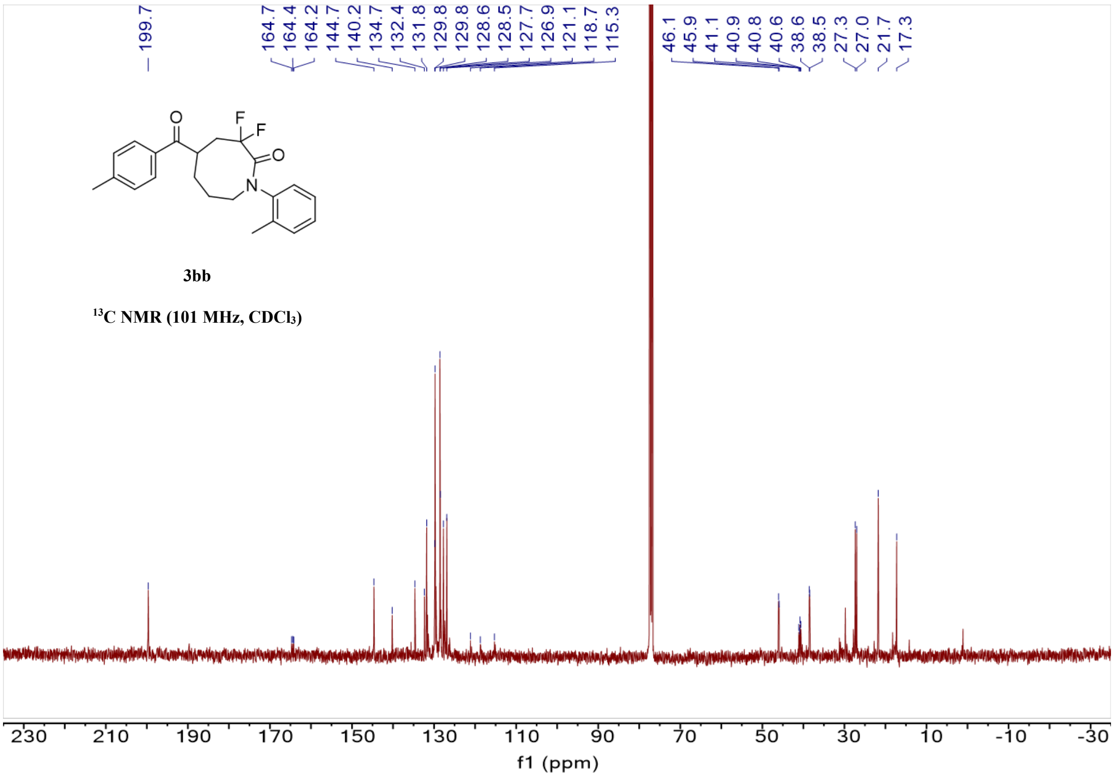
199.7
164.7 164.4 164.2 144.7 140.2 134.7 132.4 131.8 129.8 129.8 128.6 128.5 127.7 126.9 121.1 118.7 115.3
46.1 45.9 41.1 40.9 40.8 40.6 38.6 38.5 27.3 27.0 21.7 17.3
O
F
F
O
N
3bb
13C NMR (101 MHz, CDCl3)
230 210 190 170 150 130 110 90 70 50 30 10 -10 -30
f1 (ppm)

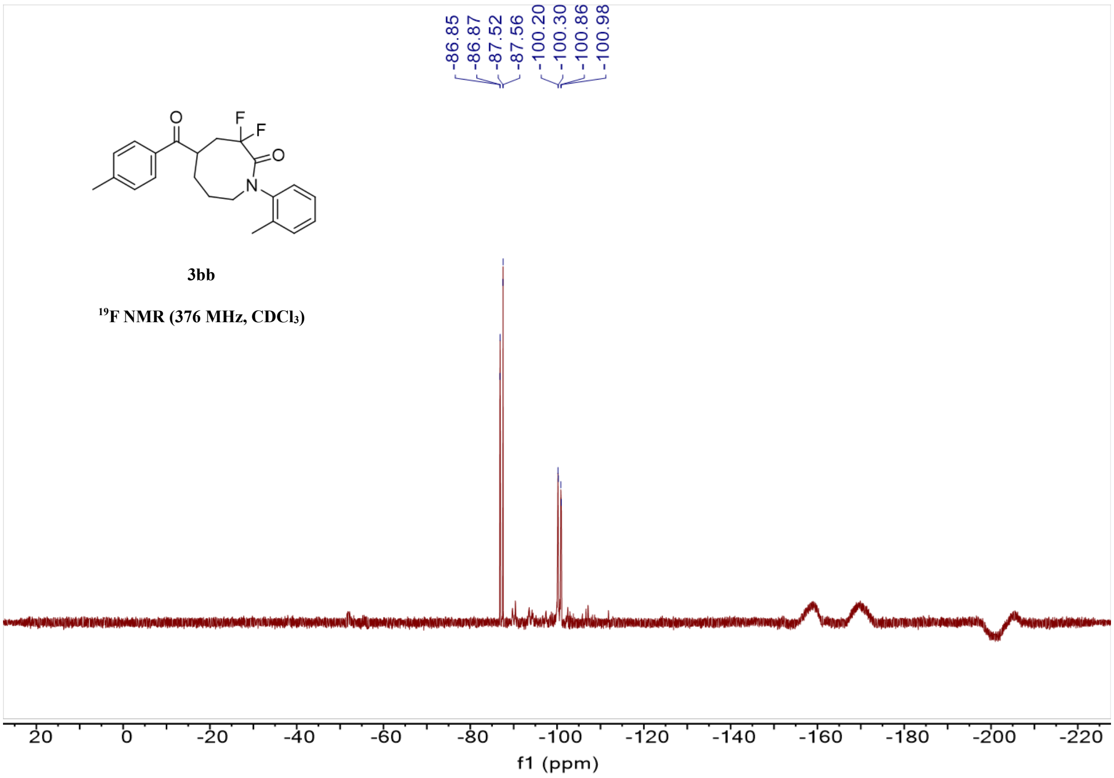

-86.85
-86.87
-87.52
-87.56
-100.20
-100.30
-100.86
-100.98
3bb
19F NMR (376 MHz, CDCl3)
20 0 -20 -40 -60 -80 -100 -120 -140 -160 -180 -200 -220
f1 (ppm)

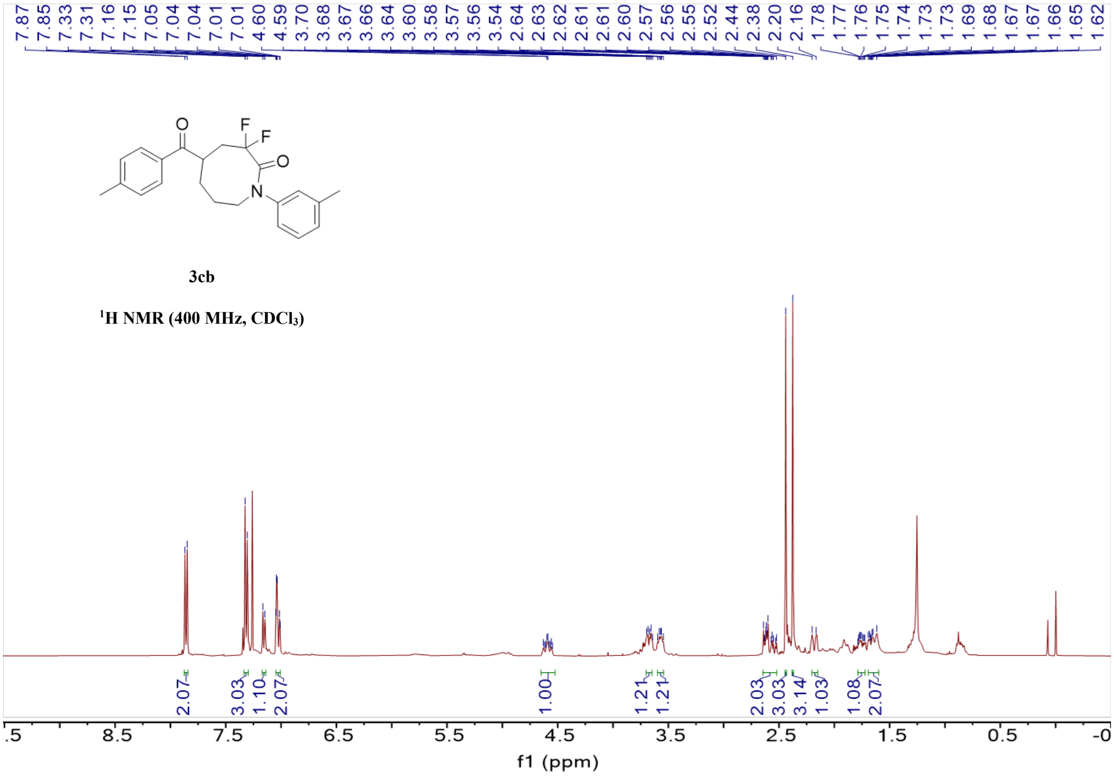
7.87 7.85 7.33 7.31 7.16 7.15 7.05 7.04 7.04 7.01 7.01 4.60 4.59 3.70 3.68 3.67 3.66 3.64 3.60 3.58 3.57 3.56 3.54 2.64 2.63 2.62 2.61 2.61 2.60 2.57 2.56 2.55 2.52 2.44 2.38 2.20 2.16 1.78 1.77 1.76 1.75 1.74 1.73 1.73 1.69 1.68 1.67 1.67 1.66 1.65 1.62
O
F
F
O
N
3cb
^{1}H NMR (400 MHz, CDCl$_3$)
2.07 3.03 1.10 2.07 1.00 1.21 1.21 2.03 3.03 3.14 1.03 1.08 2.07
f1 (ppm)

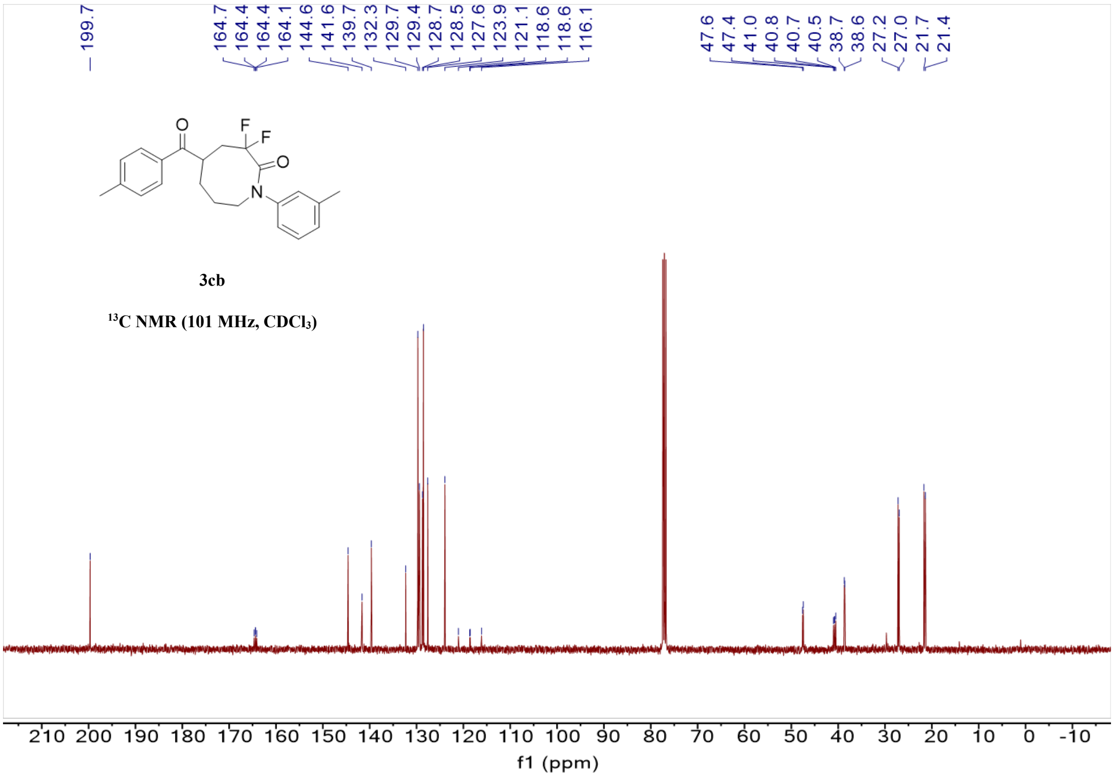
199.7
164.7 164.4 164.4 164.1 144.6 141.6 139.7 132.3 129.7 129.4 128.7 128.5 127.6 123.9 121.1 118.6 118.6 116.1
47.6 47.4 41.0 40.8 40.7 40.5 38.7 38.6 27.2 27.0 21.7 21.4
O
F
F
O
N
3cb
^{13}C NMR (101 MHz, CDCl$_3$)
f1 (ppm)

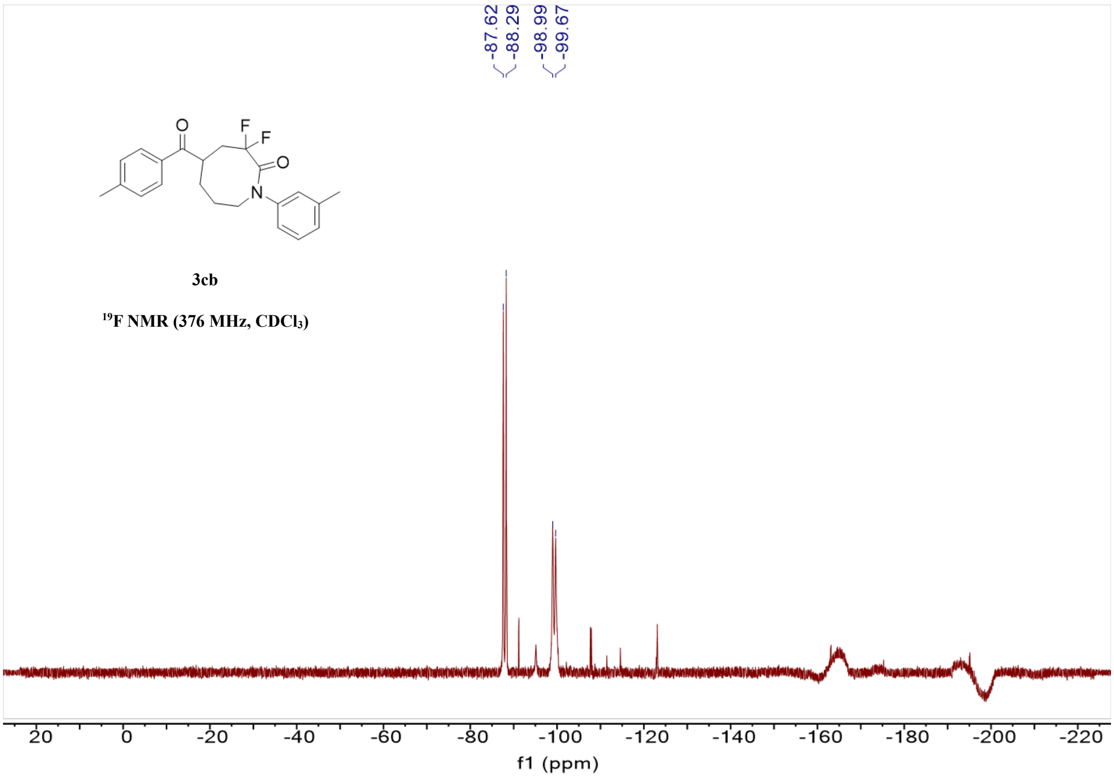

3cb

^{19}F NMR (376 MHz, $CDCl_3$)

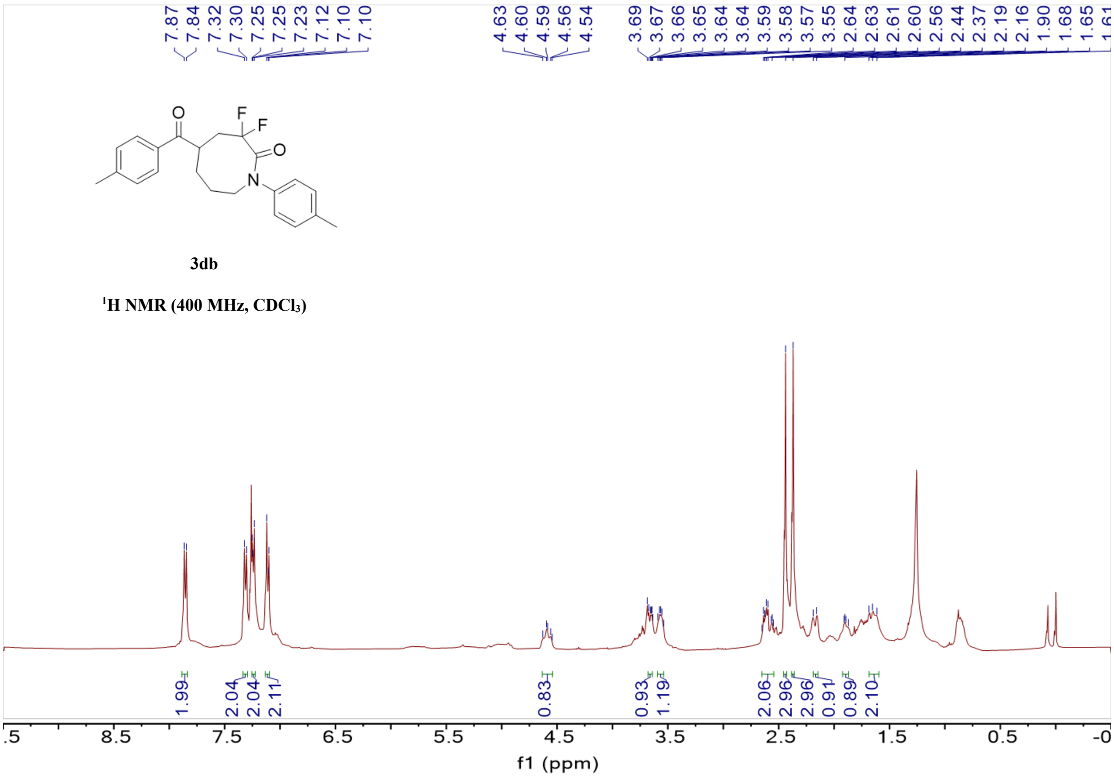

3db

^{1}H NMR (400 MHz, $CDCl_3$)

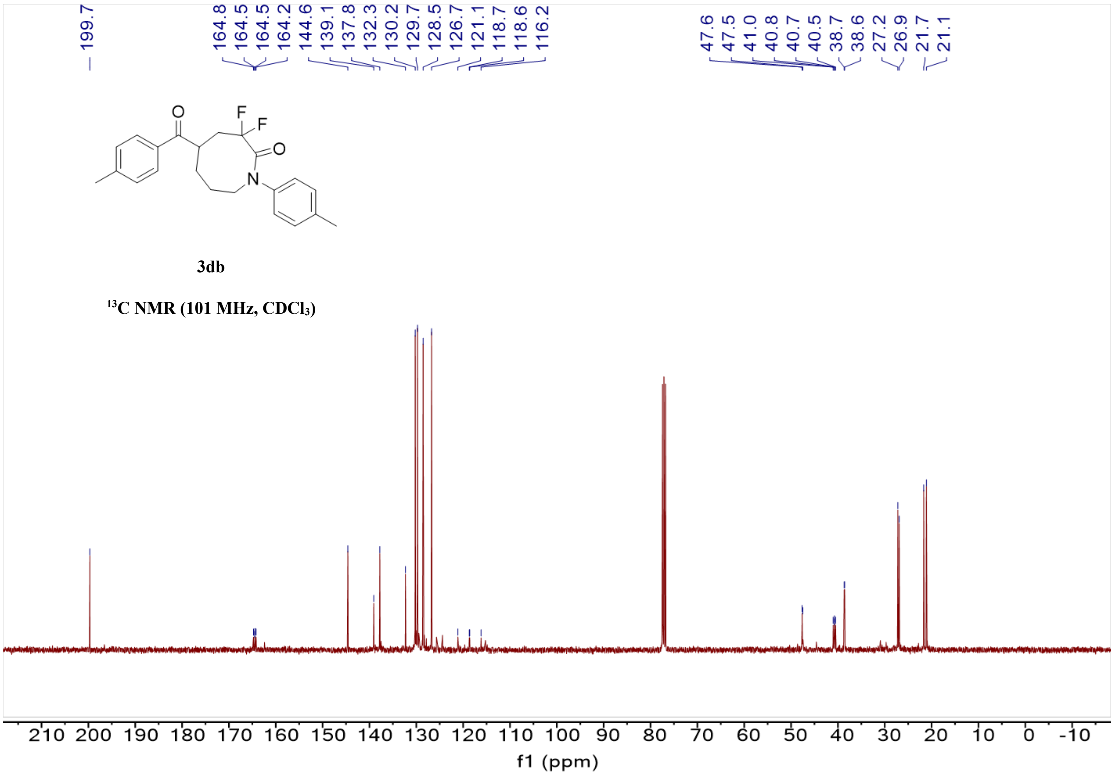

3db

^{13}C NMR (101 MHz, $CDCl_3$)

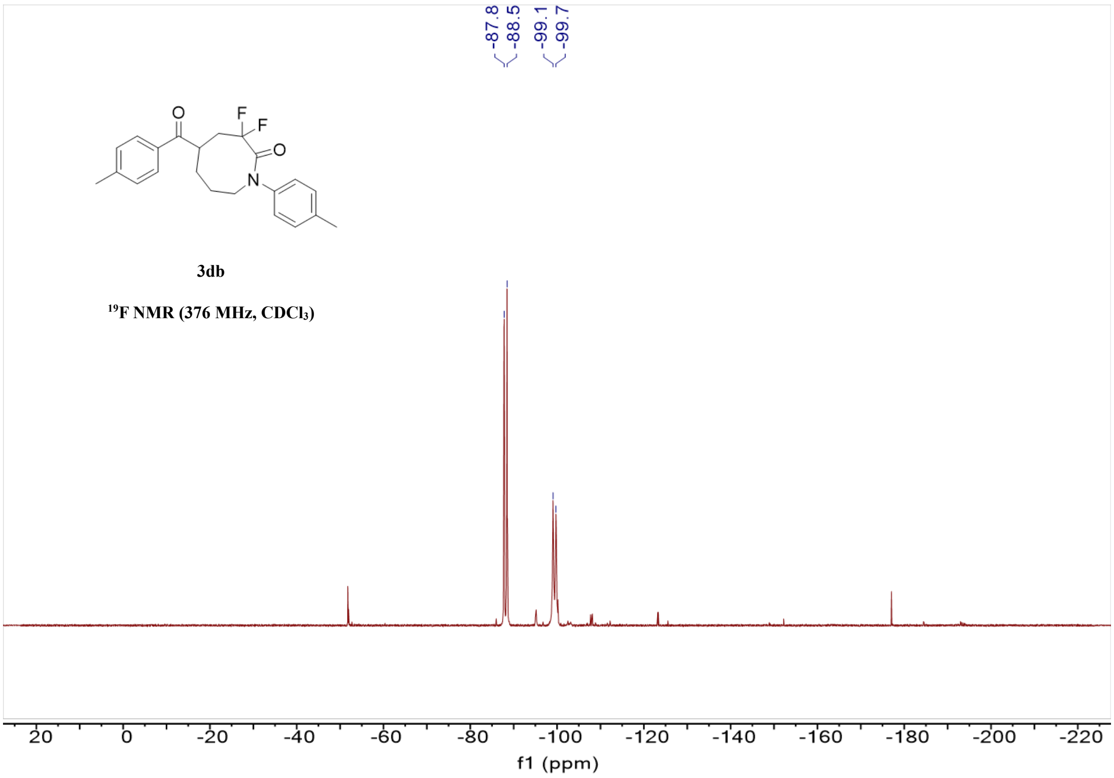

3db

^{19}F NMR (376 MHz, $CDCl_3$)

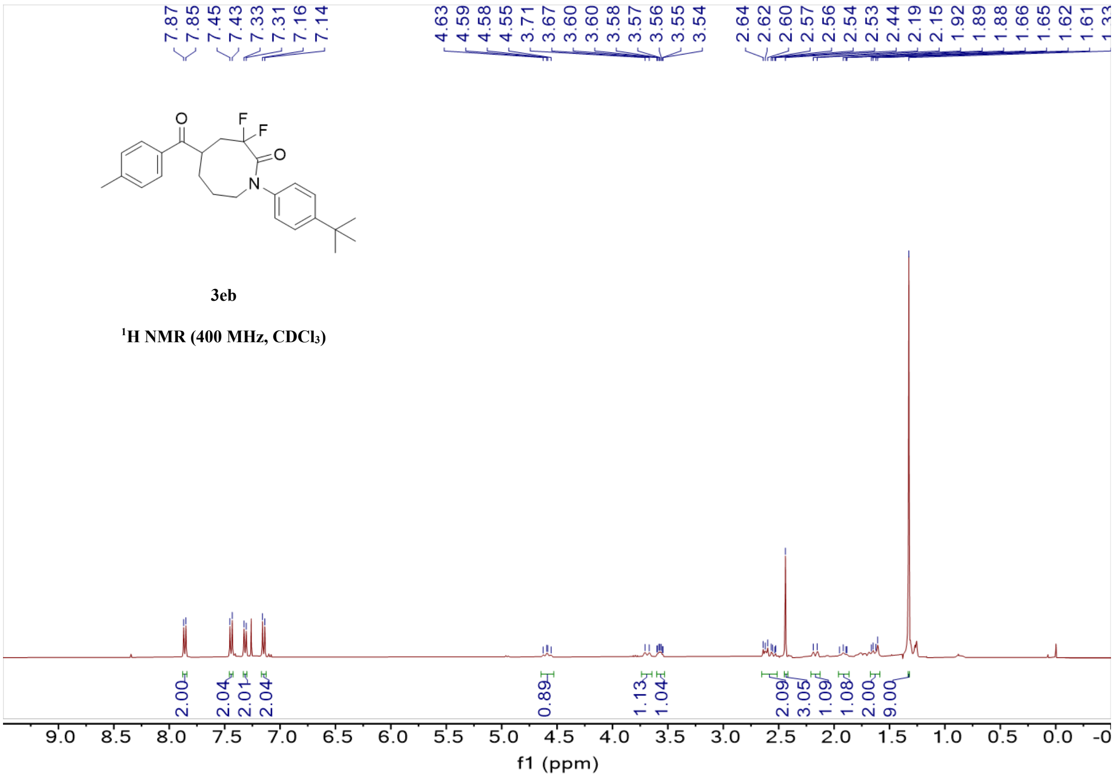
7.87 7.85 7.45 7.43 7.33 7.31 7.16 7.14
4.63 4.59 4.58 4.55 3.71 3.67 3.60 3.60 3.58 3.57 3.56 3.55 3.54
2.64 2.62 2.60 2.57 2.56 2.54 2.53 2.44 2.19 2.15 1.92 1.89 1.88 1.66 1.65 1.62 1.61 1.33
O
F
F
O
N
3eb
^{1}H NMR (400 MHz, $CDCl_3$)
2.00 2.04 2.01 2.04 0.89 1.13 1.04 2.09 3.05 1.09 1.08 2.00 9.00
9.0 8.5 8.0 7.5 7.0 6.5 6.0 5.5 5.0 4.5 4.0 3.5 3.0 2.5 2.0 1.5 1.0 0.5 0.0 -0
f1 (ppm)

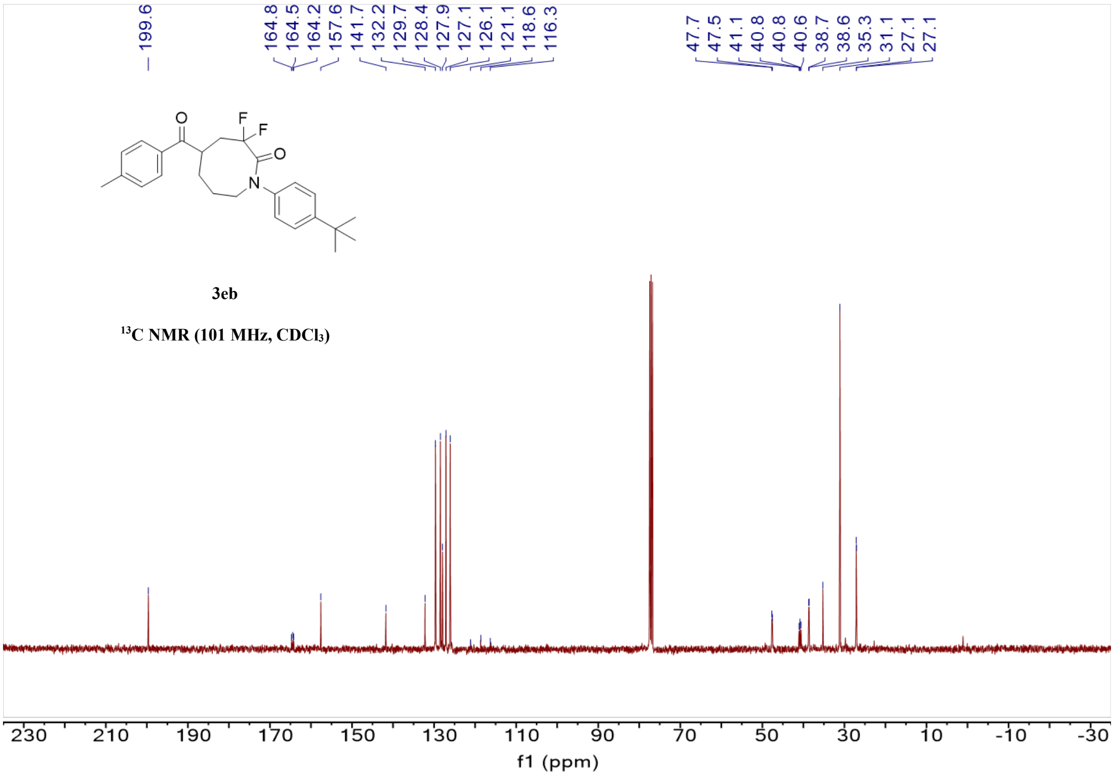
199.6
164.8 164.5 164.2 157.6 141.7 132.2 129.7 128.4 127.9 127.1 126.1 121.1 118.6 116.3
47.7 47.5 41.1 40.8 40.8 40.6 38.7 38.6 35.3 31.1 27.1 27.1
O
F
F
O
N
3eb
^{13}C NMR (101 MHz, $CDCl_3$)
230 210 190 170 150 130 110 90 70 50 30 10 -10 -30
f1 (ppm)

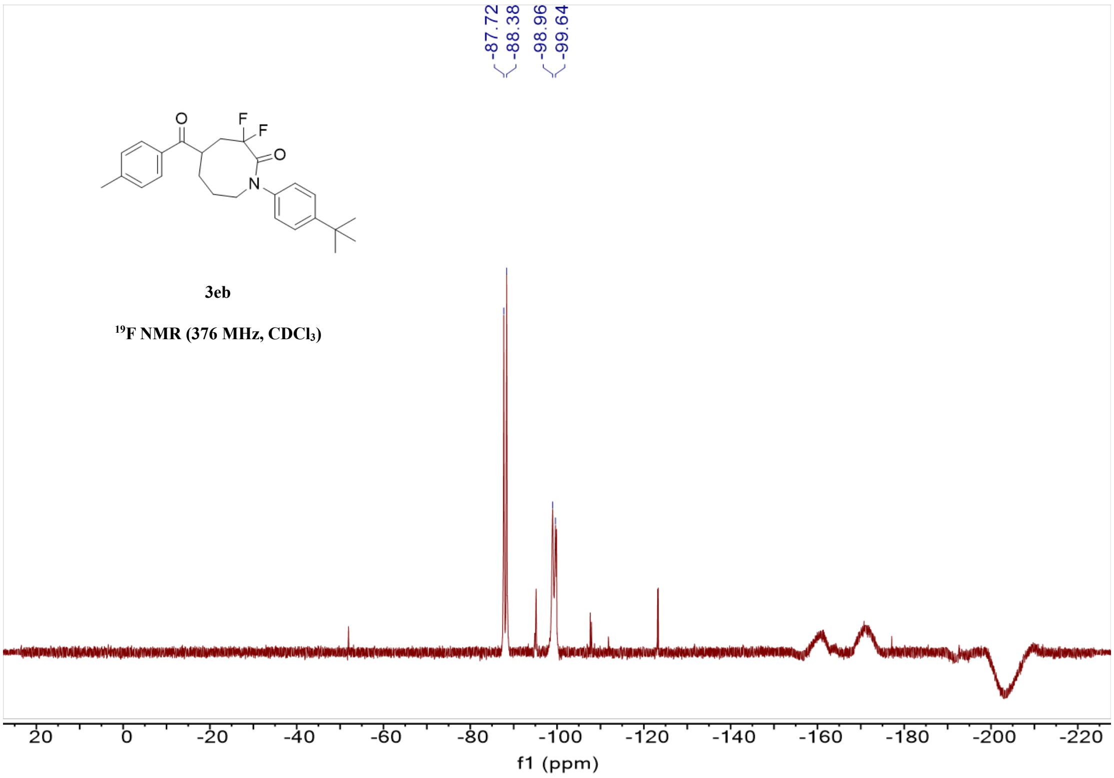
-87.72
-88.38
-98.96
-99.64
O
F
F
O
N
3eb
^{19}F NMR (376 MHz, $CDCl_3$)
20
0
-20
-40
-60
-80
-100
-120
-140
-160
-180
-200
-220
f1 (ppm)

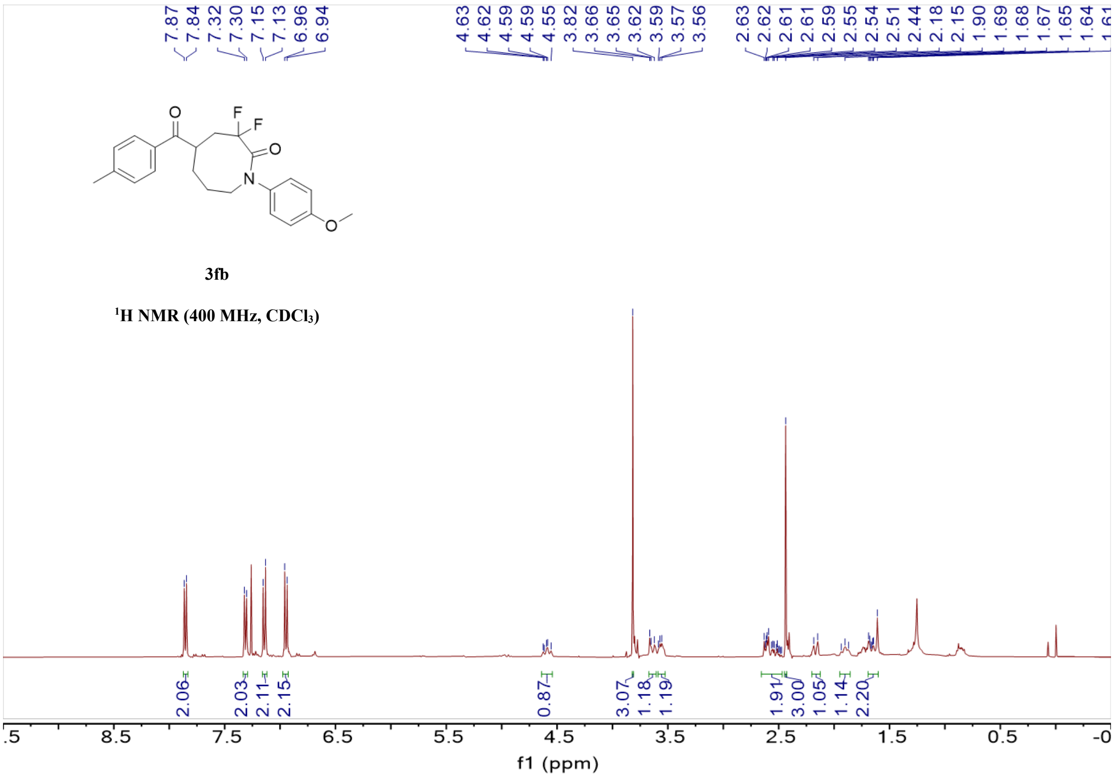
7.87 7.84 7.32 7.30 7.15 7.13 6.96 6.94
4.63 4.62 4.59 4.59 4.55 3.82 3.66 3.65 3.62 3.59 3.57 3.56
2.63 2.62 2.61 2.61 2.59 2.55 2.54 2.51 2.44 2.18 2.15 1.90 1.69 1.68 1.67 1.65 1.64 1.61
3fb
^{1}H NMR (400 MHz, CDCl$_3$)
2.06 2.03 2.11 2.15 0.87 3.07 1.18 1.19 1.91 3.00 1.05 1.14 2.20
f1 (ppm)

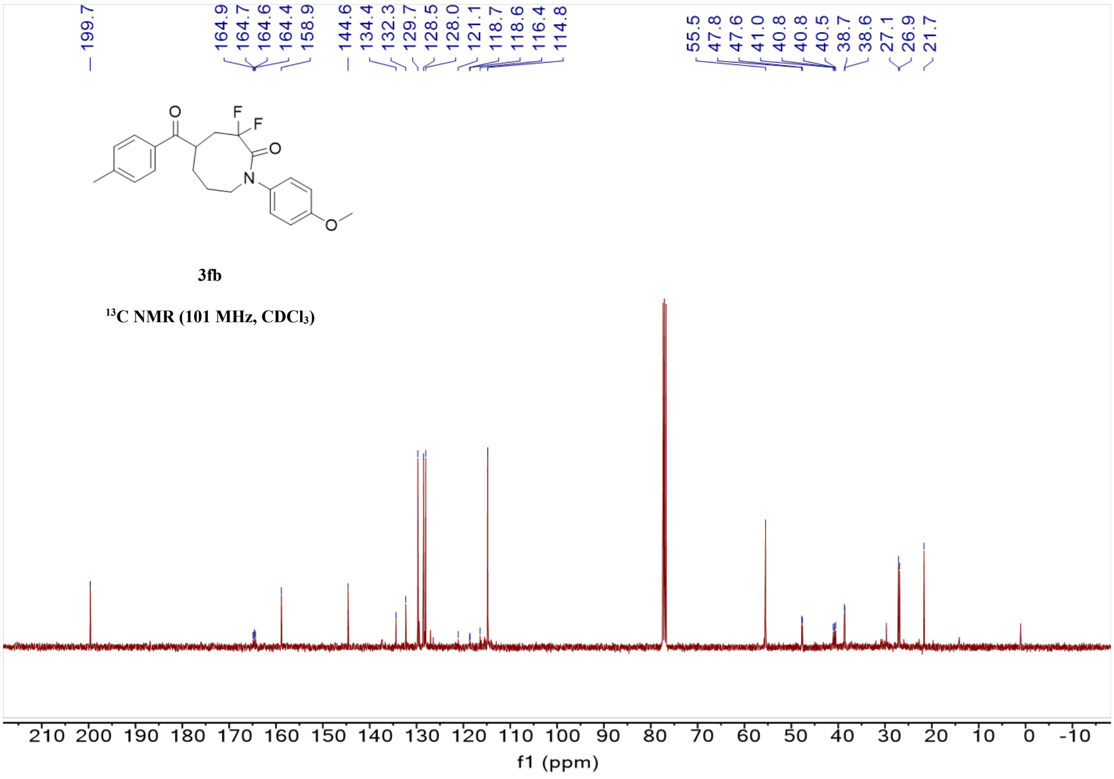
199.7
164.9 164.7 164.6 164.4 158.9
144.6 134.4 132.3 129.7 128.5 128.0 121.1 118.7 118.6 116.4 114.8
55.5 47.8 47.6 41.0 40.8 40.8 40.5 38.7 38.6 27.1 26.9 21.7
3fb
^{13}C NMR (101 MHz, CDCl$_3$)
f1 (ppm)

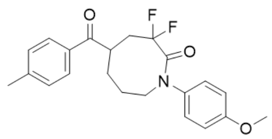

3fb

^{19}F NMR (376 MHz, CDCl$_3$)

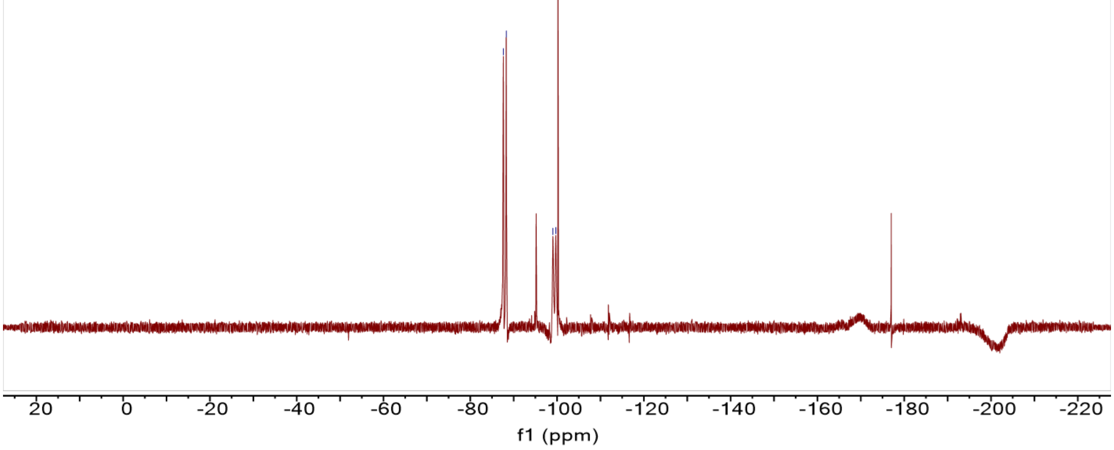

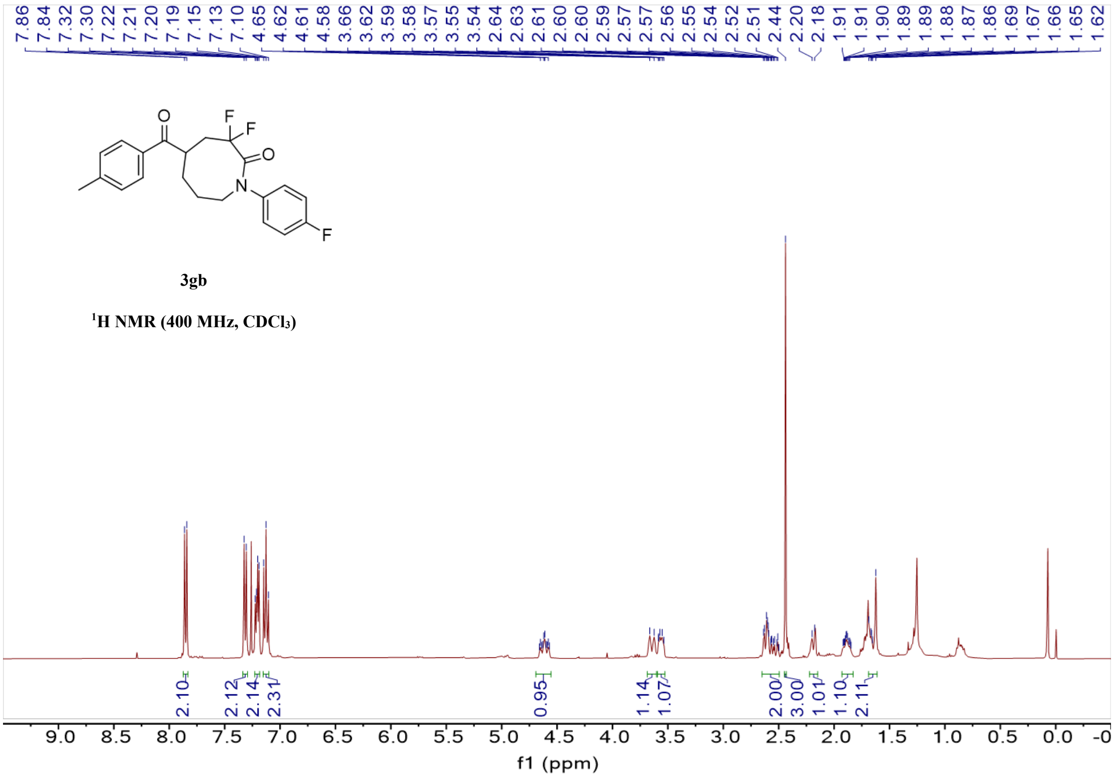
7.86 7.84 7.32 7.30 7.22 7.21 7.20 7.19 7.15 7.13 7.10 4.65 4.62 4.61 4.58 3.66 3.62 3.59 3.58 3.57 3.55 3.54 2.64 2.63 2.61 2.60 2.60 2.59 2.57 2.57 2.56 2.55 2.54 2.52 2.51 2.44 2.20 2.18 1.91 1.91 1.90 1.89 1.89 1.88 1.87 1.86 1.69 1.67 1.66 1.65 1.62
O
F
F
O
N
F
3gb
¹H NMR (400 MHz, CDCl₃)
2.10 2.12 2.14 2.31 0.95 1.14 1.07 2.00 3.00 1.01 1.10 2.11
9.0 8.5 8.0 7.5 7.0 6.5 6.0 5.5 5.0 4.5 4.0 3.5 3.0 2.5 2.0 1.5 1.0 0.5 0.0 -0
f1 (ppm)

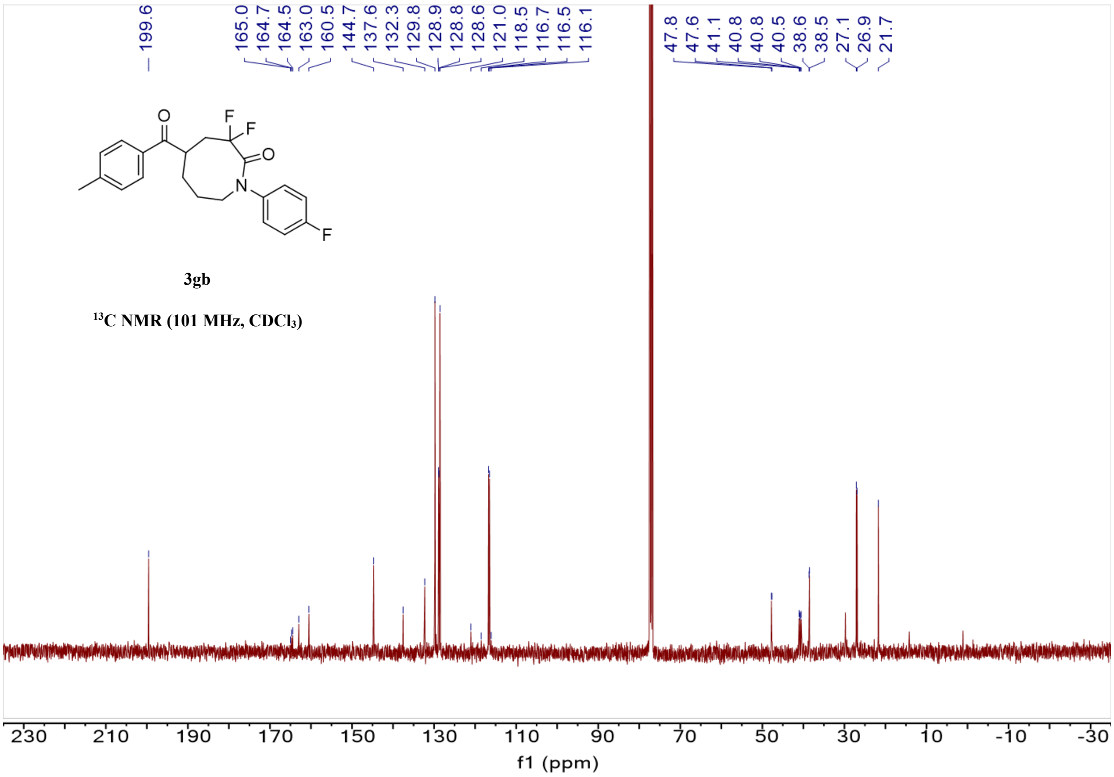
199.6
165.0 164.7 164.5 163.0 160.5 144.7 137.6 132.3 129.8 128.9 128.8 128.6 121.0 118.5 116.7 116.5 116.1
47.8 47.6 41.1 40.8 40.8 40.5 38.6 38.5 27.1 26.9 21.7
O
F
F
O
N
F
3gb
¹³C NMR (101 MHz, CDCl₃)
230 210 190 170 150 130 110 90 70 50 30 10 -10 -30
f1 (ppm)

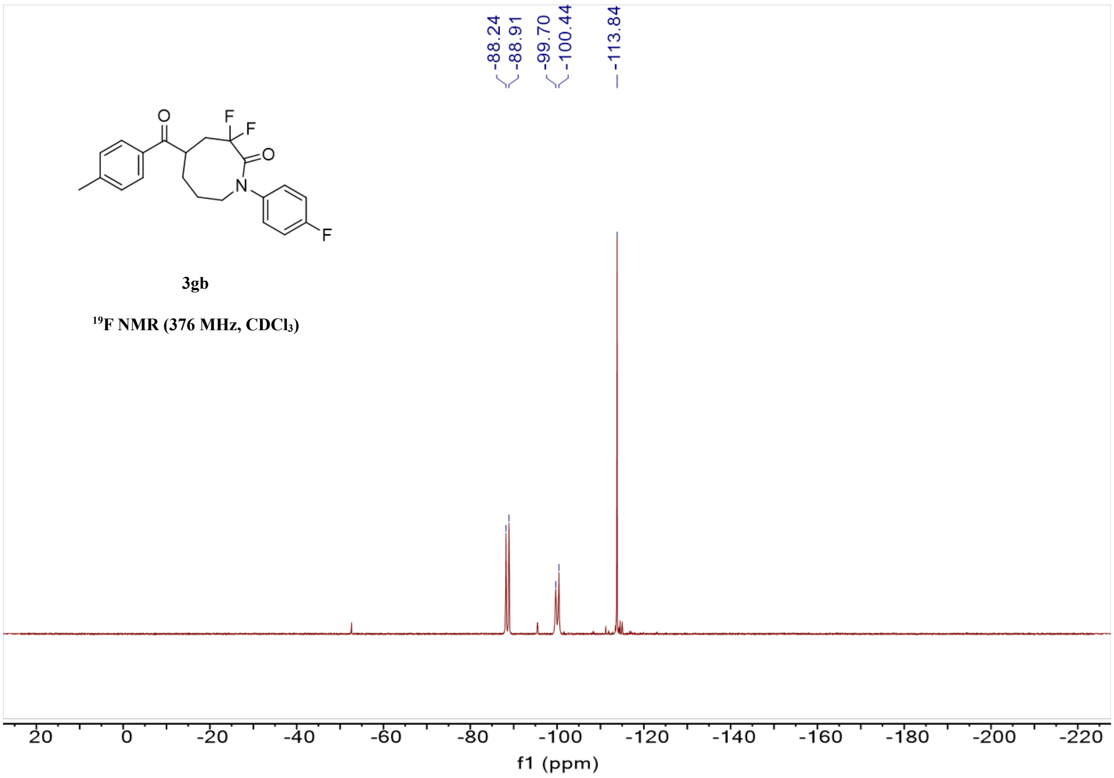

-88.24
-88.91
-99.70
-100.44
-113.84
3gb
19F NMR (376 MHz, CDCl3)
20
0
-20
-40
-60
-80
-100
-120
-140
-160
-180
-200
-220
f1 (ppm)

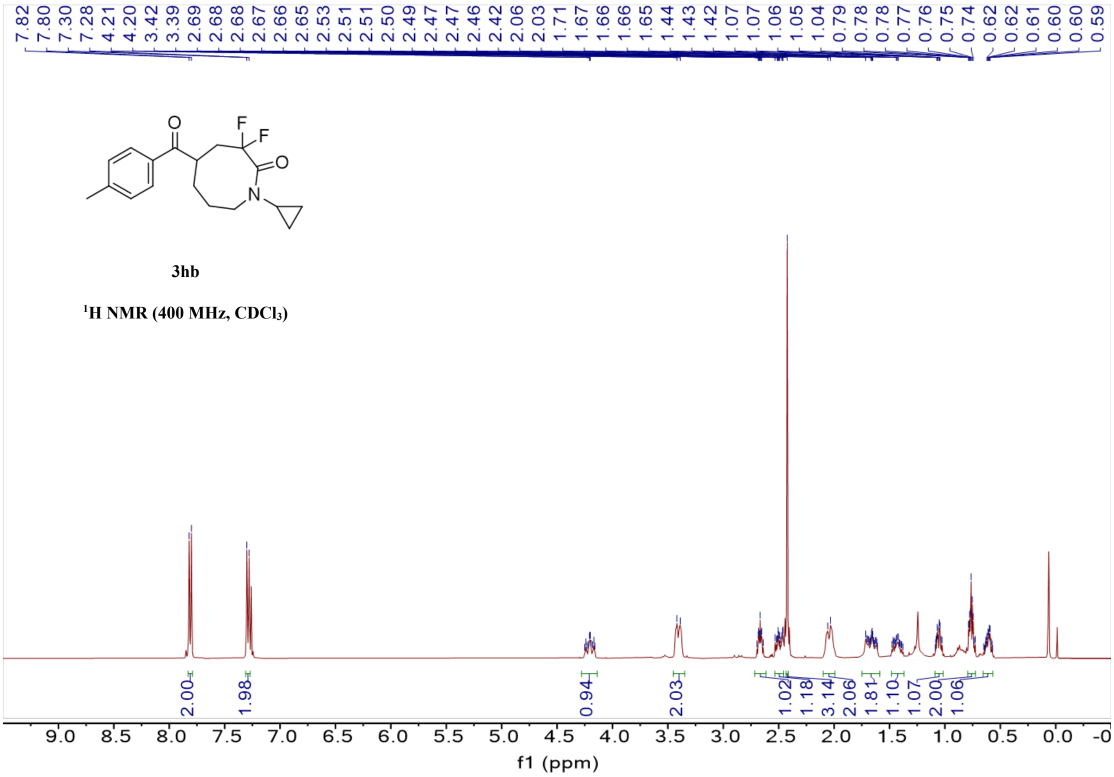

3hb

^{1}H NMR (400 MHz, $CDCl_3$)

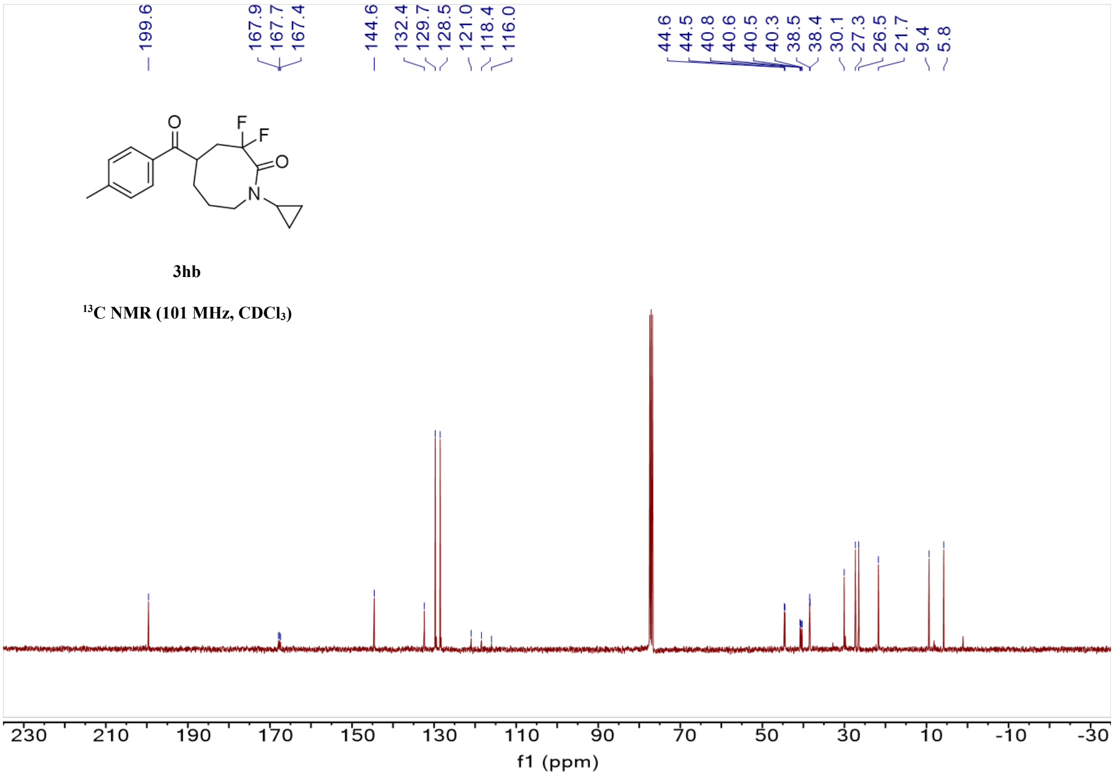

3hb

^{13}C NMR (101 MHz, $CDCl_3$)

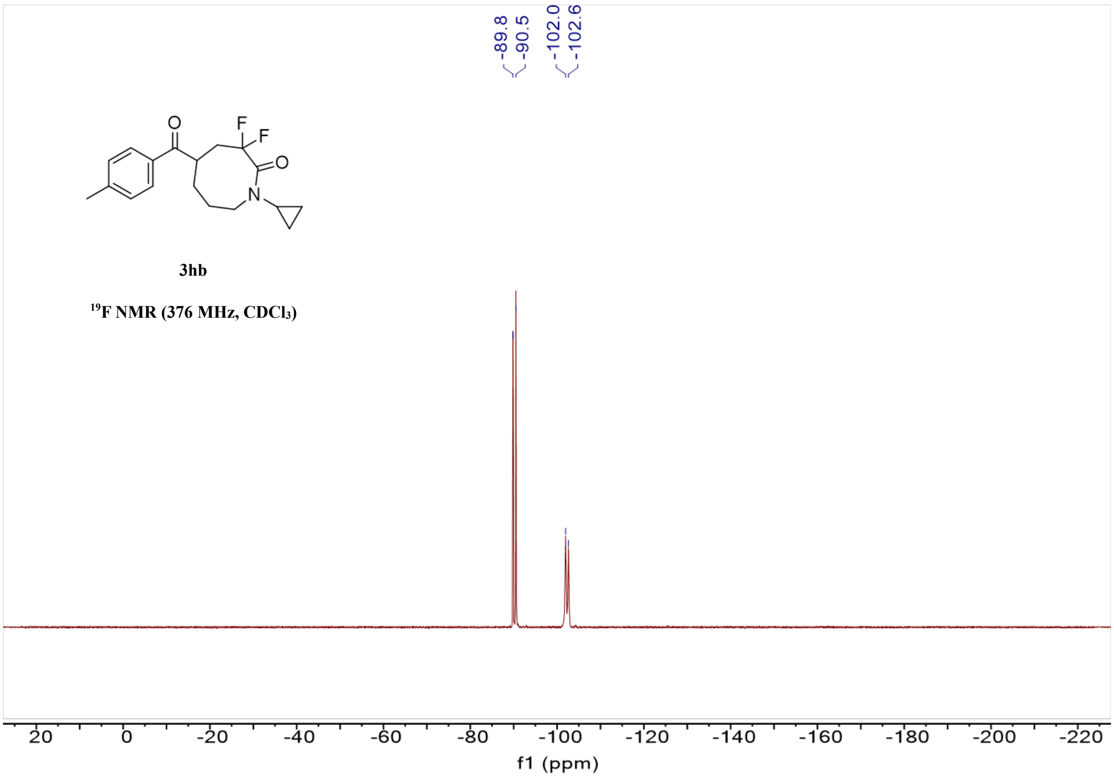

3hb

^{19}F NMR (376 MHz, CDCl$_3$)

8. Copy of DEPT-135 Spectra of 3hb.

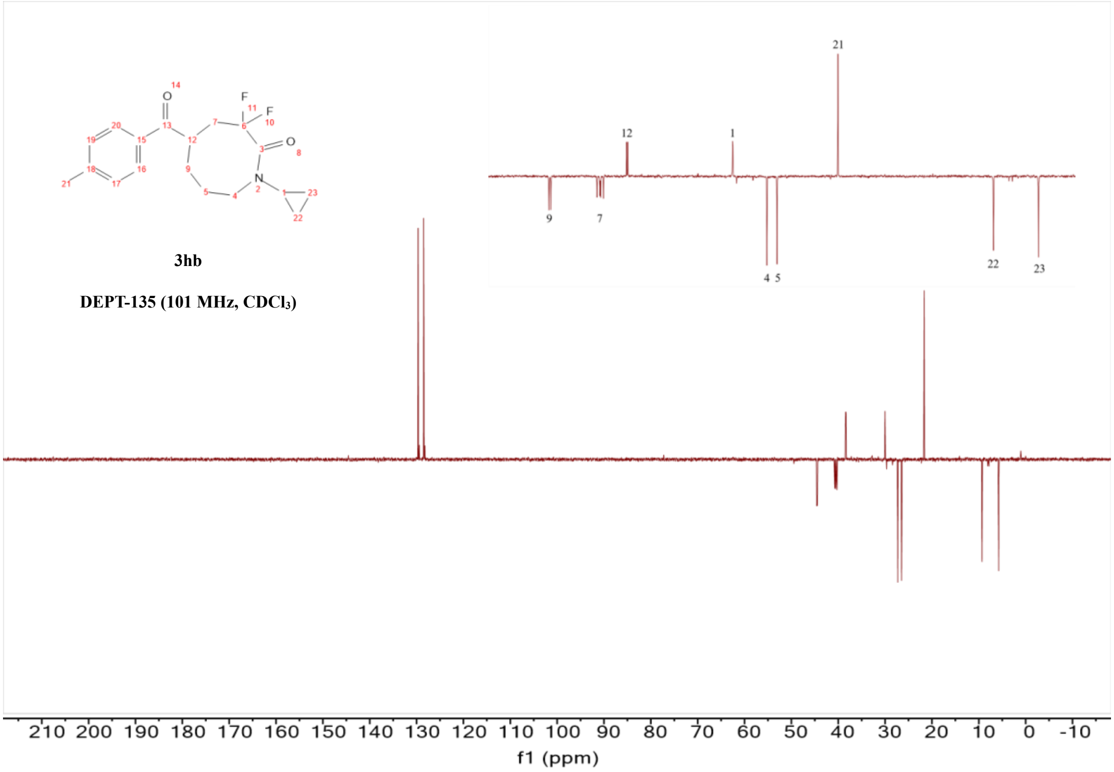

Supplement: Supplementary file 1 [file ol6c02643_si_001.pdf]
